# Epidemiology of herpes simplex virus type 2 in sub-Saharan Africa: Systematic review, meta-analyses, and meta-regressions

**DOI:** 10.1016/j.eclinm.2021.100876

**Published:** 2021-05-07

**Authors:** Manale Harfouche, Farah M. Abu-Hijleh, Charlotte James, Katharine J. Looker, Laith J. Abu-Raddad

**Affiliations:** a Infectious Disease Epidemiology Group, Weill Cornell Medicine-Qatar, Cornell University, Qatar Foundation - Education City, Doha, Qatar; b World Health Organization Collaborating Centre for Disease Epidemiology Analytics on HIV/AIDS, Sexually Transmitted Infections, and Viral Hepatitis, Weill Cornell Medicine–Qatar, Cornell University, Qatar Foundation – Education City, Doha, Qatar; c Department of Public Health, College of Health Sciences, Academic Quality Affairs Office, QU Health, Qatar University, Doha, Qatar; d Population Health Sciences, Bristol Medical School, University of Bristol, Bristol, United Kingdom; e Department of Population Health Sciences, Weill Cornell Medicine, Cornell University, New York, NY, United States

**Keywords:** Seroprevalence, Genital ulcer disease, Genital herpes, Synthesis, Region

## Abstract

**Background:**

Herpes simplex virus type 2 (HSV-2) infection is a prevalent, sexually transmitted infection with a sizable disease burden that is highest in sub-Saharan Africa. This study aimed to characterize HSV-2 epidemiology in this region.

**Methods:**

Cochrane and PRISMA guidelines were followed to systematically review, synthesize, and report HSV-2 related findings up to August 23, 2020. Meta-analyses and meta-regressions were conducted.

**Findings:**

From 218 relevant publications, 451 overall outcome measures and 869 stratified measures were extracted. Pooled incidence rates ranged between 2.4–19.4 per 100 person-years across populations. Pooled seroprevalence was lowest at 37.3% (95% confidence interval (CI): 34.9–39.7%) in general populations and high in female sex workers and HIV-positive individuals at 62.5% (95% CI: 54.8–70.0%) and 71.3% (95% CI: 66.5–75.9%), respectively. In general populations, pooled seroprevalence increased steadily with age. Compared to women, men had a lower seroprevalence with an adjusted risk ratio (ARR) of 0.61 (95% CI: 0.56–0.67). Seroprevalence has decreased in recent decades with an ARR of 0.98 (95% CI: 0.97–0.99) per year. Seroprevalence was highest in Eastern and Southern Africa. Pooled HSV-2 proportion in genital ulcer disease was 50.7% (95% CI: 44.7–56.8%) and in genital herpes it was 97.3% (95% CI: 84.4–100%).

**Interpretation:**

Seroprevalence is declining by 2% per year, but a third of the population is infected. Age and geography play profound roles in HSV-2 epidemiology. Temporal declines and geographic distribution of HSV-2 seroprevalence mirror that of HIV prevalence, suggesting sexual risk behavior has been declining for three decades. HSV-2 is the etiological cause of half of genital ulcer disease and nearly all genital herpes cases with limited role for HSV-1.

**Funding:**

This work was supported by pilot funding from the Biomedical Research Program at Weill Cornell Medicine in Qatar and by the Qatar National Research Fund [NPRP 9–040–3–008].

**Research in context**

**Evidence before this study**

Herpes simplex virus type 2 (HSV-2) infection is a highly prevalent sexually transmitted infection (STI) worldwide, and results in a sizable disease burden. Despite breadth of empirical evidence for this infection, a PubMed search using the search criteria ("Herpes Simplex" [MeSH] AND "Review" [Publication Type]) yielded no regional in-depth, robust systematic review and meta-analytical assessment regarding the epidemiology of this infection in sub-Saharan Africa or elsewhere.

**Added value of this study**

Based on a large body of identified evidence, the study provided estimates for HSV-2 incidence rate, seroprevalence, and proportion of HSV-2 detection in genital ulcer disease (GUD) and in genital herpes. The study also established epidemiological associations relevant for Africa and elsewhere. Seroprevalence was declining rapidly by 2% per year, yet still remains high with a third of the population infected. The study further documented HSV-2 as the etiological cause of half of GUD cases and nearly all of genital herpes cases in this part of the world.

**Implications of all the available evidence**

HSV-2 infection and its adverse health outcomes are widespread in sub-Saharan Africa, more so than any other region, yet despite this disease burden, this infection remains uncontrolled with the absence of any specific or targeted public health program to prevent and control genital herpes. In the context of serious consequences for reproductive, sexual, and psychosocial health, the study findings demonstrate an urgent need for both prophylactic and therapeutic HSV-2 vaccines, and argue for the acceleration of ongoing efforts to develop a vaccine. The findings further provide concrete evidence and analytical knowledge towards building a business case for the global public health value of HSV-2 vaccines.

Alt-text: Unlabelled box

## 1 Introduction

Herpes simplex virus type 2 (HSV-2) infection is a highly prevalent, sexually transmitted infection (STI) worldwide [1]. It is a leading cause of genital ulcer disease (GUD) and genital herpes, manifesting in the form of painful, recurrent, and frequent genital lesions [2], [3], [4], [5], [6], [7], [8]. Its vertical transmission from mother-to-child can lead to neonatal herpes, a severe and sometimes fatal outcome in newborns [3,9]. HSV-2 is linked to a 2- to 3-fold increase in sexual transmission and acquisition of HIV [10], [11], [12], implying potential epidemiological synergy between the two viruses [11,13,14].

HSV-2 is typically asymptomatic in most of those who acquire it [3]. Its chronic and reactivating nature, as well as its subclinical shedding [15,16], increase its rate of transmission and lead to high antibody prevalence (seroprevalence) among general and higher-risk populations alike [11,17,18]. Since HSV-2 is more transmissible than HIV and produces long-lasting antibodies, it has been used as an objective biological marker of sexual risk behavior and risk of HIV infection [19], [20], [21], [22], [23]. Analyses using empirical data and mathematical modeling supported the utility of using HSV-2 seroprevalence to predict HIV epidemic potential [19,20,24].

Inadequate understanding of HSV-2 epidemiology and HSV-2′s considerable consequences on sexual and reproductive health and the HIV epidemic [11,13], call for further research and urgent preventive and control measures. The World Health Organization (WHO) outlined a “Global Health Sector Strategy on STIs, 2016–2021″ [25] to eliminate STIs as a main public health concern by 2030 through integration of preventive and control measures. Consequently, WHO, along with its global partners, is spearheading efforts to develop an HSV vaccine as an urgent priority [26,27].

To inform these efforts, we conducted a systematic review to characterize HSV-2 infection levels and trends in sub-Saharan Africa (SSA), the hub of the HIV epidemic [28,29]. We estimated pooled means for each outcome measure (incidence rate, seroprevalence, proportion of HSV-2 in GUD, and proportion of HSV-2 in genital herpes) across populations and subpopulations. We also conducted meta-regression analyses to assess temporal trends and to identify predictors of high seroprevalence and between-study heterogeneity.

## 2 Methodology

### 2.1 Data sources and search strategy

This systematic review was guided by the Cochrane Collaboration Handbook [30] and was reported according to the Preferred Reporting Items for Systematic Reviews and Meta-analyses (PRISMA) guidelines [31]. The review was informed by the methodology applied recently in a series of systematic reviews for HSV-1 infection [32], [33], [34], [35], [36].

All available publications were systematically reviewed up to August 23, 2020. The search was conducted in PubMed and Embase databases, using search strategies with exploded MeSH/Emtree terms, broad search criteria, without language or date restrictions to widen the scope and include all subheadings (Table S1). The definition of Africa included 45 countries, as defined by WHO for the African Region [37], covering countries of sub-Saharan Africa. The list of countries and their subregional stratification is in Box S1.

### 2.2 Study selection and eligibility criteria

Search results were imported into the reference manager Endnote (Thomson Reuters, USA), whereby duplicate publications were identified and removed. The remaining records were screened for relevance based on titles and abstracts, followed by full text screening of relevant and potentially relevant records. Additional bibliography screening was performed on both reviews and the relevant articles to identify any missing publications.

Inclusion criteria were met if publications reported primary data on any of the following four outcomes: 1) HSV-2 incidence rate, 2) HSV-2 seroprevalence, 3) proportion of HSV-2 detection in GUD, and 4) proportion of HSV-2 detection in genital herpes. A sample size of ≥10 was required for inclusion for all outcome measures. Exclusion criteria encompassed case reports, series, commentaries, reviews, and qualitative studies. In this review, “publication” refers to a document reporting any outcome measure, whilst a “study” refers to details of a specific outcome measure. Special care was given to ensure that overlapping studies were only included once by searching the extraction database for any potential overlap in years of publication, years of data collection, study authors, and study locations.

### 2.3 Data extraction and synthesis

MH and FA each extracted half of the studies. Subsequently, MH double-extracted and reviewed studies extracted by FA and FA did the same for those extracted by MH. Discrepancies were discussed in consultation with LJA to reach consensus. A list of extracted variables is in Box S2.

Overall outcome measures and their stratified measures were extracted, provided that the stratification agreed with a pre-set stratification hierarchy and the subsample in each stratum was ≥10. The pre-set stratification hierarchy sequence for incidence and seroprevalence measures was as follows: population type (see Box S3 for definition), sex, and age. As for proportion of HSV-2 detection in GUD and genital herpes, the sequence was: genital herpes episode status (primary *versus* recurrent episode), sex, age, and study site (hospital *versus* outpatient clinic).

Measures reporting any HSV-2 outcome among children <15 years old were only reported, and not included in the analyses.

### 2.4 Quality assessment

Relevant studies were subjected to a quality pre-assessment to evaluate the validity of the assays used, given their limitations [38,39]. This assessment was done with the help of an expert from the University of Washington, Professor Rhoda Ashley-Morrow, who assessed the quality of study diagnostic methods. Only studies with valid, sensitive, and specific assays were included in the review. These studies were then evaluated using the Cochrane approach for risk of bias (ROB) assessment [30]. Study precision was classified as either low or high based on the study sample size (<100 *versus* ≥100) [40]. Studies were classified as either low or high ROB using two quality domains: sampling method (probability *versus* non-probability based sampling) and response rate (≥80% *versus* <80% or unclear). Effect of ROB on study outcome was investigated through meta-regression as noted below.

### 2.5 Meta-analyses

Pooled mean estimates for all four outcomes were calculated across all strata using meta-analyses, provided each stratum had ≥3 measures. Only studies reporting incidence rate by person-time were included in the meta-analyses. Log transformed incidence rates were used to calculate pooled estimates using the inverse variance method in the metarate function [41,42]. HSV-2 seroprevalence measures and proportions of HSV-2 detection in GUD and in genital herpes were each pooled using the DerSimonian-Laird random-effects model [41], applying the Freeman-Tukey double arcsine transformation to stabilize the variance [43], and factoring knowledge of the applicability of this transformation [44]. Random-effects meta-analyses were used to account for the potential existence of heterogeneity in effect size arising from clinical differences in study participants or study methodological differences (such as diagnostic method, sampling method, and response rate). Existence of heterogeneity in effect size was assessed using Cochran's Q statistic [41,45]. The magnitude of between-study variation attributed to *true* differences in effect size, as opposed to *chance*, was measured using I^2^ [41,45]. Distributions of *true* measures around the pooled mean were described using the prediction interval. Sources of heterogeneity were investigated through meta-regressions. Meta-analyses were conducted in R version 3.4.1 [46] using the meta package [42].

### 2.6 Meta-regressions

Univariable and multivariable random-effects meta-regression analyses were conducted using log-transformed seroprevalence measures to examine factors and predictors potentially associated with increased HSV-2 seroprevalence, as well as sources of between-study heterogeneity. Pre-set variables included in these analyses are listed in Box S4. Factors included in multivariable models had to have a p-value<0.1 in the univariable model. Strength of evidence for an association was deemed significant for factors with a p-value<0.05 in the multivariable models. Meta-regressions were conducted in Stata/SE version 13 [47] using the metareg package [48].

### 2.7 Role of the funding source

The funder of the study had no role in study design, data collection, data analysis, data interpretation, or writing of the article. The corresponding author had full access to all data in the study and had the final responsibility for the decision to submit for publication.

## 3 Results

### 3.1 Search results and scope of evidence

**Fig. 1:**
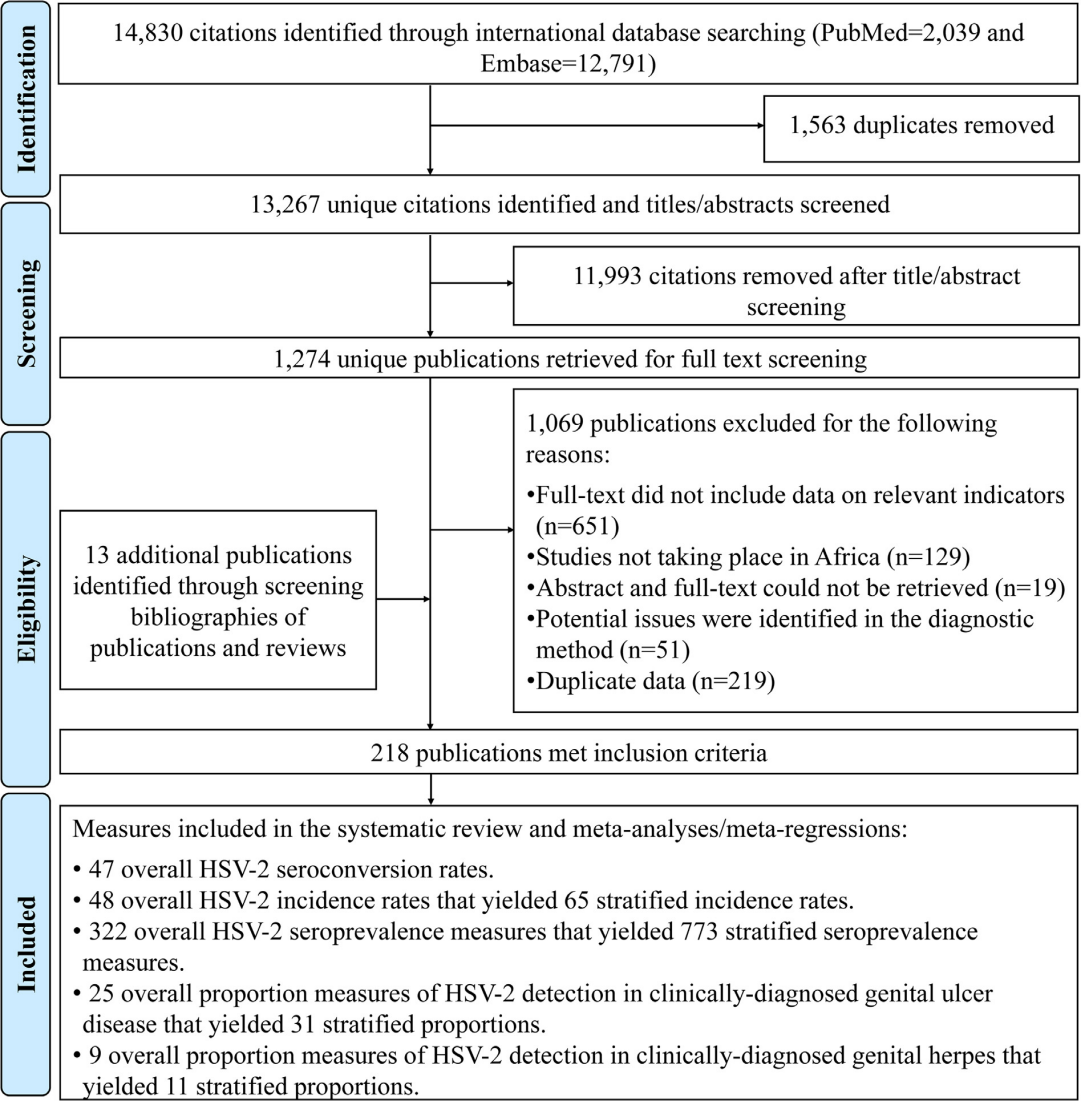
Flowchart of article selection for the systematic review of HSV-2 infection in sub-Saharan Africa, per PRISMA guidelines [31] Abbreviations: HSV-2 = Herpes simplex virus type 2.

Fig. 1 depicts the PRISMA flowchart describing the study selection process [31]. A total of 14,830 citations were captured in the initial search (2039 from PubMed and 12,791 from Embase). After deduplication, title and abstract screening, and full text screening, 205 relevant publications were identified. Thirteen additional publications, including country level reports, were identified through bibliography screening [49], [50], [51], [52], [53], [54], [55], [56], [57], [58], [59], [60], [61].

In total, 218 publications met the inclusion criteria. Extracted measures encompassed 47 seroconversion rates, 48 overall (65 stratified) incidence rates, 322 overall (773 stratified) seroprevalence measures, 25 overall (31 stratified) proportions of HSV-2 detection in GUD, and 9 overall (11 stratified) proportions of HSV-2 detection in genital herpes.

### 3.2 Incidence overview and pooled mean estimates for HSV-2 incidence rate

Table S2 summarizes extracted seroconversion and incidence rates. Studies were either longitudinal cohorts (number of measures (n)=35; 49.3%) or randomized controlled trials (*n* = 36; 50.7%), with a follow-up duration ranging between 6 weeks to 6 years. HSV-2 seroconversion rates (*n* = 47), that is cumulative incidence, ranged between 0.0–58.0% across studies, reflecting in part the widely variable duration of follow-up.

In general populations, HSV-2 incidence rates among women (*n* = 20) ranged from 3.6 to 21.7 per 100 person-years with a median of 7.5 and a pooled mean of 7.2 (95% confidence interval (CI): 5.5–9.4) per 100 person-years (Table 1). Among men (*n* = 20), incidence rate ranged from 1.5 to 10.5 per 100 person-years with a median of 5.5 and a pooled mean of 4.1 (95% CI: 3.1–5.3) per 100 person-years. Higher incidence rates were found in higher risk populations. A summary of pooled mean incidence rate by population type and associated forest plots are in Table 1 and Figure S1, respectively. Seroconversion rates were not pooled because follow-up durations varied so widely that rates were methodologically not comparable.

**Table 1:** Pooled mean estimates for herpes simplex virus type 2 incidence rate among different populations in sub-Saharan Africa.

Population type	Outcome measures	Samples	HSV-2 incidence rate (per 100 person-years)		Pooled mean HSV-2 incidence rate	Heterogeneity measures	
	Total n	Total	Range	Median	Mean (per 100 person-years)	Q*	I²† (%)
		person-years			(95% CI)	(p-value)	(95% CI)
General populations							
Women	20	21,607.4	3.6–21.7	7.5	7.2 (5.5–9.4)	321.1 (*p*<0.001)	94.1 (92.1–95.6)
<25 years old	6	6914.5	4.0–21.7	9.1	6.7 (4.6–9.7)	51.5 (*p*<0.001)	90.3 (81.6–94.9)
≥25 years old	3	154.0	9.0–15.4	9.3	11.4 (7.1–18.3)	1.1 (*p* = 0.582)	0.0 (0.0–80.8)
Mixed ages	11	14,538.9	3.6–21.0	7.0	6.9 (4.7–10.1)	257.5 (*p*<0.001)	96.1 (94.5–97.3)
Men	20	29,287.3	1.5–10.5	5.5	4.1 (3.1–5.3)	250.9 (*p*<0.001)	92.4 (89.7–94.4)
<25 years old	6	3841.1	1.5–10.4	6.5	4.0 (2.3–7.0)	49.9 (*p*<0.001)	90.0 (80.9–94.7)
≥25 years old	8	1835.2	4.0–10.5	6.5	6.5 (5.3–7.9)	7.7 (*p* = 0.364)	8.6 (0.0–70.4)
Mixed age	6	23,611.1	1.4–4.9	5.0	2.4 (1.6–3.6)	120.0 (*p*<0.001)	95.8 (93.1–97.5)
Mixed sexes	5	15,603.7	2.3–3.6	3.5	3.2 (2.5–3.7)	17.9 (*p* = 0.001)	77.7 (46.2–90.7)
Intermediate-risk populations							
Women	3	774.8	14.2–28.6	17.3	19.4 (11.8–32.1)	16.8 (*p*<0.001)	88.1 (66.9–95.7)
Higher-risk populations							
Female sex workers	3	1458.0	8.7–23.0	21.0	18.0 (13.1–24.9)	11.2 (*p* = 0.004)	82.2 (45.2–94.2)
HIV-negative populations							
Mixed sexes	5	5203.0	5.2–11.0	6.1	7.9 (6.0–10.5)	31.7 (*p*<0.001)	87.4 (73.0–94.1)
HIV-positive individuals and in individuals in HIV discordant couples							
Mixed sexes	3	2606.8	5.5–14.8	7.7	8.6 (4.7–15.7)	38.2 (*p*<0.001)	94.8 (88.1–97.7)
Other populations							
Mixed sexes	6	767.3	3.4–35.1	17.1	14.2 (8.6–23.6)	27.7 (*p*<0.001)	82.0 (61.6–91.5)

⁎ Q: the Cochran's Q statistic is a measure assessing the existence of heterogeneity in incidence rates.

† I^2^: a measure that assesses the magnitude of between-study variation that is due to actual differences in incidence rates across studies rather than chance. Abbreviations: CI = Confidence interval, HIV = human immunodeficiency virus, HSV-2 = Herpes simplex virus type 2.

### 3.3 Prevalence overview

Tables S3, S4, S5, S6, and S7 summarize overall seroprevalence measures (*n* = 322) across the subregions of SSA. The earliest publication appeared in 1991. Half the studies were published after the year 2010 (*n* = 176; 55.5%), and most were based on convenience sampling (*n* = 233; 72.4%).

For stratified seroprevalence measures, HSV-2 seroprevalence among general population women (*n* = 290) ranged from 0.1 to 97.4% with a median of 43.1%, and among general population men (*n* = 186) from 0.0 to 84.2% with a median of 27.5% (Table 2).

**Table 2:** Pooled mean estimates for herpes simplex virus type 2 seroprevalence among the different at risk populations in sub-Saharan Africa, by sex.

Population type	Outcome measures	Sample size	HSV-2 seroprevalence (%)		Pooled mean HSV-2 seroprevalence	Heterogeneity measures		
	Total n	Total N	Range	Median	Mean (%) (95% CI)	Q* (p-value)	I²† (%) (95% CI)	Prediction interval‡ (%)
General populations	507	230,541	0.0–97.4	39.5	37.3 (34.9–39.7)	73,247.9 (*p*<0.001)	99.3 (99.3–99.3)	0.7–88.0
Women	290	134,034	0.1–97.4	43.1	43.1 (39.8–46.5)	44,291.3 (*p*<0.001)	99.3 (99.3–99.4)	1.8–92.4
Men	186	83,898	0.0–84.2	27.5	29.1 (25.7–32.6)	21,577.1 (*p*<0.001)	99.1 (99.1–99.2)	0.1–77.7
Mixed	31	12,609	1.3–90.9	33.9	32.5 (27.3–37.8)	975.5 (*p*<0.001)	96.9 (96.3–97.4)	8.4–62.9
Intermediate-risk populations	45	9259	0.0–92.2	58.0	57.1 (50.1–63.9)	1906.5 (*p*<0.001)	97.7 (97.3–98.0)	13.7–94.6
Women	29	8267	20.5–92.2	70.6	66.4 (59.1–73.3)	1283.4 (*p*<0.001)	97.8 (97.4–98.2)	25.4–96.4
Men	13	849	9.1–88.5	43.0	43.6 (31.4–56.2)	150.1 (*p*<0.001)	92.0 (88.1–94.6)	4.3–88.7
Mixed	3	143	0.0–45.0	21.0	18.2 (0.5–43.4)	29.1 (*p*<0.001)	93.1 (83.3–97.2)	0.0–100
Higher-risk populations	40	13,476	4.3–99.0	63.7	61.4 (53.4–69.1)	3264.9 (*p*<0.001)	98.8 (98.7–98.9)	13.4–98.2
FSWs	39	13,036	4.3–99.0	65.0	62.5 (54.8–70.0)	2898.0 (*p*<0.001)	98.7 (98.5–98.8)	15.7–97.9
MSM	1§	440	–	–	22.3 (18.5–26.3)§	-	-	-
HIV-negative populations	51	38,533	6.0–89.0	44.0	47.4 (43.2–51.5)	3198.9 (*p*<0.001)	98.4 (98.2–98.6)	19.7–75.9
Women	34	33,699	17.0–89.0	46.5	52.1 (47.5–56.7)	2283.9 (*p*<0.001)	98.6 (98.3–98.7)	25.6–78.0
Men	13	3119	11.0–80.4	31.6	37.9 (30.2–46.0)	227.1 (*p*<0.001)	94.7 (92.5–96.3)	10.9–69.8
Mixed	4	1715	6.0–57.9	35.6	35.8 (23.0–49.8)	84.4 (*p*<0.001)	96.4 (93.5–98.1)	0.0–93.1
HIV-positive individuals and in individuals in HIV discordant couples	42	15,521	17.5–95.2	70.2	71.3 (66.5–75.9)	1514.7 (*p*<0.001)	97.3 (96.8–97.7)	38.9–94.9
Women	20	6183	42.0–95.2	79.7	76.7 (70.1–82.7)	587.4 (*p*<0.001)	96.8 (95.9–97.5)	43.2–97.9
Men	15	4306	44.0–94.2	61.4	72.0 (63.7–79.6)	311.0 (*p*<0.001)	95.5 (93.9–96.7)	36.2–96.8
Mixed	7	5032	17.5–70.2	62.3	54.6 (47.4–61.8)	108.9 (*p*<0.001)	94.5 (91.0–96.6)	31.0–77.1
STI clinic attendees and symptomatic populations	72	11,996	14.7–93.3	60.7	61.2 (56.5–65.9)	1826.8 (*p*<0.001)	96.1 (95.6–96.6)	22.9–93.0
Women	26	4038	38.0–93.3	75.3	69.2 (62.7–75.4)	393.3 (*p*<0.001)	93.6 (91.8–95.1)	35.2–94.7
Men	38	6585	14.7–86.4	56.6	52.9 (46.9–58.8)	791.4 (*p*<0.001)	95.3 (94.3–96.1)	18.8–85.6
Mixed	8	1373	85.0–85.0	79.4	75.8 (68.5–82.5)	35.9 (*p*<0.001)	80.5 (62.3–89.9)	51.8–93.7
Other populations	16	6506	11.2–89.4	48.1	50.3 (41.9–58.7)	546.4 (*p*<0.001)	97.3 (96.5–97.9)	17.4–58.7
Women	9	5642	27.8–89.4	48.7	53.8 (45.6–61.9)	260.2 (*p*<0.001)	96.9 (95.6–97.9)	24.8–81.5
Men	3	803	11.2–38.6	23.7	23.4 (10.5–39.4)	48.2 (*p*<0.001)	95.9 (91.0–98.1)	0.0–100
Mixed	4	61	46.7–83.3	75.7	71.1 (54.4–85.5)	5.1 (*p* = 0.164)	41.2 (0.0–80.2)	13.7–100

⁎ Q: the Cochran's Q statistic is a measure assessing the existence of heterogeneity in seroprevalence.

† I^2^: a measure that assesses the magnitude of between-study variation that is due to actual differences in seroprevalence across studies rather than chance.

‡ Prediction interval: a measure that estimates the distribution (95% interval) of true seroprevalence around the estimated mean.

§ No meta-analysis was done due to the small number of studies (*n* < 3). ^¶^Symptomatic populations include patients with clinical manifestations related to an STI. Abbreviations: CI = Confidence interval, FSWs = Female sex workers, HIV = Human immunodeficiency virus, HSV-2 = Herpes simplex virus type 2, MSM = Men who have sex with men, STI = Sexually transmitted disease.

In higher-risk populations (*n* = 40), almost all studies were conducted among female sex workers (FSWs; *n* = 39) with seroprevalence ranging from 4.3 to 99.0% with a median of 65.0% (Table 2). High seroprevalence was observed in HIV-positive individuals and in individuals in HIV discordant couples, ranging from 42.0 to 95.2% with a median of 79.7% among women (*n* = 20), and from 44.0 to 94.2% with a median of 61.4% among men (*n* = 15).

Tables 2, 3, and S9 summarize HSV-2 seroprevalence measures for additional populations and subpopulations, including by population type, country, subregion, age, sex, and year of publication.

**Table 3:** Pooled mean estimates for herpes simplex virus type 2 seroprevalence among general populations in sub-Saharan Africa.

Population classification	Outcome measures	Sample size	HSV-2 seroprevalence (%)		Pooled mean HSV-2 seroprevalence	Heterogeneity measures		
	Total n	Total N	Range	Median	Mean (%) (95% CI)	Q* (p-value)	I²† (%) (95% CI)	Prediction interval‡ (%)
**African countries**								
Benin	16	3991	1.0–57.0	28.0	24.6 (17.1–32.9)	447.1 (*p*<0.001)	96.6 (95.6–74.4)	1.1–63.5
Burkina Faso	14	5932	4.1–40.5	20.3	19.5 (14.7–24.8)	297.3 (*p*<0.001)	95.6 (94.0–96.8)	3.5–43.8
Cameroon	23	2309	3.0–90.9	59.0	53.6 (40.9–66.2)	804.9 (*p*<0.001)	97.3 (96.6–97.8)	2.4–99.6
Ethiopia	10	2444	7.9–59.5	28.5	27.3 (16.8–39.3)	277.4 (*p*<0.001)	96.8 (95.4–97.7)	0.2–73.7
Kenya	71	34,605	1.9–91.3	42.4	39.9 (34.3–45.9)	8018.6 (*p*<0.001)	99.1 (99.1–99.2)	3.0–86.2
Malawi	55	9571	0.0–87.5	50.0	45.1 (38.2–52.0)	2192.8 (*p*<0.001)	97.5 (97.2–97.8)	4.5–90.2
Nigeria	14	2205	8.7–61.3	29.3	28.5 (16.1–42.7)	486.9 (*p*<0.001)	97.3 (96.5–98.0)	0.0–85.6
South Africa	79	44,449	1.5–92.5	31.0	34.1 (27.3–41.9)	19,846.8 (*p*<0.001)	99.6 (99.6–99.6)	0.0–93.4
Tanzania	41	18,731	7.0–68.0	36.8	37.4 (33.7–41.1)	1008.2 (*p*<0.001)	96.0 (95.3–96.7)	16.1–61.6
Uganda	59	52,216	9.9–90.7	53.4	50.5 (44.2–56.8)	12,120.9 (*p*<0.001)	99.5 (99.5–99.6)	8.5–92.1
Zambia	42	25,973	1.0–80.0	41.5	36.3 (28.2–44.8)	7909.4 (*p*<0.001)	99.5 (99.4–99.5)	0.3–88.7
Zimbabwe	42	20,530	0.1–71.0	30.6	26.0 (17.5–35.5)	8678.9 (*p*<0.001)	99.5 (99.5–99.6)	0.0–88.6
Other countries§	41	7585	2.6–97.4	22.2	31.1 (24.5–38.1)	1564.8 (*p*<0.001)	97.4 (97.0–97.8)	0.1–77.3
**African subregions**								
Eastern Africa	188	110,399	1.9–91.3	42.4	41.9 (38.4–45.3)	25,111.0 (*p*<0.001)	99.3 (99.2–99.3)	4.9–85.6
Southern Africa	226	100,925	0.0–92.5	40.3	35.8 (31.6–40.0)	43,422.5 (*p*<0.001)	99.5 (99.5–99.5)	0.0–92.9
Western Africa	68	16,005	1.0–97.4	21.9	25.0 (20.9–29.4)	2442.9 (*p*<0.001)	97.3 (96.9–97.6)	1.2–63.7
Central Africa	25	3212	3.0–90.9	59.0	52.4 (40.4–64.3)	1065.0 (*p*<0.001)	97.7 (97.3–98.1)	2.6–98.9
**Age group**								
<20 years	88	40,217	0.0–46.0	11.0	12.4 (10.6–14.3)	2419.1 (*p*<0.001)	96.4 (96.0–96.8)	1.0–32.6
20–30 years	112	49,099	2.9–91.3	36.8	34.2 (30.7–37.8)	7583.3 (*p*<0.001)	98.5 (98.4–98.6)	5.0–72.7
30–40 years	69	26,412	16.4–90.4	60.6	57.1 (54.8–63.3)	3210.2 (*p*<0.001)	97.9 (97.6–98.1)	24.6–89.2
40–50 years	48	12,470	20.0–92.5	61.7	64.6 (59.5–69.5)	1445.1 (*p*<0.001)	96.7 (96.2–97.2)	29.8–92.5
>50 years	15	4054	39.6–80.9	60.6	58.2 (50.3–65.9)	321.3 (*p*<0.001)	95.6 (94.1–96.8)	26.3–86.8
Mixed	175	98,289	0.1–97.4	35.0	36.2 (32.1–40.3)	31,073.2 (*p*<0.001)	99.4 (99.4–99.5)	0.4–87.7
**Year of publication category**								
≤2005	124	27,633	1.0–90.9	45.5	42.1 (38.1–46.2)	5477.8 (*p*<0.001)	97.8 (97.6–97.9)	6.2–83.8
2005–2015	283	142,060	0.0–97.4	38.4	35.7 (32.6–38.9)	42,499.0 (*p*<0.001)	99.3 (99.3–99.3)	0.6–86.0
>2015	100	60,848	1.5–92.5	35.8	35.9 (30.1–41.8)	23,039.3 (*p*<0.001)	99.6 (99.5–99.6)	0.0–90.9
**All studies**	**507**	**230,541**	**0.0–97.4**	**39.5**	**37.3 (34.9–39.7)**	**73,247.9 (*p*<0.001)**	**99.3 (99.3–99.3)**	**0.7–88.0**

⁎ Q: the Cochran's Q statistic is a measure assessing the existence of heterogeneity in seroprevalence.

† I^2^: a measure that assesses the magnitude of between-study variation that is due to actual differences in seroprevalence across studies rather than chance.

‡ Prediction interval: a measure that estimates the distribution (95% interval) of true seroprevalence around the estimated mean.

§ Other countries: Chad, Cote D'Ivoire, Eritrea, Gabon, Gambia, Ghana, Mali, Namibia, Rwanda, Senegal. Abbreviations: CI = Confidence interval, HSV-2 = Herpes simplex virus type 2.

### 3.4 Pooled mean estimates for HSV-2 seroprevalence

Tables 2, 3, and S8 show results of seroprevalence meta-analyses across populations and subpopulations. Pooled mean seroprevalence was lowest at 37.3% (95% CI: 34.9–39.7%) in general populations (*n* = 507), followed by 47.4% (95% CI: 43.2–51.5%) in HIV-negative populations (*n* = 51), 57.1% (95% CI: 50.1–63.9%) in intermediate-risk populations (*n* = 45), 61.2% (95% CI: 56.5–65.9%) in STI clinic attendees and symptomatic populations (*n* = 72), 61.4% (95% CI: 53.4–69.1%) in higher-risk populations (*n* = 40; mainly FSWs), and 71.3% (95% CI: 66.5–75.9%) in HIV-positive individuals and in individuals in HIV discordant couples (*n* = 42; Table 2).

Among general populations, the pooled mean seroprevalence varied across African subregions (Table 3), with the lowest being 25.0% (95% CI: 20.9–29.4%) in Western Africa (*n* = 68), followed by 35.8% (95% CI: 31.6–40.0%) in Southern Africa (*n* = 226), 41.9% (95% CI: 38.4–45.3%) in Eastern Africa (*n* = 188), and 52.4% (95% CI: 40.4–64.3%) in Central Africa (*n* = 25). A similar pattern was observed across subregions for women and men, with women having consistently higher pooled mean seroprevalences than men (Table S8).

Across age groups (Table 3), pooled mean seroprevalence increased gradually from 12.4% (95% CI: 10.6–14.3%) in those <20 years-old (*n* = 88), followed by 34.2% (95% CI: 30.7–37.8%) in those 20–30 years-old (*n* = 112), 57.1% (95% CI: 54.8–63.3%) in those 30–40 years-old (*n* = 69), 64.6% (95% CI: 59.5–69.5%) in those 40–50 years-old (*n* = 48), and then decreasing slightly to 58.2% (95% CI: 50.3–65.9%) in those >50 years-old. A similar trend was observed across age groups for women and men separately, but seroprevalence grew faster with age for young women (Table S8).

Heterogeneity was evident in almost all meta-analyses (p-value<0.001), and was confirmed by wide prediction intervals (Tables 2, 3, and S8). Most heterogeneity was attributed to true variation in seroprevalence rather than to chance (I²>50%). The Q statistic was very large for some pooled measures (>10,000), indicating very strong evidence for heterogeneity in seroprevalence. Forest plots for meta-analyses across African subregions stratified by population type are in Figure S2.

### 3.5 Predictors of HSV-2 seroprevalence

Table 4 shows results of the meta-regression analyses for HSV-2 seroprevalence. Nine variables were eligible for inclusion in the multivariable model (p-value<0.1 in univariable analysis). Two multivariable models were conducted, one including year of publication as a categorical variable and one including it as a linear term.

**Table 4:** Univariable and multivariable meta-regression analyses for herpes simplex virus type 2 seroprevalence in sub-Saharan Africa.

			Outcome measures	Sample size	Univariable analysis				Multivariable analysis			
			Total n	Total N	*RR* (95%CI)	p-value	LR test p-value	Adjusted R^2^ (%)	Model 1*		Model 2†	
									*ARR* (95% CI)	p-value	*ARR* (95% CI)	p-value
**Population characteristics**	**Population type**	General populations	507	230,541	1.00	–	<0.001	12.9	1.00	–	1.00	–
		Intermediate-risk populations	45	9259	1.73 (1.35–2.21)	<0.001			1.50 (1.24–1.81)	<0.001	1.49 (1.23–1.81)	<0.001
		Higher-risk populations	40	13,476	1.70 (1.31–2.20)	<0.001			1.58 (1.28–1.94)	<0.001	1.59 (1.29–1.96)	<0.001
		HIV negative populations	51	38,533	1.41 (1.12–1.78)	0.003			1.31 (1.07–1.59)	0.008	1.30 (1.07–1.59)	0.008
		HIV positive individuals and individuals in HIV discordant couples	42	15,521	2.18 (1.70–2.80)	<0.001			2.17 (1.78–2.65)	<0.001	2.18 (1.78–2.66)	<0.001
		STI clinic attendees and symptomatic populations	72	11,996	1.88 (1.54–2.28)	<0.001			1.76 (1.49–2.08)	<0.001	1.78 (1.51–2.10)	<0.001
		Other populations	16	6506	1.53 (1.03–2.27)	0.033			1.22 (0.90–1.65)	0.179	1.24 (0.92–1.68)	0.150
	**Age group**	<20 years	108	42,984	1.00	–	<0.001	32.6	1.00	–	1.00	–
		20–30 years	144	55,344	2.49 (2.07–3.00)	<0.001			2.51 (2.15–2.93)	<0.001	2.53 (2.17–2.94)	<0.001
		30–40 years	84	27,875	4.26 (3.46–5.24)	<0.001			4.44 (3.74–5.28)	<0.001	4.46 (3.75–5.30)	<0.001
		40–50 years	52	12,650	4.62 (3.64–5.86)	<0.001			5.27 (4.32–6.44)	<0.001	5.27 (4.31–6.43)	<0.001
		>50 years	16	4127	4.30 (2.96–6.24)	<0.001			4.80 (3.52–6.56)	<0.001	4.64 (3.40–6.33)	<0.001
		Mixed ages	369	182,852	3.10 (2.64–3.64)	<0.001			2.37 (2.05–2.73)	<0.001	2.38 (2.07–2.75)	<0.001
	**Sex**	Women	447	204,899	1.00	–	<0.001	4.7	1.00	–	1.00	–
		Men	269	100,000	0.68 (0.60–0.78)	<0.001			0.61 (0.56–0.67)	<0.001	0.61 (0.56–0.67)	<0.001
		Mixed sexes	57	20,933	0.89 (0.70–1.12)	0.337			0.78 (0.65–0.94)	0.011	0.80 (0.66–0.96)	0.018
	**African subregion**	Eastern Africa	298	144,196	1.00	–	<0.001	3.3	1.00	–	1.00	–
		Southern Africa	319	128,395	0.82 (0.72–0.94)	0.006			0.85 (0.76–0.96)	0.014	0.86 (0.76–0.97)	0.015
		Western Africa	94	21,301	0.63 (0.52–0.77)	<0.001			0.60 (0.52–0.70)	<0.001	0.61 (0.53–0.71)	<0.001
		Central Africa	48	5832	1.01 (0.78–1.31)	0.909			0.75 (0.61–0.93)	0.009	0.75 (0.61–0.93)	0.009
		Mixed regions	14	26,225	1.23 (0.79–1.91)	0.347			1.01 (0.47–2.17)	0.965	1.02 (0.47–2.18)	0.955
	**National income**	LIC	326	123,156	1.00	–	0.027	0.7	1.00	–	1.00	–
		LMIC	285	107,905	0.86 (0.75–0.98)	0.030			0.95 (0.86–1.05)	0.385	0.96 (0.87–1.07)	0.540
		UMIC	145	63,908	0.89 (0.75–1.05)	0.195			1.12 (0.96–1.31)	0.131	1.14 (0.97–1.33)	0.093
		Mixed	17	30,863	1.35 (0.90–2.03)	0.141			0.84 (0.42–1.67)	0.622	0.86 (0.43–1.71)	0.670
**Study methodology characteristics**	**Assay type**	Western Blot	82	19,787	1.00	–	0.089	0.5	1.00	–	1.00	–
		ELISA	681	304,639	0.86 (0.71–1.05)	0.157			1.02 (0.87–1.20)	0.730	1.03 (0.88–1.21)	0.663
		Rapid test	10	1406	0.55 (0.31–0.97)	0.041			0.71 (0.46–1.08)	0.111	0.74 (0.48–1.12)	0.162
	**Sample size**‡	≥100	740	324,163	1.00	–	0.634	0.0	–	–	–	–
		<100	33	1669	1.07 (0.79–1.45)	0.654			–	–	–	–
	**Sampling method**	Probability based	311	165,963	1.00	–	<0.001	3.6	1.00	–	1.00	–
		Non-probability based	462	159,869	1.35 (1.20–1.53)	<0.001			1.06 (0.94–1.19)	0.289	1.06 (0.94–1.18)	0.303
	**Response rate**	≥80%	255	142,489	1.00	–	<0.001	4.0	1.00	–	1.00	–
		<80%	153	57,722	0.95 (0.80–1.12)	0.564			1.18 (1.04–1.34)	0.007	1.19 (1.05–1.35)	0.007
		Unclear	365	125,621	1.34 (1.17–1.53)	<0.001			1.30 (1.16–1.45)	<0.001	1.29 (1.15–1.44)	<0.001
**Temporal variables**	**Year of publication category**	<2005	218	38,609	1.00	–	<0.001	3.1	1.00	–	–	–
		2006–2015	422	208,948	0.86 (0.74–0.98)	0.032			0.85 (0.76–0.96)	0.009	–	–
		>2015	133	78,275	0.67 (0.56–0.80)	<0.001			0.80 (0.69–0.93)	0.005	–	–
	**Year of publication**		773	325,832	0.97 (0.97–0.98)	<0.001	<0.001	2.9	–	–	0.98 (0.97–0.99)	0.006

⁎ Variance explained by multivariable model 1 (adjusted *R^2^*) = 57.47%.

† Variance explained by multivariable model 2 (adjusted *R^2^*) = 57.50%.

‡ Sample size denotes the sample size of each study population found in the original publication. Abbreviations: *ARR* = Adjusted risk ratio, CI = Confidence interval*,* ELISA = Enzyme-linked immunosorbent type-specific assay, HIV = Human immunodeficiency virus, HSV-2 = Herpes simplex virus type 2, LIC = Low-income country, LMIC = Lower-middle-income country, LR = Likelihood ratio, *RR* = Risk ratio, STI = Sexually transmitted infection, UMIC = Upper-middle-income country.

The model including year of publication as a categorical variable explained 57.5% of seroprevalence variation and included population type, age group, sex, African subregion, national income, assay type, sampling method, response rate, and year of publication category (Table 4). Compared to general populations that had the lowest seroprevalence, seroprevalence was highest in HIV-positive individuals and in individuals in HIV discordant couples [adjusted risk ratio (ARR) of 2.17 (95% CI: 1.78–2.65)], followed by STI clinic attendees and symptomatic populations, higher-risk populations, intermediate-risk populations, and HIV-negative populations.

Compared to women, men had a 0.61-fold (95% CI: 0.56–0.67) lower seroprevalence. Seroprevalence increased rapidly with age among young people, but the increase plateaued by age 40–50 years. Seroprevalence was highest in Eastern Africa, followed by Southern Africa, Central Africa, and lowest in Western Africa. National income was not associated with seroprevalence.

Studies that had a lower or unknown response rate had a higher seroprevalence. Meanwhile, assay type, study sample size, and study sampling method were not associated with seroprevalence.

Compared to those published before the year 2005, studies published from 2006 to 2015 [ARR of 0.85 (95% CI: 0.76–0.96)] and after 2015 [ARR of 0.80 (95% CI: 0.69–0.93)] had lower seroprevalence.

The model including year of publication as a linear term (Table 4) showed similar results and indicated declining seroprevalence with time [ARR of 0.98 (95% CI: 0.97–0.99)]. The model explained 57.5% of seroprevalence variation.

Sensitivity analyses including year of data collection as a categorical variable or as a linear term (replacing year of publication) yielded similar results (Table S9).

### 3.6 Overview and meta-analyses of HSV-2 isolation in genital ulcer disease and in genital herpes

Table S10 summarizes extracted proportions of HSV-2 detected in GUD and in genital herpes. In GUD cases (*n* = 31), the proportion of HSV-2 ranged from 8.3 to 100% with a median of 49.1% and a pooled mean proportion of 50.7% (95% CI: 44.7–56.8%) (Table 5). Among women (*n* = 8), it ranged from 35.0 to 100% with a median of 49.5% and a pooled proportion of 59.0% (95% CI: 44.7–72.6%), and among men (*n* = 13), it ranged from 8.3 to 72.2% with a median of 49.1% and a pooled proportion of 47.3% (95% CI: 37.2–57.5%).

**Table 5:** Pooled proportions of herpes virus type 2 (HSV-2) virus isolation in clinically diagnosed GUD and in clinically diagnosed genital herpes in Africa.

Population type	Outcome measures	Sample size	Proportion of HSV-2 isolation (%)		Pooled proportion of HSV-2 isolation (%)	Heterogeneity measures		
	Total n	Total N	Range	Median	Mean (95% CI)	Q* (p-value)	I²† (%) (95% CI)	Prediction Interval‡ (%)
Patients with GUD	31	4296	8.3–100	49.1	50.7 (44.7–56.8)	423.6 (*p*<0.001)	92.9 (91.0–94.4)	19.7–81.5
Women	8	816	35.0–100	49.5	59.0 (44.7–72.6)	88.1 (*p*<0.001)	92.1 (86.7–95.2)	13.0–100
Men	13	1915	8.3–72.2	49.1	47.3 (37.2–57.5)	225.1 (*p*<0.001)	94.7 (92.4–96.2)	11.4–84.8
Mixed sexes	10	1565	22.4–73.0	50.0	49.1 (39.0–59.2)	110.3 (*p*<0.001)	91.8 (87.1–94.8)	15.5–83.2
Patients with genital herpes	11	1380	53.0–100	100	97.3 (84.4–100)	586.5 (*p*<0.001)	98.3 (97.8–98.7)	22.8–100
Men	6	715	53.0–100	100	97.3 (73.4–100)	318.8 (*p*<0.001)	98.4 (97.7–98.9)	0.0–100
Mixed sexes	5	665	91.4–100	85.4	97.8 (91.9–100)	23.9 (*p*<0.001)	83.3 (62.0–92.6)	66.2–100

⁎ Q: The Cochran's Q statistic is a measure assessing the existence of heterogeneity in pooled outcome measures, here proportions of HSV-2 virus isolation.

† I^2^: A measure assessing the magnitude of between-study variation that is due to true differences in proportions of HSV-2 virus isolation across studies rather than sampling variation.

‡ Prediction interval: A measure quantifying the distribution 95% interval of true proportions of HSV-2 virus isolation around the estimated pooled mean. Abbreviations: CI = Confidence interval, GUD = Genital ulcer disease, HSV-2 = Herpes simplex virus type 2.

In genital herpes cases (*n* = 11), the proportion of HSV-2 ranged between 53.0–100% with a median of 100% and a pooled mean proportion of 97.3% (95% CI: 84.4–100%) (Table 5). No study distinguished between primary and recurrent genital herpes.

**Fig. 2:**
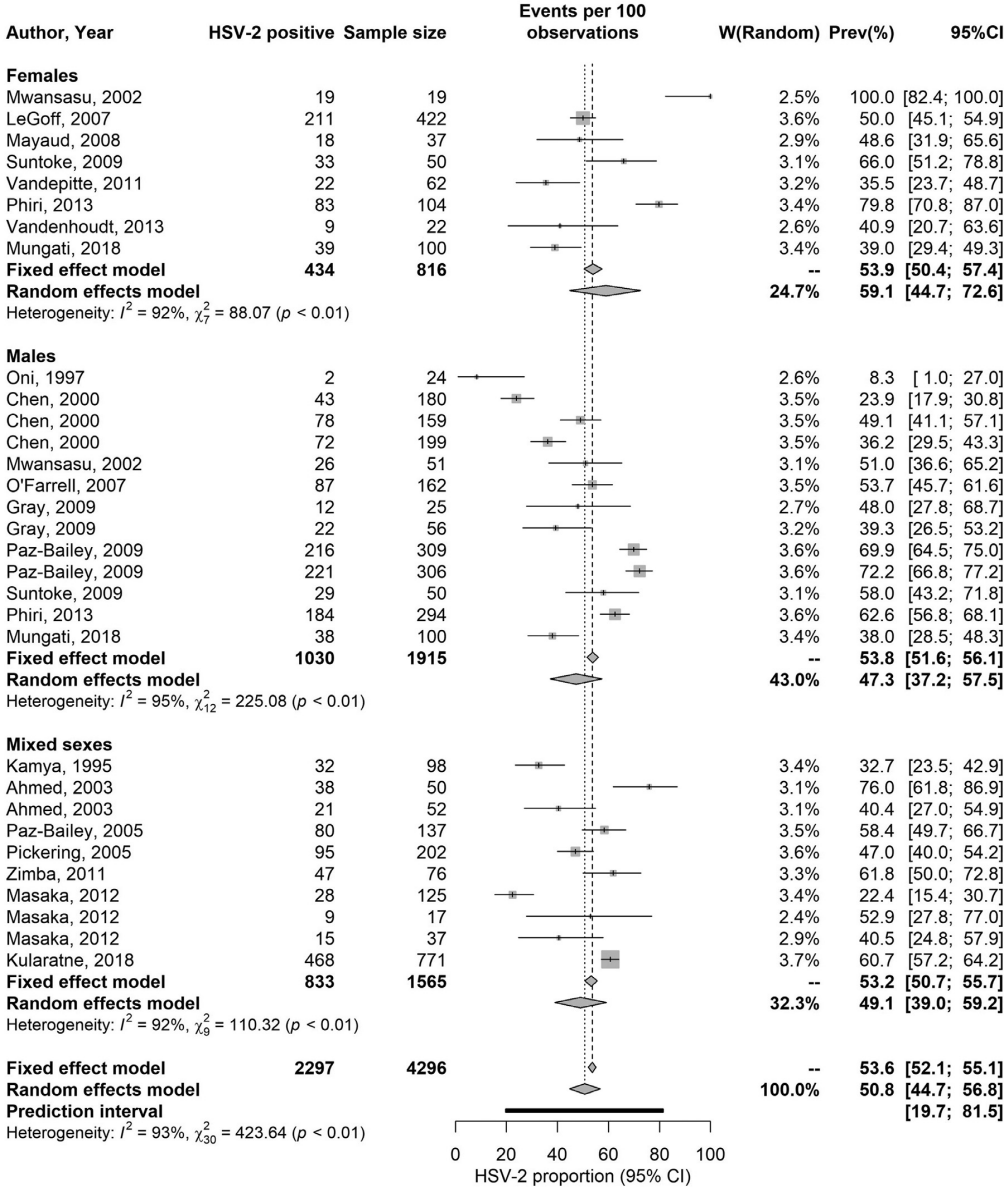

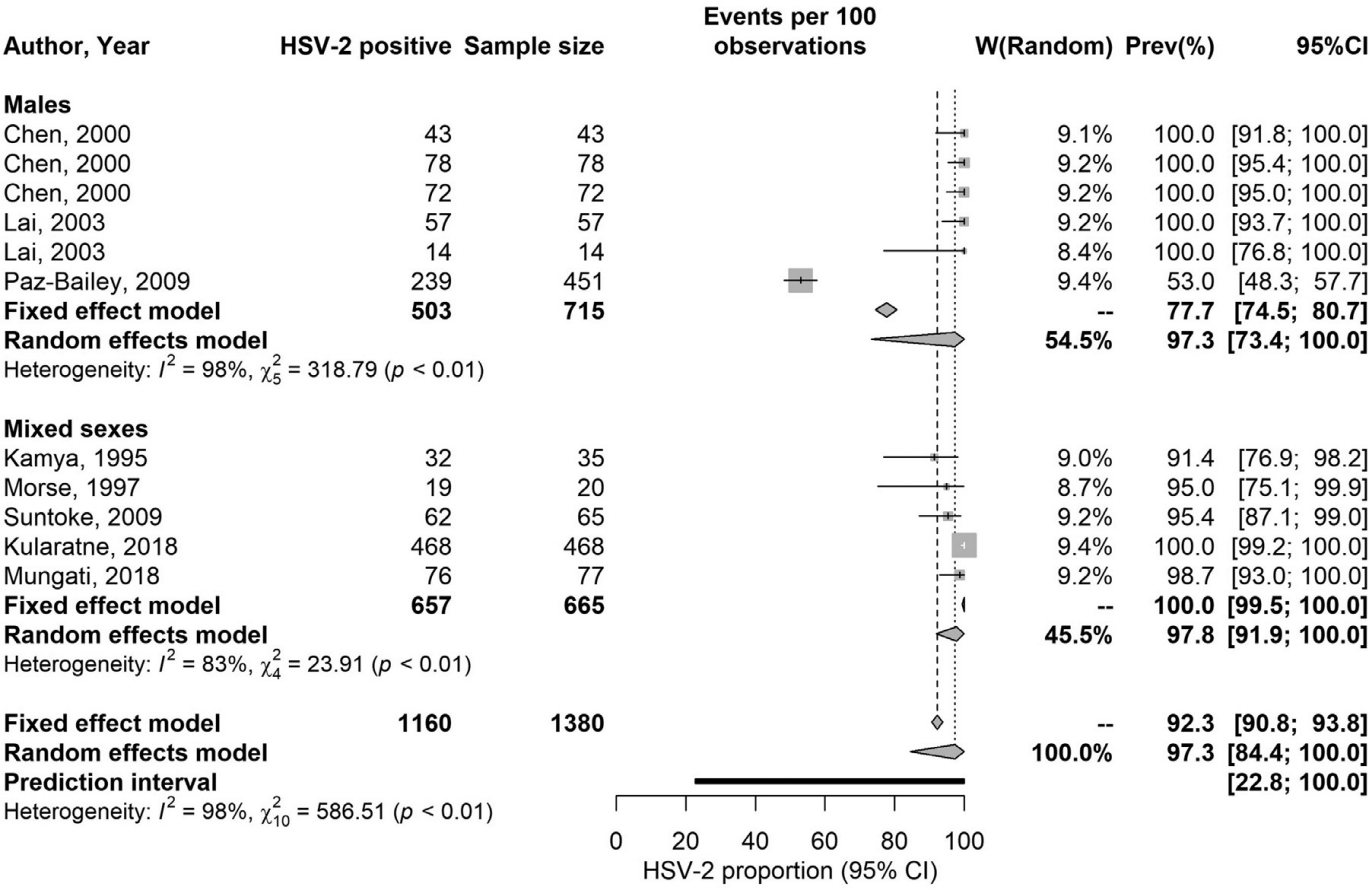
Forest plots presenting the outcomes of pooled mean proportions of HSV-2 virus isolation in clinically diagnosed genital ulcer disease (GUD) and in clinically diagnosed genital herpes in sub-Saharan Africa. Abbreviations: HSV-2 = Herpes simplex virus type 2.

These meta-analyses showed evidence of heterogeneity (p-value<0.001, I²>50%, and wide prediction intervals). Forest plots are in Fig. 2.

### 3.7 Quality assessment

Quality assessment of diagnostic methods excluded 51 publications due to potential problems in the diagnostic assays employed (Fig. 1). Quality assessment of included seroprevalence measures is summarized in Table S11. Briefly, 288 studies (89.4%) had high precision, 88 studies (27.3%) and 85 studies (26.4%) had low ROB in sampling methods and in the response rate domains, respectively. Twenty-three studies (7.1%) had high ROB in both quality domains.

## 4 Discussion

This systematic review presented a detailed assessment of HSV-2 epidemiology in sub-Saharan Africa. HSV-2 seroprevalence in sub-Saharan Africa, estimated at 37%, was much higher than that estimated in other global regions, from 8% in Europe to 18% in the Americas [1]. Strikingly, the results demonstrate that HSV-2 seroprevalence has been declining by about 2% per year over the last three decades (Table 4). This decline is consistent with observed declines in HIV epidemics in SSA during the same period [62]. Drivers of the decline in HIV prevalence have been subject to debate, with various mechanisms posited, including natural epidemic dynamics [63], increased HIV-associated mortality [64,65], impact of interventions [65], heterogeneity in host susceptibility to HIV infection [66,67], and reductions in sexual risk behavior [65,[68], [69], [70], [71]]. Considering that HSV-2 seroprevalence provides an *objective proxy biomarker* of population-level sexual risk behavior [19], [20], [21], [22], [23], [24], our finding of rapidly declining HSV-2 seroprevalence suggests that sexual risk behavior has been declining, and that this decline has reduced the transmission of both HIV and HSV-2 infections. With evidence of HSV-2 infection increasing risk of HIV acquisition and transmission [10], [11], [12], [13], declining HSV-2 incidence may have also contributed to the decline in HIV incidence in SSA.

Despite declining HSV-2 transmission in SSA, the incidence rate is still high (Table 1), and much higher than that found elsewhere in the world [1]. For instance, the incidence rate in the United States (<1 per 100 person-years) [72] is an order of magnitude lower than in SSA. The results further demonstrate that age has a profound effect in HSV-2 epidemiology—age alone accounted for 30% of the seroprevalence variation (Table 4). HSV-2 infection in SSA is typically acquired at a young age, not long after sexual debut, especially for women, manifested in rapidly increasing seroprevalence with age before plateauing at high levels by ages 40–50 (Tables 1,3,4, and S8).

Population risk classification is an important determinant in HSV-2 epidemiology, accounting for 13% of seroprevalence variation (Table 4). Both incidence rate and seroprevalence were much higher in specific at-risk populations, such as female sex workers (Tables 2 and 4), with both measures displaying the “classical hierarchy” of STI exposure by sexual risk behavior, seen with other STIs [73,74]. Despite its prominence amongst higher-risk populations, HSV-2 infection is also widely disseminated in SSA with high levels of infection even in the lower-risk general population, in which over 25% of men and nearly 50% of women are infected (Tables 2 and 4).

Although HSV-2 seroprevalence is high everywhere in SSA, there are still considerable variations by subregion. Infection levels were highest in Eastern Africa followed by Southern Africa, Central Africa, and lowest in Western Africa (Table 4). Incidentally, this pattern is also seen for HIV infection with Eastern Africa and Southern Africa being most affected and Western Africa least affected [75,76]. This further suggests a strong link between HSV-2 and HIV epidemiologies [24], reflecting a similar mode of transmission and hinting at a biological/epidemiological synergy [10], [11], [12], [13], [14]. The results further demonstrate that women are almost twice as likely to be infected as men (Table 4), reflecting a higher bio-anatomical susceptibility to the infection [77,78].

Another finding of this study is that HSV-2 infection causes half the GUD cases in SSA (Table 5), confirming that this infection is the main cause of this disease in this part of the world where nearly 60 million individuals are estimated to be affected with HSV-related GUD [79]. Although HSV-2 seroprevalence is declining (Table 4), it will likely remain the main cause of GUD in SSA, as other causes such as syphilis have also been declining [80,81]. HSV-2 infection (as opposed to HSV-1 infection) also accounted for nearly all cases of genital herpes (>97%; Table 5). This finding is presumably due to the nature of HSV-1, which is widely acquired in childhood in SSA by oral transmission [33], and distinguishes this region from other global regions where there is an increasing role for HSV-1 in genital herpes [33], [34], [35], [36], with some countries already observing HSV-1 as the cause of a large proportion of first-episode genital herpes cases [33], [34], [35], [36].

This study had limitations, principally the unavailability of data for 15 of 45 African countries. There were also less data for Central and Western Africa than for Eastern and Southern Africa, in addition to data for seroprevalence eclipsing those of GUD and genital herpes. Despite these limitations, a large volume of data was available to sufficiently power an array of analyses. Included studies exhibited heterogeneity (Tables 2, 3, and S8); however, more than half of this heterogeneity (57%) was subsequently explained through meta-regression (Table 4). Studies differed by assay type, sample size, sampling method, and response rate, yet none of these characteristics appeared to affect seroprevalence, with the exception of response rate, where studies with lower or unknown response rates had a higher seroprevalence (Table 4). Overall, these limitations should not pose a barrier to the critical interpretation of this study's results or findings.

In conclusion, HSV-2 seroprevalence is declining rapidly in SSA. Yet, HSV-2 incidence and seroprevalence remain at high levels, with over a third of the population being infected. Age and subregion in SSA play a critical role in HSV-2 epidemiology and explain much of the observed variation in seroprevalence. The geographical distribution of this infection was similar to that of HIV infection, and the declines in HSV-2 seroprevalence mirrored those for HIV prevalence. These findings suggest that reductions in sexual risk behavior following the massive expansion of the HIV epidemic in this continent have contributed to reductions in both HIV and HSV-2, and suggest that the biological/epidemiologic synergy may have been an influencing factor. HSV-2 infection was found to be the etiological cause of half the GUD cases in this region, and virtually all cases of genital herpes. These findings demonstrate the urgent need for both prophylactic and therapeutic HSV-2 vaccines to tackle the disease burden of this infection [82], and argue for further acceleration of ongoing efforts for vaccine development [26,27,83].

## Declaration of Competing Interest

MH, FAH, CJ, and LJA declare no competing interests. KL is currently funded by the World Health Organization and by GlaxoSmithKline (GSK) for a gonorrhea vaccine modeling project.

## Appendix Supplementary materials

Image, application 1
