# Supplementary material for: Epidemiology of herpes simplex virus type 2 in sub-Saharan Africa: Systematic review, meta-analyses, and meta-regressions
Source: eClinicalMedicine. 2021 May 7;35:100876. doi: 10.1016/j.eclinm.2021.100876 (PMC8129943; doi:10.1016/j.eclinm.2021.100876)
Supplement: Supplementary file 1 [file mmc1.docx]

**Web extra material**

**Epidemiology of herpes simplex virus type 2 in sub-Saharan Africa: systematic review, meta-analyses, and meta-regressions**

Manale Harfouche MPH,^a,b*^ Farah M. Abu-Hijleh MD,^c*^ Charlotte James PhD,^d^ Katharine J. Looker PhD,^d^ and Laith J. Abu-Raddad PhD^a,b,e^

*^a^ Infectious Disease Epidemiology Group, Weill Cornell Medicine-Qatar, Cornell University, Qatar Foundation - Education City, Doha, Qatar*

*^b^ World Health Organization Collaborating Centre for Disease Epidemiology Analytics on HIV/AIDS, Sexually Transmitted Infections, and Viral Hepatitis, Weill Cornell Medicine–Qatar, Cornell University, Qatar Foundation – Education City, Doha, Qatar*

^c^ Department of Public Health, College of Health Sciences, Academic Quality Affairs Office, QU Health, Qatar University, Doha, Qatar

*^d^ Population Health Sciences, Bristol Medical School, University of Bristol, Bristol, United Kingdom*

^e^ Department of Population Health Sciences, Weill Cornell Medicine, Cornell University, New York, New York, USA

Table of Contents

[**Table S1.** Data sources and search criteria for systematically reviewing HSV-2 epidemiology in sub-Saharan Africa. 3](#_Toc67562702)

[**Box S1*.*** List of the 45 countries included in our definition for sub-Saharan Africa by subregion.^1^ 4](#_Toc67562703)

[**Box S2.** List of variables extracted from the relevant publications meeting the inclusion criteria. 5](#_Toc67562704)

[**Box S3.** Definitions of population type classifications^*^. 6](#_Toc67562705)

[**Box S4.** Variables included in the univariable and multivariable meta-regression analyses. 7](#_Toc67562706)

[**Table S2.** Studies reporting HSV-2 seroconversion rate or incidence rate in sub-Saharan Africa. 8](#_Toc67562707)

[**Figure S1*.*** Forest plot presenting the outcome of the pooled mean herpes simplex virus type 2 (HSV-2) incidence rate by population type and by study design in sub-Saharan Africa. 10](#_Toc67562708)

[A) Population type 10](#_Toc67562709)

[B) Study design 11](#_Toc67562710)

[**Table S3.** Studies reporting HSV-2 seroprevalence in Eastern Africa. This table includes only overall and not stratified seroprevalence measures. 12](#_Toc67562711)

[**Table S4.** Studies reporting HSV-2 seroprevalence in Southern Africa. This table includes only overall and not stratified seroprevalence measures, 15](#_Toc67562712)

[**Table S5.** Studies reporting HSV-2 seroprevalence in Western Africa. This table includes only overall and not stratified seroprevalence measures. 18](#_Toc67562713)

[**Table S6.** Studies reporting HSV-2 seroprevalence in Central Africa. This table includes only overall and not stratified seroprevalence measures. 19](#_Toc67562714)

[**Table S7.** Studies reporting HSV-2 seroprevalence across several regions in sub-Saharan Africa. This table includes only overall and not stratified seroprevalence measures. 20](#_Toc67562715)

[**Table S8.** Pooled mean estimates for herpes simplex virus type 2 seroprevalence among general populations by sex stratification in sub-Saharan Africa. 21](#_Toc67562716)

[**Figure S2.** Forest plots presenting the outcomes of the pooled mean herpes simplex virus type 2 (HSV-2) seroprevalence among the different at risk populations across the sub-Saharan Africa subregions. 22](#_Toc67562717)

[A) Eastern Africa 22](#_Toc67562718)

[B) Southern Africa 26](#_Toc67562719)

[C) Western Africa 31](#_Toc67562720)

[D) Central Africa 33](#_Toc67562721)

[E) Mixed regions in sub-Saharan Africa 34](#_Toc67562722)

[**Table S9.** Univariable and multivariable meta-regression analyses for herpes simplex virus type 2 seroprevalence among the different at risk populations in sub-Saharan Africa using the year of data collection as a categorical variable or as a linear term (in replacement of year of publication). 35](#_Toc67562723)

[**Table S10.** Studies reporting proportions of HSV-2 virus isolation in clinically-diagnosed genital ulcer disease and in clinically-diagnosed genital herpes in sub-Saharan Africa. 36](#_Toc67562724)

[**Table S11.** Summary of the precision assessment and risk of bias assessment for the studies reporting HSV-2 seroprevalence in sub-Saharan Africa. 37](#_Toc67562725)

[**References** 38](#_Toc67562726)

# **Table S1.** Data sources and search criteria for systematically reviewing HSV-2 epidemiology in sub-Saharan Africa.

| **PubMed (last searched: August 23^rd^, 2020)** |
| --- |
| (Simplexvirus[MeSH] OR Herpes Simplex[MeSH] OR Herpes Genitalis[MeSH] OR Herpes Hominis[Text] OR HSV type-2[Text] OR HSV type 2[Text] OR HSV2[Text] OR HSV-2[Text] OR HSV [Text] OR Human herpes virus[Text] OR Herpes simplex virus type 2[Text] OR Herpes simplex virus type-2[Text] OR herpes simplex virus 2[Text] OR herpes simplex virus-2[Text] OR herpes simplex type 2[Text] OR herpes simplex type-2[Text] OR herpes simplex 2[Text] OR herpes simplex-2[Text] OR Herpesvirus type 2[Text] OR Herpesvirus type-2[Text] OR Herpesvirus 2[Text] OR Herpesvirus-2[Text] OR Herpes virus type 2[Text] OR Herpes virus type-[Text] OR Herpes virus [Text] OR Herpes virus-2[Text] OR genital herpes[Text] OR Herpes Genitalis[Text] OR Stomatitis Herpetic[Text] OR Herpes Labialis[Text]) AND (Africa South of the Sahara [MeSH] OR Comoros [MeSH] OR Ethiopia[MeSH] OR Madagascar[MeSH] OR Mauritius[MeSH] OR Sao Tome and Principe [MeSH] OR “Seychelles”[ MeSH] OR Angola*[Text] OR Benin*[Text] OR Botswan*[Text] OR Batswana[Text] OR Burkina fas*[Text] OR Burkina*[Text] OR Burundi*[Text] OR Cameroon*[Text] OR Cabo Verde*[Text] OR cape verd*[Text] OR Central Africa Republic[Text] Central Africa*[Text] OR Chad*[Text] OR Comor*[Text] OR Congo*[Text] OR Cote d’Ivoire[Text] OR Ivorian*[Text] OR Democratic Republic of Congo[Text] OR Equatorial Guinea*[Text] OR Equatoguinean*[Text] OR Eritr*[Text] OR Ethiop*[Text] OR Gabon*[Text] OR Gambia*[Text] OR Ghana*[Text] OR Ghinea*[Text] OR Guinea-Bissau[Text] OR Kenya*[Text] OR Lesotho*[Text] OR Basotho*[Text] OR Liberia*[Text] OR Madagascar*[Text] OR Malagasy*[Text] OR Malawi*[Text] OR Mali*[Text] OR Maurit*[Text] OR Mozambi*[Text] OR Namibia*[Text] OR Niger*[Text] OR Nigeria*[Text] OR Rwanda*[Text] OR Sao Tome and Principe*[Text] OR Sao Tome*[Text] OR Senegal*[Text] OR Seychell*[Text] OR Sierra Leone*[Text] OR South Africa*[Text] OR Swazi*[Text] OR Togo*[Text] OR Uganda*[Text] OR United Republic of Tanzania[Text] OR Tanzan*[Text] OR Zambia*[Text] OR Zimbabwe*[Text] OR Mauritania*[Text]) |
| **Embase (last searched: August 23^rd^, 2020)** |
| (exp Herpes simplex/ or exp Herpesviridae/) OR (Herpes simplex or Herpes simplex virus or HSV type-2 or HSV type 2 or HSV2 or HSV-2 or HSV 2 or human herpes virus or Herpes simplex virus type 2 or Herpes simplex virus type-2 or herpes simplex virus 2 or herpes simplex virus-2 or herpes simplex type 2 or herpes simplex type-2 or herpes simplex 2 or herpes simplex-2 or Herpesvirus type 2 or Herpesvirus type-2 or Herpesvirus 2 or Herpesvirus-2 or Herpes virus type 2 or Herpes virus type-2 or Herpes virus 2 or Herpes virus-2 or genital herpes or Herpes Genitalis or herpes labialis or herpetic stomatitis).mp.) AND (exp "Africa south of the Sahara" or exp Southern African/ or African/ or exp Central African/ or exp West African/ or exp South African/ or exp Central African Republic/ or exp East African/ or exp Mauritius/ or exp Mauritania/ or exp "Sao Tome and Principe"/ or exp Seychelles/) or (angola* or Benin* or Botswan* or Batswana* or Burkina Fas* or Burkina* or Burundi* or Cameroon* or Cabo verde* or Cape Verd* or Central Africa* or "Central African Republic*" or Chad* or Comor* or Congo* or Cote D'ivoire* or Ivorian* or "Democratic Republic of Congo*" or Equatorial Guinea* or Equatoguinean* or Ethiop* or Eritr* or Gabon* or Gambia* or Ghana* or Ghinea* or Guinea-Bissau* or Kenya* or Lesotho* or Basotho* or Liberia* or Madagascar* or Malagasy* or Malawi* or Mali* or Maurit* or Mozambi* or Namibia* or Niger* or Nigeria* or Rwanda* or "Sao Tome and Principe*" or Sao Tome* or Senegal* or Seychell* or Sierra Leone* or South Africa* or Swazi* or Togo* or Uganda* or United republic of Tanzania* or Tanza* or Zambia* or Zimbabwe* or Mauritania*).mp.) |

Abbreviations: HSV-2 = Herpes simplex virus type 2.

# **Box S1*.*** List of the 45 countries included in our definition for sub-Saharan Africa by subregion.^1^

**Eastern Africa**: Comoros, Ethiopia, Eritrea, Kenya, Madagascar, Mauritius, Rwanda, Seychelles, Tanzania, and Uganda.

**Southern Africa**: Angola, Botswana, Lesotho, Malawi, Mozambique, Namibia, South Africa, Eswatini (formerly known as Swaziland), Zambia, and Zimbabwe.

**Western Africa**: Benin, Burkina Faso, Cabo Verde, Cote d’Ivoire, Gambia, Ghana, Guinea-Bissau, Guinea, Liberia, Mali, Niger, Nigeria, Senegal, Sierra Leone, and Togo.

**Central Africa**: Burundi, Cameroon, Central African Republic, Chad, Congo, Democratic Republic of Congo, Equatorial Guinea, Gabon, and Sao Tome-and Principe.

**Northern Africa**: Mauritania.

# **Box S2.** List of variables extracted from the relevant publications meeting the inclusion criteria.

1. Author(s)
2. Publication title
3. Publication year
4. Year(s) of data collection
5. Country of origin
6. Country of survey
7. City
8. Study site
9. Study design
10. Study sampling procedure
11. Study population
12. Population demographic characteristics (e.g., sex and age)
13. Diagnostic assay
14. Sample size
15. HSV-2 outcome measures

Abbreviations: HSV-2 = Herpes simplex virus type 2

# **Box S3.** Definitions of population type classifications^*^.

| 1. **General populations** (populations at low risk): these include populations at lower risk of exposure to HSV-2, such as antenatal clinic attendees, blood donors, and pregnant women, among others. 2. **Intermediate-risk populations**: these include populations who presumably have frequent sexual contacts with populations engaging in high sexual risk behavior, and have therefore a higher risk of exposure to HSV-2 than the general population. These comprise prisoners, people who inject drugs, and truck drivers, among others. 3. **Higher-risk populations**: these include populations at high risk of exposure to HSV-2 as a consequence of specific sexual risk behaviors such as female sex workers, men who have sex with men, male sex workers, and transgender populations, among others. 4. **HIV-negative populations**: these include populations with confirmed HIV negative status as part of the study’s inclusion criteria. 5. **HIV-positive individuals and individuals in HIV discordant couples**: these include populations who are HIV positive or are in a spousal relationship with an HIV positive individual. 6. **STI clinic attendees and symptomatic populations**: these include patients attending STI clinics, or have clinical manifestations related to an STI. 7. **Other populations**^†^: these include populations not satisfying above definitions, or populations with an undetermined risk of acquiring HSV-2 infection. |
| --- |

^*^ These population types were selected based on our understanding of HIV/STI epidemiology and the variability of risk of exposure in different population types, as informed by existing literature on HIV and STI.^2-6^

^†^ Populations with uncertain risk of exposure to HSV-2 infection were classified as “Other populations”.

Abbreviations: HSV-2 Herpes simplex virus type 2, STI = Sexually transmitted infection, HIV = Human immunodeficiency virus.

# **Box S4.** Variables included in the univariable and multivariable meta-regression analyses.

| 1. Population type as defined in Box S3 2. Sex 3. Age groups classified to best fit reported data as:  - <20 years-old - 20-30 years-old - 30-40 years-old - 40-50 years-old - >50 years-old - Mixed age bands  1. African subregion as defined in Box S1 2. National income as classified by the World Bank^7^ 3. Assay type:  - Western Blot - ELISA - Rapid test  1. Sample size:  - ≥100 - <100  1. Sampling method;  - Probability-based sampling - Non-probability based sampling  1. Response rate:  - ≥80% - <80% - Unclear  1. Year of publication category^*^:  - ≤2005 - 2006-2015 - >2015  1. Year of publication as a linear term 2. Year of data collection category^†^  - <2000 - 2000-2010 - >2010  1. Year of data collection as a linear term |
| --- |

^*^ This categorization was informed by the distribution of studies by year of publication and by the interest in examining trends and changes in trends by a unit of one decade

^†^ The categories were set based on the observed median time between the year of publication and year of data collection of 5 years.

Abbreviations: ELISA = Enzyme-linked immunosorbent assay

# **Table S2.** Studies reporting HSV-2 seroconversion rate or incidence rate in sub-Saharan Africa.

| **Author, year** | **Year(s) of data collection** | **Country** | **Original study design** | **Population characteristics** | **HSV-2 serological assay** | **Sample size** | **Follow-up duration** | **Person-years of follow-up** | **HSV-2 seroconversion rate (%)** | **HSV-2 incidence rate (per 100 person-years)** |
| --- | --- | --- | --- | --- | --- | --- | --- | --- | --- | --- |
| **General populations** | | |  |  |  |  |  |  |  |  |
| Abdool Karim, 2015^8^ | 2007-10 | South Africa | RCT | Women enrolled in a clinical trial | ELISA | 422 | 18 months | 561.3 | 20.6 | 15.5 |
| Akinyi, 2017^9^ | 2007-10 | Kenya | Cohort | Participants in the “Incidence Cohort” study | ELISA | 673 | 1 year | - | - | 7.3 |
| Biraro, 2013^10^ | 1990-94 | Uganda | Cohort | Male samples collected between 1990-1994 | ELISA | - | 18 months | 3,404.0 | - | 2.3 |
| Biraro, 2013^10^ | 1995-99 | Uganda | Cohort | Male samples collected between 1995-1999 | ELISA | - | 18 months | 4,662.0 | - | 2.0 |
| Biraro, 2013^10^ | 2000-04 | Uganda | Cohort | Male samples collected between 2000-2004 | ELISA | - | 18 months | 4,933.0 | - | 2.2 |
| Biraro, 2013^10^ | 2005-07 | Uganda | Cohort | Male samples collected between 2005-2007 | ELISA | - | 18 months | 2,107.0 | - | 2.8 |
| Biraro, 2013^10^ | 1990-94 | Uganda | Cohort | Female samples collected between 1990-1994 | ELISA | - | 18 months | 2,420.0 | - | 4.3 |
| Biraro, 2013^10^ | 1995-99 | Uganda | Cohort | Female samples collected between 1995-1999 | ELISA | - | 18 months | 3,385.0 | - | 3.9 |
| Biraro, 2013^10^ | 2000-04 | Uganda | Cohort | Female samples collected between 2000-2004 | ELISA | - | 18 months | 4,103.0 | - | 3.6 |
| Biraro, 2013^10^ | 2005-07 | Uganda | Cohort | Female samples collected between 2005-2007 | ELISA | - | 18 months | 1,878.0 | - | 3.7 |
| De Baetselier, 2015^11^ | 2009-10 | South Africa | Cohort | Females enrolled in an RCT | ELISA | 407 | 56 weeks | 229.5 | 13.4 | 18.3 |
| del Mar Pujades, 2002^12^ | 1991-94 | Tanzania | RCT | Males enrolled in an RCT | ELISA | 221 | 2 years | - | 11.3 | - |
| del Mar Pujades, 2002^12^ | 1991-94 | Tanzania | RCT | Females enrolled in an RCT | ELISA | 206 | 2 years | - | 17.5 | - |
| Hallfors, 2017^13^ | 2011-14 | Kenya | RCT | Orphans in grades 7 and 8 | ELISA | 357 | 3 years | - | 30.8 | - |
| Heffron, 2011^14^ | 2006-07 | Zambia | Cohort | Non-migrant male farmers | ELISA | 484 | 16 months | - | 6.8 | - |
| Jewkes, 2008^15^ | 2003-04 | South Africa | RCT | Individuals in the intervention arm | ELISA | - | 2 years | 1,759.2 | - | 3.2 |
| Jewkes, 2008^15^ | 2003-04 | South Africa | RCT | Individuals in the control arm | ELISA | - | 3 years | 1,623.3 | - | 4.6 |
| Kamali, 1999^16^ | 1990-93 | Uganda | Cohort | 15-24 years old males and females | WB | 373 | 4 years | - | 20.9 | - |
| Kamali, 2003^17^ | 1994-00 | Uganda | RCT | Individuals in intervention arm A | EIA | - | 6 years | 4,381.6 | - | 2.3 |
| Kamali, 2003^17^ | 1994-00 | Uganda | RCT | Individuals in intervention arm B | EIA | - | 6 years | 4,595.5 | - | 3.6 |
| Kamali, 2003^17^ | 1994-00 | Uganda | RCT | Individuals in control arm C | EIA | - | 6 years | 4,628.6 | - | 3.5 |
| Kebede, 2004^18^ | 1997-02 | Ethiopia | Cohort | HSV-2 seronegative males and females | ELISA | 953 | 5 years | 3,225.5 | 58.0 | 1.8 |
| McFarland, 1999^19^ | 1993-97 | Zimbabwe | Cohort | Male factory workers | WB | 1,444 | 4 years | 3,316.0 | 14.1 | 6.2 |
| Mensche, 2020^20^ | 2007-13 | Malawi | Cohort | School students | ELISA | 2,072 | 6 years | - | 8.5 | - |
| Munjoma, 2010^21^ | 2002-04 | Zimbabwe | Cohort | Pregnant women | ELISA | 173 | 10 months | 144.2 | 11.9 | 13.9 |
| Nakubulwa, 2016^22^ | 2013-14 | Uganda | Cohort | Pregnant women | ELISA | 191 | - | - | 7.8 | - |
| Pettifor, 2016^23^ | 2011-12 | South Africa | RCT | Individuals in the control arm | ELISA | 1,114 | 3 years | 2,525.0 | 9.1 | 4.0 |
| Pettifor, 2016^23^ | 2011-12 | South Africa | RCT | Individuals in the intervention arm | ELISA | 1,214 | 3 years | 2,675.0 | 8.8 | 4.0 |
| Radebe, 2011^24^ | 2008-10 | South Africa | RCT | Male students | ELISA | - | 54 months | - | - | 2.9 |
| Radebe, 2011^24^ | 2008-10 | South Africa | RCT | Female students | ELISA | - | 54 months | - | - | 6.4 |
| Rosenberg, 2018^25^ | 2013-15 | South Africa | Cohort | 18-25 years old women | ELISA | 645 | 2 years | - | 6.8 | - |
| Sobngwi, 2009^26^ | 2002-04 | South Africa | RCT | Individuals in the control arm | ELISA | - | 21 months | 1,003.0 | - | 3.5 |
| Sobngwi, 2009^26^ | 2002-04 | South Africa | RCT | Individuals in the intervention arm | ELISA | - | 21 months | 995.0 | - | 2.3 |
| Stoner, 2018^27^ | 2011-15 | South Africa | Cohort | <20 years old young women | ELISA | 1,963 | 4 years | - | 5.9 | - |
| Tobian, 2009^28^ | 2002-06 | Uganda | RCT | 15-49 years old men | ELISA | - | 2 years | 5,793.7 | - | 4.9 |
| Tobian, 2012^29^ | 2002-07 | Uganda | RCT | Spouses of circumcised men | WB | 359 | 3 years | 656.5 | 11.1 | 6.1 |
| Tobian, 2012^29^ | 2002-07 | Uganda | RCT | Spouses of uncircumcised men | WB | 363 | 4 years | 648.5 | 11.3 | 6.3 |
| van de Wijgert, 2009^30^ | 1999-04 | Zimbabwe | Cohort | Zimbabwean women | ELISA | - | 5 years | - | - | 8.6 |
| Wagner, 1994^31^ | - | Uganda | Cohort | Seronegative adults | WB | 36 | 1 year | - | 16.7 | - |
| **Intermediate-risk populations** | | | |  |  |  |  |  |  |  |
| Kapiga, 2013^32^ | 2008-10 | Tanzania | Cohort | Women working in food facilities and bars | ELISA | 450 | 1 year | 339.0 | 21.5 | 28.6 |
| Meque, 2014^33^ | 2009-12 | Mozambique | Cohort | Women working in service facilities | ELISA | 151 | 1 year | - | 20.5 | - |
| Ondondo, 2014^34^ | 2005-06 | Kenya | Cohort | Fishermen | ELISA | - | 1 year | - | 23.6 | - |
| Riedner, 2006^35^ | 2000-02 | Tanzania | Cohort | Women working in a bar | EIA | - | 27 months | 98.0 | - | 17.3 |
| Tassiopoulos, 2007^36^ | 2002-03 | Tanzania | Cohort | Individuals working in a hotel and bar | ELISA | 360 | 1 year | 337.8 | 13.3 | 14.2 |
| **Higher-risk populations** | | |  |  |  |  |  |  |  |  |
| Braunstein, 2011b^37^ | 2007-09 | Rwanda | Cohort | FSWs in Rwanda | ELISA | 182 | 2 years | 150.0 | 1.9 | 8.7 |
| Chohan, 2009^38^ | 1993-06 | Kenya | Cohort | FSWs in Kenya | ELISA | 297 | 28 months | 499.0 | 38.7 | 23.0 |
| Kaul, 2007^39^ | 1998-02 | Kenya | RCT | FSWs in Kenya | ELISA | 121 | 4.4 years | - | 22.3 | - |
| Masese, 2014^40^ | 1993-11 | Kenya | Cohort | FSWs in Kenya | ELISA | 406 | 5 years | 809.0 | 40.4 | 21.0 |
| Ramjee, 2005^41^ | 1996-97 | South Africa | RCT | FSWs in South Africa | ELISA | 44 | 3.6 years | - | 54.9 | - |
| Traore, 2013^42^ | 2009-11 | Burkina Faso | RCT | FSWs in Burkina Faso | ELISA | - | 1 year | - | - | 11.0 |
| **HIV-negative populations** | | |  |  |  |  |  |  |  |  |
| De Bruyn, 2011^43^ | 2002-05 | South Africa | RCT | Women in Durban | ELISA | 495 | 2 years | 689.0 | 15.4 | 11.0 |
| De Bruyn, 2011^43^ | 2002-05 | South Africa | RCT | Women in Johannesburg | ELISA | 328 | 3 years | 443.0 | 10.4 | 7.7 |
| De Bruyn, 2011^43^ | 2002-05 | Zimbabwe | RCT | Women in Harare | ELISA | 1,193 | 4 years | 949.0 | 8.4 | 5.2 |
| McCormack, 2010^44^ | 2005-08 | 4 African countries^*^ | RCT | Females in the intervention arm receiving 2% gel | ELISA | 297 | 1 year | - | 11.5 | - |
| McCormack, 2010^44^ | 2005-09 | 4 African countries^*^ | RCT | Females in the intervention arm receiving 0.5% gel | ELISA | 919 | 1 year | - | 11.9 | - |
| McCormack, 2010^44^ | 2005-09 | 4 African countries^*^ | RCT | Females in the control arm | ELISA | 888 | 1 year | - | 13.0 | - |
| Mehta, 2012^45^ | 2002-05 | Kenya | RCT | Circumcised men | ELISA | 986 | 2 years | 1,493.5 | 8.7 | 5.8 |
| Mehta, 2012^45^ | 2002-05 | Kenya | RCT | Uncircumcised men | ELISA | 1,035 | 2 years | 1,628.5 | 9.7 | 6.1 |
| Mehta, 2013^46^ | 2002-07 | Kenya | RCT | Men enrolled in an RCT | ELISA | 2,044 | 6 years | - | 33.5 | - |
| Mlisana, 2012^47^ | 2004-05 | South Africa | Cohort | HIV Negative women | ELISA | - | 2 years | - | - | 26.0 |
| Perti, 2014^48^ | - | South Africa | Cohort | HIV negative pregnant women | WB | 91 | 6 weeks | - | 0.0 | - |
| **HIV-positive individuals and individuals in HIV discordant couples** | | | | | | | | | | |
| Celum, 2014^49^ | 2008-11 | Kenya and Uganda | RCT | Partners of HIV positive persons in an RCT | WB | 1,041 | 2 years | 1,422.2 | 7.6 | 5.6 |
| Celum, 2014^49^ | 2008-11 | Kenya and Uganda | RCT | Partners of HIV positive persons in an RCT | WB | 481 | 2 years | 672.0 | 10.8 | 7.7 |
| Cowan, 2008a^50^ | 1997-00 | Zimbabwe | RCT | HIV positive mothers with HIV positive infants | WB | 50 | 6 weeks | - | 22.0 | - |
| Cowan, 2008a^50^ | 1997-00 | Zimbabwe | RCT | HIV positive mothers with HIV negative infants | WB | 106 | 6 weeks | - | 15.1 | - |
| del Mar Pujades, 2002^12^ | 1991-94 | Tanzania | RCT | HIV positive females enrolled in a large RCT | ELISA | 32 | 2 years | - | 38.7 | - |
| del Mar Pujades, 2002^12^ | 1991-94 | Tanzania | RCT | HIV positive Males enrolled in a large RCT | ELISA | 22 | 2 years | - | 31.8 | - |
| Muiru, 2013^51^ | 2007-09 | Kenya | Cohort | Participants from HIV discordant couples | ELISA | 382 | 2 years | 512.6 | 19.8 | 14.8 |
| **Other populations** | |  |  |  |  |  |  |  |  |  |
| Okuku, 2011^52^ | 2005-08 | Kenya | Cohort | Women from different risk populations | ELISA | 164 | 4 years | - | 23.8 | 22.1 |
| Okuku, 2011^52^ | 2005-08 | Kenya | Cohort | Men from different risk populations | ELISA | 443 | 4 years | - | 11.9 | 9.0 |
| van de Wijgert, 2009^30^ | 1999-04 | Uganda | Cohort | Women from different risk populations | ELISA | - | 4 years | - | - | 10.6 |

^*^ The four African countries were: South Africa, Tanzania, Uganda, and Zambia.

Abbreviations: EIA = Enzyme immunosorbent assay, ELISA = Enzyme-linked immunosorbent assay, FSWs = Female sex workers, HIV = Human immunodeficiency virus, HSV-2 = Herpes simplex virus type 2, RCT = Randomized controlled trial, WB = Western blot.

# **Figure S1*.*** Forest plot presenting the outcome of the pooled mean herpes simplex virus type 2 (HSV-2) incidence rate by population type and by study design in sub-Saharan Africa.

##
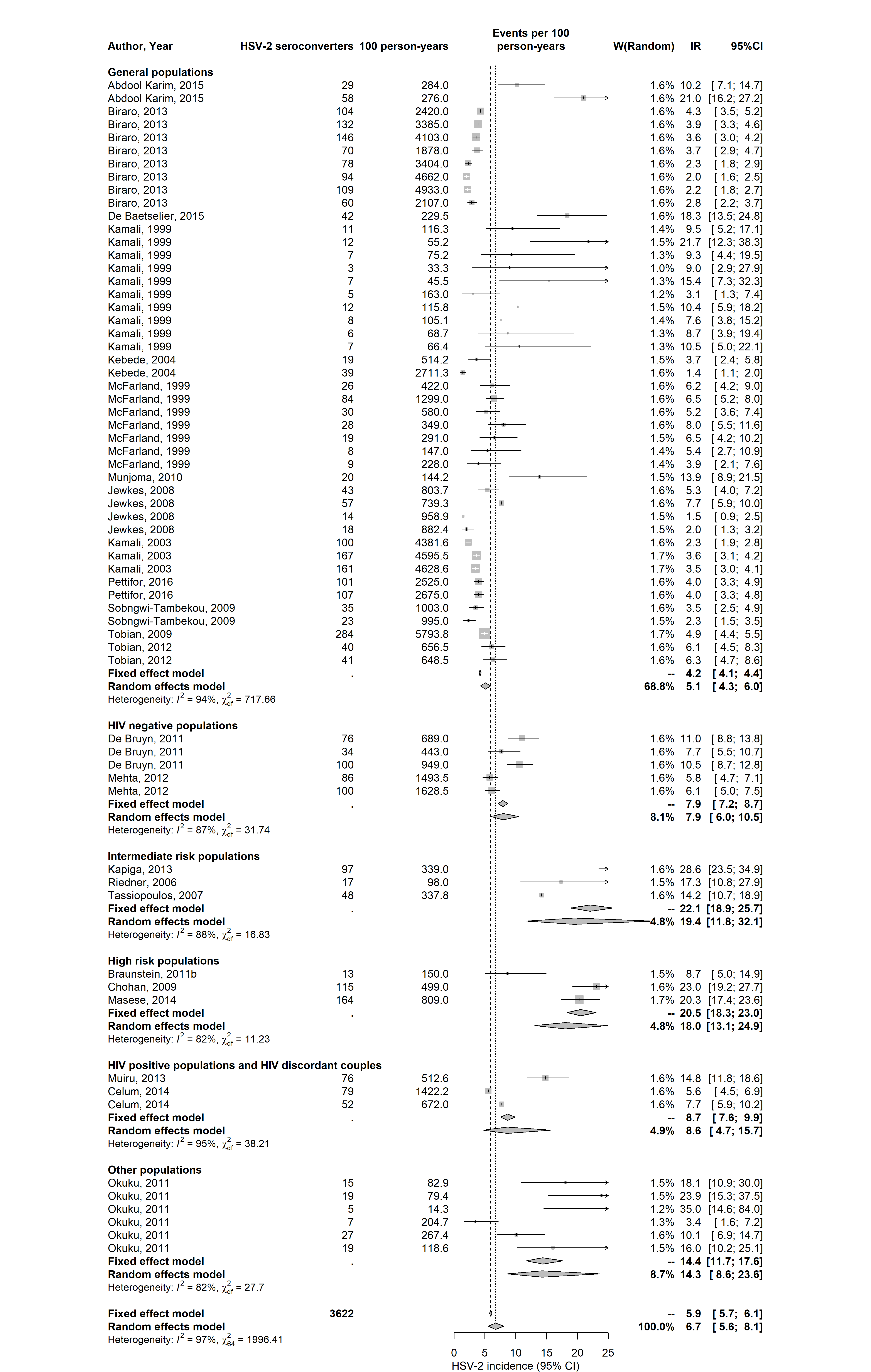
Population type

Abbreviations: HSV-2 = Herpes simplex virus type 2.

##
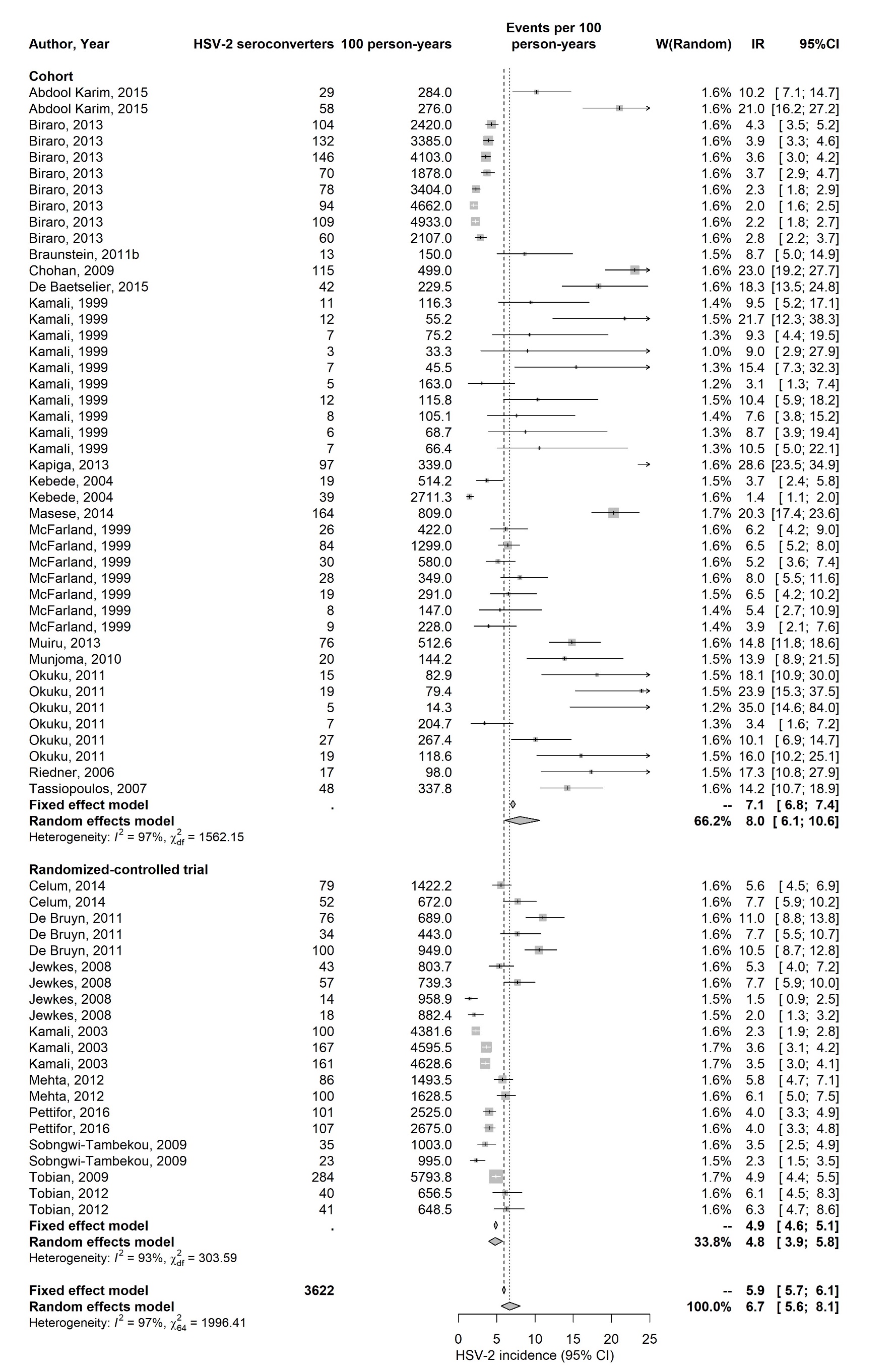
Study design

Abbreviations: HSV-2 = Herpes simplex virus type 2.

# **Table S3.** Studies reporting HSV-2 seroprevalence in Eastern Africa. This table includes only overall and not stratified seroprevalence measures.

| **Author, year** | **Year(s) of data collection** | **Country** | **Study site** | **Original study design^*^** | **Sampling method** | **Population** | **HSV-2 serological assay** | **Sample size** | **HSV-2 seroprevalence (%)** |
| --- | --- | --- | --- | --- | --- | --- | --- | --- | --- |
| **General populations** | | |  |  |  |  |  |  |  |
| Akinyi, 2017^9^ | 2007-10 | Kenya | Community | Cohort | Conv | 16-17 years old adolescents | ELISA | 243 | 10.7 |
| Amornkul, 2009^53^ | 2003-04 | Kenya | Community | CS | RS | <34 years old women | ELISA | 930 | 53.0 |
| Amornkul, 2009^53^ | 2003-04 | Kenya | Community | CS | RS | <34 years old men | ELISA | 832 | 25.8 |
| Anjulo, 2016^54^ | 2013-14 | Ethiopia | Outpatient clinic | CS | RS | Antenatal clinic attendees | ELISA | 252 | 32.1 |
| Behling, 2015^55^ | 2003-06 | Kenya | Community | RCT | RS | School students in a large trial | ELISA | 139 | 14.4 |
| Biraro, 2013^10^ | 1990-94 | Uganda | Community | CS | Conv | Male samples collected between 1990-1994 | ELISA | 1,083 | 41.0 |
| Biraro, 2013^10^ | 1995-99 | Uganda | Community | CS | Conv | Male samples collected between 1995-1999 | ELISA | 1,067 | 38.9 |
| Biraro, 2013^10^ | 2000-04 | Uganda | Community | CS | Conv | Male samples collected between 2000-2004 | ELISA | 1,485 | 28.5 |
| Biraro, 2013^10^ | 2005-07 | Uganda | Community | CS | Conv | Male samples collected between 2005-2007 | ELISA | 1,539 | 30.8 |
| Biraro, 2013^10^ | 1990-94 | Uganda | Community | CS | Conv | Female samples collected between 1990-1994 | ELISA | 1,118 | 62.4 |
| Biraro, 2013^10^ | 1995-99 | Uganda | Community | CS | Conv | Female samples collected between 1995-1999 | ELISA | 1,303 | 55.3 |
| Biraro, 2013^10^ | 2000-04 | Uganda | Community | CS | Conv | Female samples collected between 2000-2004 | ELISA | 1,799 | 48.0 |
| Biraro, 2013^10^ | 2005-07 | Uganda | Community | CS | Conv | Female samples collected between 2005-2007 | ELISA | 2,060 | 50.5 |
| Braunstein, 2011a^56^ | 2006-07 | Rwanda | Community | CS | Conv | VCT clients | ELISA | 1,250 | 43.2 |
| De Walque, 2012^57^ | 2009-10 | Tanzania | Community | RCT | RS | Controls in a RCT | ELISA | 1,124 | 33.9 |
| De Walque, 2012^57^ | 2009-10 | Tanzania | Community | RCT | RS | Intervention group A | ELISA | 615 | 36.8 |
| De Walque, 2012^57^ | 2009-10 | Tanzania | Community | RCT | RS | Intervention group B | ELISA | 660 | 34.2 |
| Dhont, 2010^58^ | 2007-09 | Rwanda | Outpatient clinic | CS | Conv | Fertile women | ELISA | 281 | 41.0 |
| Dhont, 2010^58^ | 2007-09 | Rwanda | Outpatient clinic | CS | Conv | Infertile women | ELISA | 304 | 59.0 |
| Dhont, 2010^58^ | 2007-09 | Rwanda | Outpatient clinic | CS | Conv | Male partners of fertile women | ELISA | 170 | 35.0 |
| Dhont, 2010^58^ | 2007-09 | Rwanda | Outpatient clinic | CS | Conv | Male partners of infertile women | ELISA | 251 | 51.0 |
| Doyle, 2010^59^ | 2007-08 | Tanzania | Community | CS | Conv | Females in the control arm of an RCT | ELISA | 3,238 | 42.5 |
| Doyle, 2010^59^ | 2007-08 | Tanzania | Community | CS | Conv | Males in the control arm of an RCT | ELISA | 3,493 | 26.7 |
| Duflo, 2015^60^ | 2009-10 | Kenya | Community | Cohort | RS | <21 years old women | ELISA | 5,509 | 11.8 |
| Duflo, 2015^60^ | 2009-10 | Kenya | Community | Cohort | RS | <21 years old men | ELISA | 6,302 | 7.4 |
| Ghebrekidan, 1999^61^ | 1995 | Eritrea | Hospital | CS | Conv | Rashaida tribe members | WB | 45 | 5.0 |
| Ghebrekidan, 1999^61^ | 1995 | Eritrea | Outpatient clinic | CS | Conv | Pregnant women | WB | 102 | 23.0 |
| Ghebrekidan, 1999^61^ | 1995 | Eritrea | Community | CS | Conv | 1-5 years old children | WB | 54 | 11.0 |
| Ghebrekidan, 1999^61^ | 1995 | Eritrea | Community | CS | Conv | >5 years old children | WB | 70 | 1.0 |
| Ghebremichael, 2011^62^ | 2002-03 | Tanzania | Community | CS | Conv | >20 years old men | EIA | 567 | 39.2 |
| Ghebremichael, 2012^63^ | 2002-03 | Tanzania | Community | CS | RS | >20 years old women | EIA | 1,418 | 42.9 |
| Gorander, 2006^64^ | - | Tanzania | Hospital | CS | Conv | Blood donors | WB | 196 | 41.3 |
| Guwatudde, 2009^65^ | 2006 | Uganda | Community | Cohort | Conv | 15-49 years old males and females | ELISA | 2,025 | 57.0 |
| Hallfors, 2015^66^ | 2011-12 | Kenya | Community | RCT | CRS | Students | ELISA | 837 | 3.3 |
| Hokororo, 2015^67^ | 2012 | Tanzania | Outpatient clinic | CS | Conv | Pregnant women | ELISA | 403 | 34.7 |
| Holt, 2003^68^ | 1992 | Ethiopia | Outpatient clinic | CS | Conv | Antenatal clinic attendees | WB | 85 | 26.0 |
| Jespers, 2014^69^ | 2010-11 | Kenya | Outpatient clinic | CS | Conv | Kenyan women | ELISA | 110 | 28.0 |
| Jespers, 2014^69^ | 2010-11 | Kenya | Outpatient clinic | CS | Conv | Kenyan pregnant women | ELISA | 30 | 17.0 |
| Jespers, 2014^69^ | 2010-11 | Kenya | Outpatient clinic | CS | Conv | Adolescents | ELISA | 30 | 37.0 |
| Kamali, 1999^16^ | 1990-93 | Uganda | Community | CS | Conv | 15-54 years old males | WB | 367 | 36.0 |
| Kamali, 1999^16^ | 1990-93 | Uganda | Community | CS | Conv | 15-54 years old females | WB | 541 | 71.5 |
| Kamali, 2002^70^ | 1994-00 | Uganda | Community | RCT | Conv | Participants in trial Arm A - information only | EIA | 2,396 | 28.4 |
| Kamali, 2002^70^ | 1994-00 | Uganda | Community | RCT | Conv | Participants in trial Arm B - STI awareness | EIA | 2,417 | 27.9 |
| Kamali, 2002^70^ | 1994-00 | Uganda | Community | RCT | Conv | Participants in trail Arm C - routine health services | EIA | 2,262 | 28.1 |
| Kapiga, 2006^71^ | 2002-03 | Tanzania | Community | CS | CRS | 20-44 years old women | EIA | 1,418 | 43.8 |
| Kapiga, 2006^71^ | 2002-03 | Tanzania | Community | CS | CRS | 20-44 years old men | EIA | 566 | 39.1 |
| Kasubi, 2006^72^ | - | Tanzania | Outpatient clinic | CS | Conv | <15 years old children | WB | 565 | 27.3 |
| Kebede, 2004^18^ | 1997-02 | Ethiopia | Community | Cohort | Conv | >19 years old adults | ELISA | 1,612 | 40.9 |
| Kuteesa, 2020^73^ | 2017-18 | Uganda | Community | CS | RS | 15-24 years old adults | ELISA | 1,270 | 32.0 |
| Mehta, 2018^74^ | 2014-16 | Kenya | Community | Cohort | Conv | Healthy women | ELISA | 252 | 56.8 |
| Mehta, 2018^74^ | 2014-16 | Kenya | Community | Cohort | Conv | Healthy men | ELISA | 252 | 46.6 |
| MOH Uganda, 2006^75^ | 2004-05 | Uganda | Community | CS | CRS | General population | ELISA | 17,953 | 46.1 |
| Msuya, 2002^76^ | 1999 | Tanzania | Outpatient clinic | CS | RS | Women attending antenatal clinics | ELISA | 382 | 39.0 |
| Msuya, 2007^77^ | 1999 | Tanzania | Outpatient clinic | CS | Conv | 1999 survey of pregnant women | ELISA | 382 | 39.0 |
| Msuya, 2009^78^ | 2002-04 | Tanzania | Outpatient clinic | CS | Conv | 2002-04 survey of pregnant women | ELISA | 1,271 | 33.6 |
| Nakku-Joloba, 2014^79^ | 2004 | Uganda | Community | CS | RS | Healthy adults | ELISA | 1,124 | 58.0 |
| Nakubulwa, 2015^80^ | 2013 | Uganda | Hospital | CC | Conv | Pregnant women with PROM | ELISA | 87 | 56.0 |
| Nakubulwa, 2015^80^ | 2013 | Uganda | Hospital | CC | Conv | Pregnant women in labor | ELISA | 87 | 53.0 |
| Nakubulwa, 2016^22^ | 2013-14 | Uganda | Hospital | Cohort | Conv | Pregnant women | ELISA | 524 | 52.9 |
| NASCOP, 2009^81^ | 2007 | Kenya | Community | CS | CRS | Kenyan population | ELISA | 15,707 | 35.1 |
| Nilsen, 2005^82^ | - | Tanzania | Outpatient clinic | CS | Conv | Pregnant women | ELISA | 98 | 34.3 |
| Nilsen, 2005^82^ | - | Tanzania | Outpatient clinic | CS | Conv | Blood donors | ELISA | 81 | 34.6 |
| Norris, 2009^83^ | 2004 | Tanzania | Community | CS | RS | Male plantation residents | ELISA | 232 | 45.0 |
| Norris, 2009^83^ | 2004 | Tanzania | Community | CS | RS | Female plantation residents | ELISA | 218 | 68.0 |
| Nyiro, 2011^84^ | 2004 | Kenya | Community | CS | RS | Women participating in a DSS | ELISA | 563 | 32.0 |
| Nyiro, 2011^84^ | 2004 | Kenya | Outpatient clinic | CS | Conv | Women attending a VCT | ELISA | 263 | 44.0 |
| Oliver, 2018^85^ | 2014 | Kenya | Community | RCT | Conv | Women using contraceptives | ELISA | 457 | 55.6 |
| Otieno, 2015^86^ | 2007-09 | Kenya | Community | Cohort | Conv | >18 years old men | ELISA | 422 | 13.3 |
| Otieno, 2015^86^ | 2007-09 | Kenya | Community | Cohort | Conv | >18 years old women | ELISA | 424 | 44.8 |
| Reynolds, 2012^87^ | 2007-08 | Uganda | Outpatient clinic | RCT | Conv | >18 years adults in an RCT | ELISA | 1,404 | 88.0 |
| Sivapalasingam, 2014^88^ | 2010-11 | Kenya | Outpatient clinic | CC | Conv | Women reporting intravaginal practices | ELISA | 58 | 48.0 |
| Sivapalasingam, 2014^88^ | 2010-11 | Kenya | Outpatient clinic | CC | Conv | Women reporting no intravaginal practices | ELISA | 42 | 43.0 |
| Tedla, 2011^89^ | 2002-09 | Ethiopia | Community | CC | Conv | Patients with schizophrenia | ELISA | 216 | 7.9 |
| Tedla, 2011^89^ | 2002-09 | Ethiopia | Community | CC | Conv | Patients with bipolar disorder | ELISA | 199 | 15.4 |
| Tedla, 2011^89^ | 2002-09 | Ethiopia | Community | CC | RS | Healthy controls | ELISA | 80 | 10.0 |
| Tobian, 2009^28^ | 2002-06 | Uganda | Community | RCT | RS | 15-49 years old men | ELISA | 6,396 | 33.8 |
| Tobian, 2012^29^ | 2002-07 | Uganda | Community | RCT | Conv | Spouses of circumcised men | ELISA/WB | 835 | 55.9 |
| Tobian, 2012^29^ | 2002-07 | Uganda | Community | RCT | Conv | Spouses of uncircumcised men | ELISA/WB | 803 | 53.7 |
| Todd, 2006^90^ | 1994-95 | Tanzania | Community | CC | RS | Healthy women | ELISA | 430 | 63.5 |
| Todd, 2006^90^ | 1994-95 | Tanzania | Community | CC | RS | Healthy men | ELISA | 420 | 46.0 |
| Wagner, 1994^31^ | - | Uganda | Community | CS | Conv | >15 years old residents | WB | 212 | 67.9 |
| Wagner, 1994^31^ | - | Uganda | Community | CS | Conv | <15 years old children | WB | 45 | 2.2 |
| Weiss, 2001^91^ | 1997-98 | Kenya | Community | CS | CRS | Women from Kisumu | ELISA | 824 | 68.0 |
| Weiss, 2001^91^ | 1997-98 | Kenya | Community | CS | CRS | Men from Kisumu | ELISA | 583 | 35.0 |
| Winston, 2015^92^ | 2011-12 | Kenya | Community | CS | Conv | <21 years old street youth | ELISA | 175 | 19.0 |
| Yahya-Malima, 2008^93^ | 2003-04 | Tanzania | Outpatient clinic | CS | Conv | Pregnant women | ELISA | 1,296 | 20.7 |
| Yegorov, 2018^94^ | 2015-16 | Uganda | Outpatient clinic | CS | Conv | 18-45 years old women | ELISA | 58 | 58.6 |
| **Intermediate-risk populations** | | | | | | | |  |  |
| Ghebrekidan, 1999^61^ | 1995 | Eritrea | Community | CS | Conv | Guerrilla fighters | WB | 73 | 45.0 |
| Ghebrekidan, 1999^61^ | 1995 | Eritrea | Community | CS | Conv | Truck drivers | WB | 53 | 43.0 |
| Ghebrekidan, 1999^61^ | 1995 | Eritrea | Community | CS | Conv | Port workers | WB | 48 | 21.0 |
| Holt, 2003^68^ | 1992 | Ethiopia | Community | CS | Conv | Male Sudanese refugees | WB | 211 | 27.0 |
| Kapiga, 2003^32^ | 2000 | Tanzania | Community | CS | RS | Bars and hotels workers | ELISA | 515 | 43.5 |
| Kapiga, 2003^32^ | 2000 | Tanzania | Community | CS | RS | Non sexually active bar/hotel workers | ELISA | 22 | 0.0 |
| Kapiga, 2013^71^ | 2008-10 | Tanzania | Community | Cohort | Conv | Women working in food and recreational facilities | ELISA | 1,376 | 67.0 |
| Ng'ayo, 2008^95^ | 2005-06 | Kenya | Community | CS | CRS | Fishermen | ELISA | 250 | 63.9 |
| Rakwar, 1997^96^ | 1993 | Kenya | Outpatient clinic | Cohort | Conv | Male truck drivers | WB | 130 | 49.0 |
| Riedner, 2007^97^ | 2000-02 | Tanzania | Community | CS | Conv | Women working in bars, restaurants, and guesthouses | ELISA | 753 | 88.8 |
| Tassiopoulos, 2007^36^ | 2002-03 | Tanzania | Outpatient clinic | Cohort | Conv | Bar and hotel workers | ELISA | 1,045 | 56.3 |
| Vallely, 2007^98^ | 2002-04 | Tanzania | Community | CS | Conv | Women working in food and recreational facilities | ELISA | 1,563 | 74.6 |
| Watson-Jones, 2007^99^ | 2003-05 | Tanzania | Community | CS | Conv | Women working in service facilities | ELISA | 2,719 | 80.0 |
| **Higher-risk populations** | | | | | | | | | |
| Baeten, 2007^100^ | 1993 | Kenya | Outpatient clinic | Cohort | Conv | FSWs | ELISA | 1,206 | 80.6 |
| Baltzer, 2009^101^ | 2004-06 | Kenya | Outpatient clinic | Cohort | Conv | FSWs | ELISA | 139 | 87.1 |
| Braunstein, 2011a^56^ | 2006-07 | Rwanda | Community | CS | Conv | FSWs | ELISA | 800 | 59.8 |
| Ghebrekidan, 1999^61^ | 1995 | Eritrea | Outpatient clinic | CS | Conv | FSWs | WB | 107 | 80.0 |
| Holt, 2003^68^ | 1992 | Ethiopia | Community | CS | Conv | FSWs | WB | 203 | 65.0 |
| Jespers, 2014^69^ | 2010-11 | Rwanda | Community | CS | Conv | FSWs in Rwanda | ELISA | 30 | 47.0 |
| Kaul, 2007^39^ | 1998-02 | Kenya | Community | RCT | Conv | FSWs | ELISA | 443 | 72.7 |
| Masese, 2015^102^ | 1993-12 | Kenya | Community | Cohort | Conv | FSWs | ELISA | 1,964 | 73.4 |
| Priddy, 2011^103^ | 2008 | Kenya | Community | Cohort | Conv | FSWs | ELISA | 200 | 72.0 |
| Vandenhoudt, 2013^104^ | 1997 | Kenya | Community | CS | Conv | FSWs recruited in 1997 | ELISA | 286 | 93.4 |
| Vandenhoudt, 2013^104^ | 2008 | Kenya | Community | CS | Conv | FSWs recruited in 2008 | ELISA | 479 | 83.8 |
| Vandepitte, 2011^105^ | 2008-09 | Uganda | Community | Cohort | Conv | FSWs | ELISA | 1,026 | 80.0 |
| **HIV-negative populations** | | | | | | | | | |
| Mehta, 2008^106^ | 2002-05 | Kenya | Community | RCT | Conv | HIV negative healthy men | ELISA | 2,771 | 27.6 |
| Nakubulwa, 2009^107^ | 2005 | Uganda | Outpatient clinic | CC | Conv | HIV negative pregnant women | ELISA | 200 | 62.5 |
| Serwadda, 2003^108^ | 1994-98 | Uganda | Community | CC | Conv | HIV negative control group | ELISA/WB | 496 | 57.9 |
| **HIV-positive individuals and individuals in HIV discordant couples** | | | | | | | | | |
| Baeten, 2004^109^ | 1999-00 | Kenya | Outpatient clinic | RCT | Conv | HIV positive women enrolled in a trial | ELISA | 400 | 94.0 |
| Celum, 2014^49^ | 2008-10 | Kenya and Uganda | Community | RCT | RS | Partners of HIV positive patients | ELISA/WB | 4,638 | 67.2 |
| del Mar Pujades, 2002^12^ | 1991-94 | Tanzania | Community | CC | RS | HIV positive females enrolled in a large RCT | ELISA | 70 | 54.3 |
| del Mar Pujades, 2002^12^ | 1991-94 | Tanzania | Community | CC | RS | HIV positive males enrolled in a large RCT | ELISA | 57 | 61.4 |
| Jespers, 2014^69^ | 2010-11 | Rwanda | Community | CS | Conv | HIV positive +women | ELISA | 30 | 83.0 |
| Madebe, 2020^110^ | - | Tanzania | Hospital | CS | Conv | >10 years old HIV positive patients | ELISA | 180 | 18.0 |
| McClelland, 2002^111^ | 1996-99 | Kenya | Hospital | CS | Conv | HIV positive women | ELISA | 210 | 95.2 |
| Muiru, 2013^51^ | 2007-09 | Kenya | VCT | CS | Conv | Participants from HIV discordant couples | ELISA | 938 | 58.0 |
| Nakubulwa, 2009^107^ | 2005 | Uganda | Outpatient clinic | CC | Conv | HIV positive pregnant women | ELISA | 50 | 86.0 |
| Roxby, 2011^112^ | 1999-02 | Kenya | Outpatient clinic | Cohort | Conv | HIV positive pregnant women | ELISA | 296 | 85.8 |
| Serwadda, 2003^108^ | 1994-98 | Uganda | Community | CC | Conv | HIV seroconverters | ELISA/WB | 248 | 70.2 |
| Todd, 2006^90^ | 1994-95 | Tanzania | Community | Nested CC | RS | HIV positive women | ELISA | 37 | 70.3 |
| Todd, 2006^90^ | 1994-95 | Tanzania | Community | Nested CC | RS | HIV positive men | ELISA | 36 | 61.1 |
| **STI clinic attendees and symptomatic populations**^†^ | | | | | | | | | |
| Gorander, 2006^64^ | 2001 | Tanzania | Hospital | CS | Conv | Patients with GUD | WB | 198 | 78.3 |
| Langeland, 1998^113^ | 1989-93 | Tanzania | Outpatient clinic | CS | Conv | STI clinic attendees | ELISA | 294 | 42.9 |
| Mostad, 2000^114^ | 1994-96 | Kenya | Outpatient clinic | CS | Conv | Women attending an STI clinic | EIA | 314 | 93.3 |
| Mwansasu, 2002^115^ | - | Tanzania | Community | CS | Conv | Patients with GUD | ELISA | 69 | 79.7 |
| Nilsen, 2005^82^ | - | Tanzania | Outpatient clinic | CS | Conv | STI clinic attendees | ELISA | 494 | 70.4 |
| Suntoke, 2009^116^ | 2002-06 | Uganda | Community | CS | Conv | Males and females with GUD | ELISA | 95 | 77.0 |
| **Other populations** | | | | | | | | | |
| Kassa, 2019^117^ | 2005-09 | Ethiopia | Outpatient clinic/Hospital | CS | Conv | HIV negative and HIV positive pregnant women | ELISA | 2,532 | 41.5 |
| Okuku, 2011^52^ | 2005-08 | Kenya | Outpatient clinic | Cohort | Conv | Adults engaging in different risky sexual behaviors | ELISA | 1,272 | 32.6 |
| Van de Wijgert, 2009^30^ | 1999-04 | Uganda | Outpatient clinic | Cohort | Conv | FSWs and healthy women | ELISA | 2,199 | 48.7 |

^*^ The reported study design is the original study design (case control, cross sectional, longitudinal cohort, or randomized controlled trial). The included seroprevalence measures are those for the baseline measures at the beginning of the study.

^†^ Symptomatic populations include patients with clinical manifestations related to an STI.

Abbreviations: CC = Case control, Conv = Convenience, CRS = Cluster random sampling, CS = Cross sectional, DSS = Demographic surveillance system, EIA = Enzyme immunosorbent assay, ELISA = Enzyme-linked

immunosorbent assay, FSWs = Female sex workers, GUD = Genital ulcer disease, HIV = Human immunodeficiency virus, HSV-2 = Herpes simplex virus type 2, MOH = Ministry of Health, PROM = Premature rupture of membranes, RCT = Randomized controlled trial, RS = Random sampling, STI = Sexually transmitted infection, VCT = Voluntary counselling and testing, WB = Western blot.

# **Table S4.** Studies reporting HSV-2 seroprevalence in Southern Africa. This table includes only overall and not stratified seroprevalence measures,

| **Author, year** | **Year(s) of data collection** | **Country** | **Study site** | **Original study design^*^** | **Sampling method** | **Population** | **HSV-2 serological assay** | **Sample size** | **HSV-2 seroprevalence (%)** |
| --- | --- | --- | --- | --- | --- | --- | --- | --- | --- |
| **General populations** | | |  |  |  |  |  |  |  |
| Abbai, 2018^118^ | 2017 | South Africa | Outpatient clinic | CS | Conv | Pregnant women | ELISA | 248 | 71.0 |
| Abdool Karim, 2014^8^ | 2010 | South Africa | Community | CS | Conv | >14 years old males | ELISA | 1,252 | 2.6 |
| Abdool Karim, 2014^8^ | 2017 | South Africa | Community | CS | Conv | >13 years old females | ELISA | 1,423 | 10.7 |
| Achilles, 2016^119^ | - | Zimbabwe | Community | CS | Conv | 18-34 years old Zimbabwean women | ELISA | 200 | 34.0 |
| Austrian, 2016^120^ | 2016-16 | Zambia | Community | RCT | CRS | 15-19 years old females | ELISA | 2,360 | 7.0 |
| Baird, 2012^121^ | 2007 | Malawi | Community | RCT | CRS | Schoolgirls as a control group | RDT | 796 | 3.0 |
| Baird, 2012^121^ | 2007 | Malawi | Community | RCT | CRS | School dropouts as a control group | RDT | 208 | 8.0 |
| Birdthistle, 2008^122^ | 2004 | Zimbabwe | Community | CS | CRS | 14-20 years old females | ELISA | 746 | 11.7 |
| Bradley, 2018^123^ | 2013-15 | South Africa | Community | RCT | RS | South African women | ELISA | 12,179 | 60.0 |
| Bradley, 2018^123^ | 2013-15 | South Africa | Community | RCT | RS | South African men | ELISA | 5,472 | 27.0 |
| Bradley, 2018^123^ | 2013-15 | Zambia | Community | RCT | RS | Zambian women | ELISA | 14,002 | 50.0 |
| Bradley, 2018^123^ | 2013-15 | Zambia | Community | RCT | RS | Zambian men | ELISA | 5,309 | 22.0 |
| Chatterjee, 2010^124^ | 1998-01 | South Africa | Outpatient clinic | CC | Conv | Women without cervical cancer | ELISA | 407 | 65.1 |
| Cowan, 2008b^125^ | 2003 | Zimbabwe | Community | RCT | RS | Healthy students | ELISA | 6,791 | 0.2 |
| Crucitti, 2011^126^ | - | Zambia | Community | CS | Conv | 13-16 years old females | ELISA | 450 | 5.6 |
| De Baetselier, 2015^11^ | 2009-10 | South Africa | Outpatient clinic | Cohort | Conv | Healthy women | ELISA | 701 | 41.1 |
| Delany-Moretlwe, 2010^127^ | 2003 | South Africa | Outpatient clinic | CS | Conv | Women at a family planning clinic | ELISA | 210 | 65.7 |
| Fearon, 2017^128^ | 2011-12 | South Africa | Community | RCT | Conv | 13-20 years old students | ELISA | 2,326 | 4.6 |
| Ferrand, 2010a^129^ | 2009 | Zimbabwe | Outpatient clinic | CS | Conv | Primary health care attendees | ELISA | 506 | 4.2 |
| Ferrand, 2010a^129^ | 2009 | Zimbabwe | Outpatient clinic | CS | Conv | ANC attendees | ELISA | 88 | 14.0 |
| Ferrand, 2010b^130^ | 2007-08 | Zimbabwe | Hospital | CS | Conv | 10-18 years old patients | ELISA | 301 | 1.3 |
| Francis, 2018^131^ | 2016-17 | South Africa | Community | CS | RS | 15-24 years old females | ELISA | 259 | 28.7 |
| Francis, 2018^131^ | 2016-17 | South Africa | Community | CS | RS | 15-24 years old males | ELISA | 188 | 16.8 |
| Glynn, 2008^132^ | 1988-90 | Malawi | Community | CS | Conv | Sample collected from women in 1988-90 | ELISA | 343 | 56.0 |
| Glynn, 2008^132^ | 1988-90 | Malawi | Community | CS | Conv | Samples collected from men in 1988-90 | ELISA | 334 | 33.2 |
| Glynn, 2008^132^ | 1998-01 | Malawi | Community | CS | Conv | Samples collected from women in 1998-01 | ELISA | 384 | 66.7 |
| Glynn, 2008^132^ | 1998-01 | Malawi | Community | CS | Conv | Samples collected from men in 1998-01 | ELISA | 335 | 57.0 |
| Glynn, 2008^132^ | 2002-05 | Malawi | Community | CS | Conv | Samples collected from women in 2002-05 | ELISA | 205 | 55.6 |
| Glynn, 2008^132^ | 2002-05 | Malawi | Community | CS | Conv | Samples collected from men in 2002-05 | ELISA | 178 | 42.1 |
| Glynn, 2008^132^ | 1999-00 | Malawi | Community | CS | Conv | Samples collected from ANC attendees in 1999-00 | ELISA | 981 | 47.6 |
| Glynn, 2014^133^ | 2007 | Malawi | Community | CS | Conv | 15-30 years old women | ELISA | 3,419 | 25.5 |
| Gray, 2011^134^ | 2007 | South Africa | Community | RCT | Conv | >18 years old healthy individuals | WB | 801 | 31.0 |
| Gregson, 2001^135^ | 1998 | Zimbabwe | Community | CS | Conv | Healthy population | ELISA | 144 | 63.2 |
| Gwanzura, 2002^136^ | 2006 | Zimbabwe | Community | CS | RS | Blood donors | IB | 299 | 9.7 |
| Hazel, 2015^137^ | 2009 | Namibia | Community | CS | Conv | Mobile rural pastoralists | RDT | 402 | 35.0 |
| Heffron, 2011^14^ | 2006-07 | Zambia | Community | Cohort | Conv | Male sugar cane farmers | ELISA | 1,062 | 54.4 |
| Heffron, 2011^14^ | 2006-07 | Zambia | Community | Cohort | Conv | Male migrant farmers | ELISA | 498 | 62.5 |
| Heffron, 2011^14^ | 2006-07 | Zambia | Community | Cohort | Conv | Male non-migrant farmers | ELISA | 564 | 47.3 |
| Jespers, 2014^69^ | 2010-11 | South Africa | Outpatient clinic | CS | Conv | South African women | ELISA | 109 | 40.0 |
| Jespers, 2014^69^ | 2010-11 | South Africa | Outpatient clinic | CS | Conv | South African pregnant women | ELISA | 30 | 37.0 |
| Jespers, 2014^69^ | 2010-11 | South Africa | Outpatient clinic | CS | Conv | South African adolescents | ELISA | 30 | 3.0 |
| Jespers, 2014^69^ | 2010-11 | South Africa | Outpatient clinic | CS | Conv | South African women engaging in vaginal practices | ELISA | 31 | 45.0 |
| Jewkes, 2008^15^ | 2003-04 | South Africa | Community | RCT | CRS | Women in the intervention arm in a trial | ELISA | 715 | 27.6 |
| Jewkes, 2008^15^ | 2003-04 | South Africa | Community | RCT | CRS | Women in the control arm in a trial | ELISA | 701 | 31.0 |
| Jewkes, 2008^15^ | 2003-04 | South Africa | Community | RCT | CRS | Men in the intervention arm in a trial | ELISA | 694 | 10.3 |
| Jewkes, 2008^15^ | 2003-04 | South Africa | Community | RCT | CRS | Men in the control arm in a trial | ELISA | 666 | 10.0 |
| Kapina, 2009^138^ | 2003-04 | Zambia | Community | Cohort | Conv | Healthy women | ELISA | 239 | 38.9 |
| Kenyon, 2013^139^ | 2000 | South Africa | Community | CS | RS | Students or unemployed women | ELISA | 771 | 53.3 |
| Kenyon, 2013^139^ | 2000 | South Africa | Community | CS | RS | Students or unemployed men | ELISA | 718 | 17.0 |
| Kharsany, 2020^140^ | 2014-15 | South Africa | Community | CS | RS | Healthy adults | ELISA | 9,786 | 57.8 |
| Kjetland, 2005^141^ | 1998-99 | Zimbabwe | Community | CS | Conv | Women in rural Zimbabwe | ELISA | 476 | 64.5 |
| Kurewa, 2010^142^ | 2002-03 | Zimbabwe | Outpatient clinic | CS | Conv | Pregnant women | ELISA | 678 | 51.1 |
| Luseno, 2014^143^ | 2012 | Zimbabwe | Community | CS | Conv | Female adolescent orphans | ELISA | 287 | 6.0 |
| Mbizvo, 2002^144^ | 1999-00 | Zimbabwe | Outpatient clinic | CS | Conv | Women presenting to polyclinics | ELISA | 389 | 42.2 |
| McFarland, 1999^19^ | 1993-97 | Zimbabwe | Community | CS | Conv | >18 years old male factory workers | IB | 2,397 | 39.8 |
| Menezes, 2018^145^ | 2012-13 | South Africa | Outpatient clinic | RCT | Conv | Young healthy females | ELISA | 388 | 46.0 |
| Munjoma, 2010^21^ | 2002 | Zimbabwe | Outpatient clinic | CS | Conv | Husbands of pregnant women | ELISA | 43 | 46.0 |
| NIMH Collaborative, 2007^146^ | 2001 | Zimbabwe | Community | CS | RS | 16-30 years old healthy women | ELISA | 891 | 58.6 |
| NIMH Collaborative, 2007^146^ | 2001 | Zimbabwe | Community | CS | RS | 16-30 years old healthy men | ELISA | 683 | 26.6 |
| Pascoe, 2015^147^ | 2007 | Zimbabwe | Community | CS | RS | 18-22 years old females | ELISA | 2,505 | 11.2 |
| Price, 2016^148^ | 2011-12 | South Africa | Community | RCT | RS | Females going to school | ELISA | 2,533 | 5.0 |
| Sobngwi-Tambekou, 2009^26^ | 2002-04 | South Africa | Community | RCT | RS | Healthy males | ELISA | 3,274 | 5.9 |
| Mensch, 2020^20^ | 2007-13 | Malawi | Community | Cohort | RS | School students | ELISA | 2,392 | 13.4 |
| van de Wijgert, 2009^30^ | 1999-04 | Zimbabwe | Outpatient clinic | Cohort | Conv | 18-35 years old women | ELISA | 2,240 | 53.2 |
| Wand, 2012^149^ | 2002-05 | South Africa | Community | Cohort | Conv | Women in Durban | ELISA | 3,492 | 73.0 |
| Weiss, 2001^91^ | 1997-98 | Zambia | Community | CS | CRS | Women from Ndola | ELISA | 885 | 55.0 |
| Weiss, 2001^91^ | 1997-98 | Zambia | Community | CS | CRS | Men from Ndola | ELISA | 607 | 36.0 |
| **Intermediate-risk population**s | | | | | | | |  |  |
| Meque, 2014^33^ | 2009-12 | Mozambique | Community | CS | Conv | Women working in service facilities | ELISA | 409 | 60.6 |
| **Higher-risk populations** | | | | | | | | | |
| Cowan, 2005^150^ | - | Zimbabwe | Community | CS | Conv | FSWs | ELISA | 369 | 77.8 |
| Ramjee, 2005^41^ | 1996-97 | South Africa | Community | RCT | Conv | FSWs | ELISA | 416 | 84.0 |
| **HIV-negative populations** | | | | | | | | | |
| Abdool Karim, 2015^151^ | 2007-10 | South Africa | Community | Cohort | Conv | HIV negative women | ELISA | 888 | 51.4 |
| Balkus, 2016^152^ | 2005-08 | 4 African countries^†^ | Community | RCT | Conv | Adult women from 4 African countries | ELISA | 2,830 | 42.0 |
| Barnabas, 2018^153^ | 2013-15 | South Africa | Community | Cohort | Conv | Young women from Cape Town | ELISA | 149 | 21.0 |
| Benjamin, 2008^154^ | 2005 | South Africa | Community | CC | Conv | HIV negative blood donors | ELISA | 102 | 19.6 |
| Benjamin, 2008^154^ | 2005 | South Africa | Community | CS | Conv | Blood donors in 2005 | ELISA | 200 | 8.5 |
| Gust, 2016^155^ | 2007-10 | Botswana | Community | RCT | Conv | All male and female participants in a study | ELISA | 1,201 | 35.6 |
| Mavedzenge, 2011^156^ | 2003-05 | South Africa | Community | Cohort | Conv | Women in Johannesburg | ELISA | 1,008 | 65.0 |
| Mavedzenge, 2011^156^ | 2003-05 | Zimbabwe | Community | Cohort | Conv | Women in Harare | ELISA | 2,455 | 51.0 |
| Mlisana, 2012^47^ | 2004-05 | South Africa | Outpatient clinic | CS | Conv | Healthy women | ELISA | 242 | 86.0 |
| Perti, 2014^48^ | - | South Africa | Hospital | CS | Conv | HIV negative pregnant women | ELISA/WB | 255 | 43.5 |
| Wand, 2013^157^ | 2003-06 | South Africa | Community | Cohort | Conv | Women born before 1960 | ELISA | 110 | 89.0 |
| Wand, 2013^157^ | 2003-06 | South Africa | Community | Cohort | Conv | Women born between 1960-1964 | ELISA | 257 | 81.8 |
| Wand, 2013^157^ | 2003-06 | South Africa | Community | Cohort | Conv | Women born between 1965-1969 | ELISA | 409 | 81.6 |
| Wand, 2013^157^ | 2003-06 | South Africa | Community | Cohort | Conv | Women born between 1970-1974 | ELISA | 559 | 81.4 |
| Wand, 2013^157^ | 2003-06 | South Africa | Community | Cohort | Conv | Women born between 1975-1979 | ELISA | 770 | 76.7 |
| Wand, 2013^157^ | 2003-06 | South Africa | Community | Cohort | Conv | Women born between 1980-1984 | ELISA | 1,043 | 66.2 |
| Wand, 2013^157^ | 2003-06 | South Africa | Community | Cohort | Conv | Women born after 1985 | ELISA | 324 | 43.3 |
| Sutcliffe, 2002^158^ | 1998 | Malawi | Community | CC | Conv | HIV negative males | ELISA | 280 | 64.3 |
| **HIV-positive individuals and individuals in HIV discordant couples** | | | | | | | | | |
| Benjamin, 2008^154^ | 2005 | South Africa | Community | CC | Conv | HIV positive blood donors | ELISA | 106 | 72.6 |
| Benjamin, 2008^154^ | 2001-02 | South Africa | Community | CS | Conv | HIV positive blood donors in 2001-2002 | ELISA | 625 | 69.3 |
| Cowan, 2008a^50^ | 1997-00 | Zimbabwe | Outpatient clinic | CC | RS | HIV positive mothers with HIV positive infants | ELISA/WB | 478 | 86.2 |
| Cowan, 2008a^50^ | 1997-00 | Zimbabwe | Outpatient clinic | CC | RS | HIV positive mothers with HIV negative infants | ELISA/WB | 970 | 80.7 |
| Lewis, 2012^159^ | 2007 | South Africa | Outpatient clinic | CS | Conv | HIV positive patients | ELISA | 1,106 | 85.2 |
| Lowe, 2019^160,161^ | 2016 | South Africa | Outpatient clinic | CS | Conv | HIV positive women | ELISA | 385 | 52.5 |
| Perti, 2014^48^ | - | South Africa | Hospital | CS | Conv | HIV positive pregnant women | ELISA/WB | 132 | 87.9 |
| Rabenau, 2010^162^ | 2007 | Lesotho | Outpatient clinic | CS | Conv | HIV positive patients | ELISA | 205 | 78.5 |
| Sutcliffe, 2002^158^ | 1998 | Malawi | Community | CC | Conv | HIV positive males | ELISA | 279 | 88.1 |
| Varo, 2016^163^ | 2008-10 | Malawi | Hospital | CS | RS | <14 years old HIV positive children | ELISA | 91 | 4.4 |
| **STI clinic attendees and symptomatic populations**^‡^ | | | | | | | | | |
| Chen, 2000^164^ | 1993-94 | South Africa | Outpatient clinic | CS | Conv | Men with GUD | WB | 498 | 49.2 |
| Chen, 2000^164^ | 1993-94 | South Africa | Outpatient clinic | CS | Conv | Men with urethritis | WB | 554 | 42.1 |
| Esber, 2017^165^ | 2015 | Malawi | Outpatient clinic | CS | Conv | Symptomatic women attending clinic | ELISA | 197 | 50.0 |
| Hoyo, 2005^166^ | 1998-99 | Malawi | Outpatient clinic | CS | Conv | STI clinic attendees with GUD | WB | 136 | 80.0 |
| Kufa, 2020^161^ | 2017-19 | South Africa | Outpatient clinic | CS | Conv | Men attending an STI clinic | ELISA | 847 | 51.0 |
| Kularatne, 2018^167^ | 2007-15 | South Africa | Outpatient clinic | CS | Conv | Patients attending an STI clinic | ELISA | 771 | 80.2 |
| Lewis, 2008^168^ | 2006 | South Africa | Community | CS | Conv | Male STI clinic attendees | ELISA | 303 | 57.1 |
| Lewis, 2013^169^ | 2007-12 | South Africa | Outpatient clinic | CS | Conv | Men with urethral discharge | ELISA | 1,204 | 55.6 |
| Lewis, 2013^169^ | 2007-12 | South Africa | Outpatient clinic | CS | Conv | Women with vaginal discharge | ELISA | 1,216 | 77.7 |
| Mhlongo, 2010^170^ | 2006-07 | South Africa | Outpatient clinic | CS | Conv | Females in Cape Town with VDS | ELISA | 92 | 38.0 |
| Mhlongo, 2010^170^ | 2006-07 | South Africa | Outpatient clinic | CS | Conv | Males in Cape Town with MUS Syndrome | ELISA | 279 | 30.5 |
| Mhlongo, 2010^170^ | 2006-07 | South Africa | Outpatient clinic | CS | Conv | Females in Johannesburg with VDS | ELISA | 199 | 75.9 |
| Mhlongo, 2010^170^ | 2006-07 | South Africa | Outpatient clinic | CS | Conv | Males in Johannesburg with MUS Syndrome | ELISA | 211 | 59.7 |
| Morse, 1997^171^ | 1993-94 | Lesotho | Outpatient clinic | CS | Conv | Patients with GUD | WB | 99 | 55.0 |
| O'Farrell, 2007^172^ | 2004 | South Africa | Outpatient clinic | CS | Conv | Males with GUD | ELISA | 162 | 86.4 |
| O'Farrell, 2007^172^ | 2004 | South Africa | Outpatient clinic | CS | Conv | Males attending an STI clinic | ELISA | 480 | 72.3 |
| Paz-Bailey, 2009^173^ | 2005-06 | South Africa | Outpatient clinic | RCT | RS | Men with GUD | ELISA | 615 | 70.6 |
| Phiri, 2013^174^ | 2004-06 | Malawi | Outpatient clinic | RCT | Conv | Males and females with GUD | ELISA | 417 | 72.0 |
| Zimba, 2011^175^ | 2005 | Mozambique | Outpatient clinic | CS | Conv | Patients attending a clinic with US or VDS | ELISA | 346 | 85.0 |
| **Other populations** | | | | | | | | | |
| Chattopadhyay, 2015^176^ | 1998-01 | South Africa | Community | CC | Conv | Women with cervical cancer | ELISA | 429 | 68.0 |

^*^ The reported study design is the original study design (case control, cross sectional, longitudinal cohort, or randomized controlled trial). The included seroprevalence measures are those for the baseline measures at the beginning of the study.

^†^ The four African countries are: Malawi, South Africa, Zambia, and Zimbabwe.

^‡^ Symptomatic populations include patients with clinical manifestations related to an STI.

Abbreviations: ANC = Antenatal clinic, CC = Case control, Conv = Convenience, CRS = Cluster random sampling, CS = Cross sectional, DSS = Demographic surveillance system, EIA = Enzyme immunosorbent assay, ELISA = Enzyme-linked immunosorbent assay, FSWs = Female sex workers, GUD = Genital ulcer disease, HIV = Human immunodeficiency virus, HSV-2 = Herpes simplex virus type 2, IB = Immunoblot, US = Urethritis syndrome, NIMH = National Institute of Mental Health, RCT = Randomized controlled trial, RDT = Rapid diagnostic test, RS = Random sampling, STI = Sexually transmitted infection, VCT = Voluntary counselling and testing, VDS = Vaginal discharge syndrome, WB = Western blot.

# **Table S5.** Studies reporting HSV-2 seroprevalence in Western Africa. This table includes only overall and not stratified seroprevalence measures.

| **Author, year** | **Year(s) of data collection** | **Country** | **Study site** | **Original study design^*^** | **Sampling method** | **Population** | **HSV-2 serological assay** | **Sample size** | **HSV-2 seroprevalence (%)** |
| --- | --- | --- | --- | --- | --- | --- | --- | --- | --- |
| **General populations** | | |  |  |  |  |  |  |  |
| Anaedobe, 2019^177^ | 2013 | Nigeria | Outpatient clinic | CS | Conv | 22-44 years old ANC attendees | ELISA | 270 | 33.3 |
| Ashley-Morrow, 2004^178^ | 2000-01 | Nigeria | Community | CS | Conv | >15 years old women | ELISA | 1,268 | 62.0 |
| Béhanzin, 2012^179^ | 2008 | Benin | Community | CS | CS | General population in 2008 | ELISA | 2,193 | 26.4 |
| Cisse, 2015^180^ | 2011-12 | Cote D'Ivoire | Outpatient clinic | CS | RS | Pregnant women | ELISA | 170 | 96.5 |
| Cisse, 2015^180^ | 2011-12 | Cote D'Ivoire | Outpatient clinic | CS | RS | ≥30 years old pregnant women | ELISA | 53 | 94.3 |
| Eltom, 2002^181^ | 1991-94 | Nigeria | Outpatient clinic | CS | Conv | >15 healthy females | WB | 287 | 17.1 |
| Eltom, 2002^181^ | 1991-94 | Nigeria | Community | CS | Conv | Healthy males | WB | 479 | 11.5 |
| Kane, 2009^182^ | 2003 | Senegal | Outpatient clinic | CS | Conv | Pregnant women approached in 2003 | ELISA | 260 | 21.5 |
| Kane, 2009^182^ | 2006 | Senegal | Outpatient clinic | CS | Conv | Pregnant women approached in 2006 | ELISA | 628 | 13.2 |
| Katz, 2020^183^ | - | Ghana | Community | CS | Conv | Healthy adults | ELISA | 406 | 33.3 |
| Kirakoya-Samadoulougou, 2011^184^ | 2003 | Burkina Faso | Outpatient clinic | CS | Conv | Pregnant women from antenatal clinics | ELISA | 2,018 | 17.9 |
| Kirakoya-Samadoulougou, 2011^184^ | 2003 | Burkina Faso | Community | CS | CRS | 15-49 years old women from urban populations | ELISA | 883 | 23.6 |
| Kirakoya-Samadoulougou, 2011^184^ | 2003 | Burkina Faso | Community | CS | CRS | 15-49 years old men from urban populations | ELISA | 791 | 15.2 |
| Lagarde, 2004^185^ | 2000 | Burkina Faso | Community | CS | CRS | Healthy men | ELISA | 1,030 | 12.5 |
| Lagarde, 2004^185^ | 2000 | Burkina Faso | Community | CS | CRS | Healthy women | ELISA | 1,210 | 24.0 |
| Mawak, 2012^186^ | - | Nigeria | Community | CS | RS | Healthy adults | ELISA | 180 | 24.4 |
| Patnaik, 2007^187^ | 1985-97 | Mali | Hospital | CC | Conv | Controls in a case-control study of cervical cancer | WB | 90 | 43.3 |
| Shaw, 2001^188^ | 1998 | Gambia | Community | CS | CRS | 15-34 years old men | ELISA | 432 | 5.1 |
| Shaw, 2001^188^ | 1998 | Gambia | Community | CS | CRS | 15-34 years old women | ELISA | 575 | 28.5 |
| Thomas, 2004^189^ | 1999-00 | Nigeria | Community | CS | Conv | >15 years old sexually active women | ELISA | 892 | 61.3 |
| Walraven, 2001^190^ | 1999 | Gambia | Community | CS | CRS | ≥15 years old healthy women | ELISA | 1,317 | 31.9 |
| Weiss, 2001^91^ | 1997-98 | Benin | Community | CS | CRS | Women from Cotonou | ELISA | 935 | 30.0 |
| Weiss, 2001^91^ | 1997-98 | Benin | Community | CS | CRS | Men from Cotonou | ELISA | 863 | 12.0 |
| **Higher-risk populations** | | | | | | | | | |
| Aho, 2014^191^ | 2006 | Guinea | Outpatient clinic | CS | Conv | FSWs | ELISA | 201 | 84.1 |
| Dada, 1998^192^ | 1990-91 | Nigeria | Community | CS | Conv | FSWs | WB | 796 | 59.0 |
| Eltom, 2002^181^ | 1991-94 | Nigeria | Community | CS | Conv | FSWs | WB | 863 | 60.6 |
| Kane, 2009^182^ | 2006 | Senegal | Community | CS | Conv | FSWs | ELISA | 604 | 88.4 |
| Low, 2011^193^ | 2003-06 | Burkina Faso | Community | Cohort | Conv | FSWs | ELISA | 689 | 62.4 |
| Nagot, 2005^194^ | 198-02 | Burkina Faso | Community | Cohort | Conv | FSWs | ELISA | 329 | 54.7 |
| Traore, 2015^195^ | 2009-11 | Burkina Faso | Community | RCT | Conv | FSWs | ELISA | 321 | 28.0 |
| Wade, 2005^196^ | 2004 | Senegal | Community | CS | Conv | MSM | ELISA | 440 | 22.3 |
| **HIV-negative populations** | | | | | | | | | |
| Camara, 2012^197^ | - | Senegal | Outpatient clinic | CS | Conv | HIV negative Senegalese males | ELISA | 35 | 11.0 |
| Camara, 2012^197^ | - | Senegal | Outpatient clinic | CS | Conv | HIV negative Senegalese females | ELISA | 35 | 23.0 |
| **HIV-positive individuals and individuals in HIV discordant couples** | | | | | | | | | |
| Camara, 2012^197^ | - | Senegal | Outpatient clinic | CS | Conv | Male partners in HIV discordant couples | ELISA | 34 | 44.0 |
| Camara, 2012^197^ | - | Senegal | Outpatient clinic | CS | Conv | Female partners in HIV discordant couples | ELISA | 34 | 56.0 |
| Camara, 2012^197^ | - | Senegal | Outpatient clinic | CS | Conv | HIV positive males | ELISA | 35 | 60.0 |
| Camara, 2012^197^ | - | Senegal | Outpatient clinic | CS | Conv | HIV positive females | ELISA | 35 | 63.0 |
| Obedisi-Omokanye, 2017^198^ | - | Nigeria | Outpatient clinic | CS | Conv | HIV positive patients | ELISA | 276 | 42.0 |
| Yunusa, 2019^199^ | 2014-16 | Nigeria | Outpatient clinic | CS | Conv | HIV positive patients | ELISA | 16 | 50.0 |
| **STI clinic attendees and symptomatic populations**^†^ | | | | | | | | | |
| Aryee, 2005^200^ | 2004 | Gambia | Outpatient clinic | CS | Conv | Women attending an STI clinic | ELISA | 63 | 46.0 |
| Eltom, 2002^181^ | 1991-94 | Nigeria | Outpatient clinic | CS | Conv | Females attending an STI clinics | WB | 114 | 44.7 |
| Eltom, 2002^181^ | 1991-94 | Nigeria | Outpatient clinic | CS | Conv | Males attending an STI clinics | WB | 259 | 14.7 |

^*^ The reported study design is the original study design (case control, cross sectional, longitudinal cohort, or randomized controlled trial). The included seroprevalence measures are those for the baseline measures at the beginning of the study.

^†^ Symptomatic populations include patients with clinical manifestations related to an STI.

Abbreviations: ANC = Antenatal clinic, CC = Case control, Conv = Convenience, CRS = Cluster random sampling, CS = Cross sectional, ELISA = Enzyme-linked immunosorbent assay, FSWs = Female sex workers, HIV = Human immunodeficiency virus, HSV-2 = Herpes simplex virus type 2, MSM = Men who have sex with men, RCT = Randomized controlled trial, RS = Random sampling, STI = Sexually transmitted infection, WB = Western blot.

# **Table S6.** Studies reporting HSV-2 seroprevalence in Central Africa. This table includes only overall and not stratified seroprevalence measures.

| **Author, year** | **Year(s) of data collection** | **Country** | **Study site** | **Original study design^*^** | **Sampling method** | **Population** | **HSV-2 serological assay** | **Sample size** | **HSV-2 seroprevalence (%)** |
| --- | --- | --- | --- | --- | --- | --- | --- | --- | --- |
| **General populations** | | |  |  |  |  |  |  |  |
| Charpentier, 2011^201^ | 2007 | Chad | Community | CS | CRS | General adult population | ELISA | 548 | 15.7 |
| Eis-Hubinger, 2002^202^ | 1998 | Cameroon | Outpatient clinic | CS | RS | Patients attending a pulmonary clinic | WB | 188 | 72.3 |
| Ozouaki, 2006^203^ | - | Gabon | Outpatient clinic | CS | Conv | Women of childbearing age | ELISA | 355 | 65.9 |
| Volpi, 2004^204^ | 1997-98 | Cameroon | Outpatient clinic | CS | Conv | Males and females attending general clinic | ELISA | 238 | 34.0 |
| Weiss, 2001^91^ | 1997-98 | Cameroon | Community | CS | CRS | Women from Yaounde | ELISA | 1,002 | 51.0 |
| Weiss, 2001^91^ | 1997-98 | Cameroon | Community | CS | CRS | Men from Yaounde | ELISA | 887 | 27.0 |
| **Higher-risk populations** | | | | | | | | | |
| Longo, 2017^205^ | 2013 | Central African Republic | Community | CS | Conv | FSWs | ELISA | 345 | 11.9 |
| Nzila, 1991^206^ | 1988 | Democratic Republic of Congo | Community | CS | Conv | FSWs | WB | 1,233 | 21.5 |
| **HIV-negative populations** | | | | | | | | | |
| LeGoff, 2011^207^ | 2009 | Central African Republic | Hospital | CS | Conv | ≤17 years old HIV negative children | ELISA | 200 | 6.0 |
| **STI clinic attendees and symptomatic populations**^†^ | | | | | | | | | |
| Eis-Hubinger, 2002^202^ | 1998 | Cameroon | Outpatient clinic | CS | RS | Patients attending an STI clinic | EIA | 161 | 67.1 |
| Mbopi-keou, 2000^208^ | - | Central African Republic | Outpatient clinic | CS | Conv | Women attending an STI clinic | ELISA | 300 | 82.0 |
| Vandepitte, 2007^209^ | 2002 | Democratic Republic of Congo | Outpatient clinic | CS | Conv | FSWs attending an STI clinic | ELISA | 501 | 58.5 |
| **Other populations** | | | | | | | | | |
| Eis-Hubinger, 2002^202^ | 1998 | Cameroon | Hospital | CS | RS | Patients with symptoms suggestive of HIV | EIA | 61 | 70.5 |

^*^ The reported study design is the original study design (case control, cross sectional, longitudinal cohort, or randomized controlled trial). The included seroprevalence measures are those for the baseline measures at the beginning of the study.

^†^ Symptomatic populations include patients with clinical manifestations related to an STI.

Abbreviations: Conv = Convenience, CRS = Cluster random sampling, CS = Cross sectional, EIA = Enzyme immunosorbent assay, ELISA = Enzyme-linked immunosorbent assay, FSWs = Female sex workers, HIV = Human immunodeficiency virus, HSV-2 = Herpes simplex virus type 2, RS = Random sampling, STI = Sexually transmitted infection, WB = Western blot.

# **Table S7.** Studies reporting HSV-2 seroprevalence across several regions in sub-Saharan Africa. This table includes only overall and not stratified seroprevalence measures.

| **Author, year** | **Year(s) of data collection** | **Country** | **Study site** | **Original study design^*^** | **Sampling method** | **Population** | **HSV-2 serological assay** | **Sample size** | **HSV-2 seroprevalence (%)** |
| --- | --- | --- | --- | --- | --- | --- | --- | --- | --- |
| **HIV-negative populations** | | |  |  |  |  |  |  |  |
| ECHO trial consortium^210^ | 2015-17 | 4 African countries^†^ | Community | RCT | Conv | Females using injectable contraceptives | ELISA | 2,588 | 38.7 |
| ECHO trial consortium^210^ | 2015-17 | 4 African countries^†^ | Community | RCT | Conv | Females using intrauterine contraceptives | ELISA | 2,587 | 39.4 |
| ECHO trial consortium^210^ | 2015-17 | 4 African countries^†^ | Community | RCT | Conv | Females using implantable contraceptives | ELISA | 2,584 | 37.4 |
| Marrazzo, 2015^211^ | 2009-11 | 3 African countries^‡^ | Community | RCT | Conv | Female trial participants | ELISA | 5,005 | 46.0 |
| McCormack, 2010^44^ | 2005-09 | 4 African countries^§^ | Outpatient clinic/Community | RCT | Conv | Female trial participants receiving 2% gel | ELISA | 2,725 | 60.0 |
| McCormack, 2010^44^ | 2005-09 | 4 African countries^§^ | Outpatient clinic/Community | RCT | Conv | Female trial participants receiving 0.5% gel | ELISA | 3,312 | 61.0 |
| McCormack, 2010^44^ | 2005-09 | 4 African countries^§^ | Outpatient clinic/Community | RCT | Conv | Female trial participants receiving placebo | ELISA | 3,311 | 60.0 |
| **HIV-positive individuals and individuals in HIV discordant couples** | | | | | | | | | |
| Heffron, 2012^212^ | 2004-10 | 7 African countries^¶^ | Community | Cohort | Conv | Males with an HIV positive partner | WB | 2,393 | 60.2 |
| Heffron, 2012^212^ | 2004-10 | 7 African countries^¶^ | Community | Cohort | Conv | Females with an HIV positive partner | WB | 1,283 | 84.8 |
| **STI clinic attendees and symptomatic populations**^‖^ | | | | | | | | | |
| LeGoff, 2007^213^ | 2003-05 | 2 African countries^**^ | Outpatient clinic | RCT | Conv | Women with GUD | ELISA | 437 | 79.0 |

^*^ The reported study design is the original study design (case control, cross sectional, longitudinal cohort, or randomized controlled trial). The included seroprevalence measures are those for the baseline measures at the beginning of the study.

^†^ The four African countries are Eswatini, South Africa, Kenya, and Zambia.

^‡^ The three African countries are South Africa, Uganda, and Zimbabwe.

^§^ The four African countries are South Africa, Tanzania, Uganda, and Zimbabwe.

^¶^ The seven African countries are: Botswana, Kenya, South Africa, Tanzania, Uganda, and Zambia.

^‖^ Symptomatic populations include patients with clinical manifestations related to a sexually transmitted infection.

^**^ The two African countries are Ghana and Central African Republic

Abbreviations: Conv = Convenience, ELISA = Enzyme-linked immunosorbent assay, GUD = Genital ulcer disease, HIV = Human immunodeficiency virus, HSV-2 = Herpes simplex virus type 2, RCT = Randomized controlled trial, WB = Western blot.

# **Table S8.** Pooled mean estimates for herpes simplex virus type 2 seroprevalence among general populations by sex stratification in sub-Saharan Africa.

|  | |  |  | **Among women** | |  |  |  |  |  | | **Among men** |  |
| --- | --- | --- | --- | --- | --- | --- | --- | --- | --- | --- | --- | --- | --- |
|  | | **Outcome measures** | **Sample size** | **HSV-2 seroprevalence (%)** | | **Pooled mean (%) HSV-2 seroprevalence** | **I²^*^ (%)** | **Outcome measures** | **Sample size** | **HSV-2 seroprevalence (%)** | | **Pooled mean (%) HSV-2 seroprevalence** | **I²^*^ (%)** |
|  | | **Total n** | **Total N** | **Range** | **Median** | **(95% CI)** | **(95% CI)** | **Total n** | **Total N** | **Range** | **Median** | **(95% CI)** | **(95% CI)** |
| **Countries** | Benin | 8 | 2,130 | 9.0-57.0 | 34.6 | 34.3 (24.1-45.3) | 95.0 (92.3-96.8) | 8 | 1,861 | 1.0-42.0 | 17.5 | 15.6 (8.0-25.1) | 94.9 (92.0-96.7) |
|  | Burkina Faso | 9 | 4,111 | 6.9-40.5 | 20.6 | 21.4 (15.4-28.0) | 95.5 (93.2-97.0) | 5 | 1,821 | 4.1-37.5 | 12.5 | 16.3 (8.8-25.4) | 94.5 (90.0-97.0) |
|  | Cameroon | 8 | 1,125 | 14.0-78.0 | 69.0 | 58.0 (39.1-100) | 97.5 (96.4-98.3) | 8 | 996 | 3.0-59.0 | 34.5 | 31.5 (15.4-50.2) | 97.2 (96.0-98.1) |
|  | Ethiopia | 6 | 744 | 24.4-59.5 | 33.3 | 36.4 (22.4-41.8) | 92.8 (87.1-96.0) | - | - | - | - | - | - |
|  | Gambia | 5 | 1,892 | 7.8-47.7 | 31.9 | 28.7 (17.4-41.7) | 95.4 (91.9-97.4) | 4 | 431 | 2.6-16.4 | 4.1 | 5.5 (1.6-11.3) | 76.8 (36.5-91.5) |
|  | Kenya | 40 | 18,863 | 4.4-91.3 | 51.0 | 50.2 (42.2-58.2) | 99.1 (99.0-99.2) | 30 | 15,603 | 1.9-76.0 | 33.1 | 27.9 (21.0-35.4) | 98.8 (98.6-99.0) |
|  | Malawi | 33 | 7,287 | 3.0-87.5 | 56.0 | 48.7 (39.6-57.9) | 98.1 (97.8-98.4) | 22 | 2,013 | 0.0-77.3 | 46.5 | 39.2 (26.9-52.1) | 95.5 (94.2-96.5) |
|  | Namibia | 4 | 197 | 18.0-34.0 | 24.0 | 54.6 (28.1-79.9) | 92.8 (94.9-96.6) | 4 | 205 | 13.0-43.0 | 22.0 | 23.5 (10.1-40.1) | 84.7 (61.8-93.9) |
|  | Nigeria | 10 | 1,661 | 17.1-61.3 | 29.9 | 32.6 (18.9-47.9) | 96.5 (95.0-97.6) | 4 | 544 | 8.7-43.8 | 15.4 | 11.3 (8.6-14.4) | 71.4 (18.6-90.0) |
|  | Rwanda | 3 | 1,835 | 41.0-59.0 | 43.2 | 47.7 (37.7-57.7) | 92.9 (82.5-97.1) | - | - | - | - | - | - |
|  | Senegal | 8 | 888 | 7.7-27.1 | 18.4 | 16.2 (11.6-21.4) | 73.4 (45.8-86.9) | - | - | - | - | - | - |
|  | South Africa | 49 | 28,380 | 1.5-92.5 | 45.0 | 43.0 (34.1-52.2) | 99.6 (99.5-99.6) | 30 | 16,069 | 2.0-84.2 | 14.0 | 22.1 (14.5-30.6) | 99.3 (99.3-99.4) |
|  | Tanzania | 25 | 10,551 | 7.0-68.0 | 34.7 | 37.1 (31.7-42.7) | 96.7 (96.0-97.4) | 7 | 5,274 | 25.0-54.8 | 39.1 | 32.4 (28.0-37.0) | 96.2 (94.1-97.6) |
|  | Uganda | 29 | 22,622 | 15.3-90.7 | 64.0 | 61.5 (52.4-70.2) | 99.5 (99.4-99.5) | 27 | 22,519 | 9.9-81.9 | 46.5 | 41.2 (32.9-49.8) | 99.4 (99.3-99.5) |
|  | Zambia | 21 | 17,934 | 5.6-80.0 | 44.0 | 44.7 (32.7-57.1) | 99.6 (99.6-99.7) | 21 | 8,039 | 1.0-62.5 | 37.1 | 28.2 (18.5-39.1) | 99.1 (98.9-99.2) |
|  | Zimbabwe | 27 | 12,826 | 0.1-71.0 | 34.0 | 26.8 (16.1-39.0) | 99.5 (99.5-99.6) | 13 | 3,149 | 0.1-62.2 | 41.1 | 29.5 (13.5-48.7) | 99.6 (99.6-99.7) |
|  | Other countries^†^ | 5 | 717 | 23.0-97.4 | 65.9 | 68.6 (39.2-91.7) | 98.2 (97.3-98.8) | - | - | - | - | - | - |
| **African subregions** | Eastern Africa | 104 | 54,717 | 4.4-91.3 | 49.1 | 49.1 (44.3-53.9) | 99.2 (99.2-99.3) | 67 | 45,022 | 1.9-81.9 | 39.1 | 34.9 (26.7-40.3) | 99.3 (99.2-99.3) |
|  | Southern Africa | 134 | 66,895 | 0.1-92.5 | 44.2 | 41.6 (36.1-47.3) | 99.5 (99.5-99.6) | 90 | 33,223 | 0.0-84.5 | 25.3 | 28.4 (23.0-34.0) | 99.2 (99.1-99.2) |
|  | Western Africa | 43 | 10,942 | 6.9-97.4 | 27.1 | 30.2 (24.8-35.8) | 97.3 (96.9-97.7) | 21 | 4,657 | 1.0-43.8 | 12.5 | 13.6 (9.7-17.9) | 92.6 (90.1-94.5) |
|  | Central Africa | 9 | 1,480 | 14.0-78.0 | 68.0 | 58.8 (42.6-74.2) | 97.4 (96.4-98.2) | 8 | 996 | 3.0-59.0 | 34.5 | 31.5 (15.4-50.2) | 97.2 (96.0-98.1) |
| **Age group** | <20 years | 55 | 25,504 | 2.4-46.0 | 17.0 | 16.2 (13.8-18.9) | 96.4 (95.8-96.9) | 29 | 13,676 | 0.0-17.8 | 6.0 | 6.1 (4.5-79.7) | 93.3 (91.4-94.9) |
|  | 20-30 years | 63 | 30,832 | 7.7-91.3 | 43.3 | 44.8 (40.6-49.0) | 98.1 (97.9-98.3) | 47 | 18,194 | 2.9-57.7 | 20.7 | 20.3 (16.9-23.9) | 96.9 (96.4-97.3) |
|  | 30-40 years | 36 | 17,033 | 24.9-90.4 | 70.5 | 67.6 (62.8-72.3) | 97.6 (97.0-97.8) | 31 | 9,319 | 16.4-73.1 | 49.1 | 47.5 (42.5-52.6) | 95.4 (94.3-96.3) |
|  | 40-50 years | 24 | 7,592 | 20.0-92.5 | 71.9 | 67.0 (63.3-76.3) | 97.0 (96.3-97.6) | 22 | 4,844 | 37.0-84.2 | 55.0 | 56.9 (51.3-62.5) | 92.5 (89.9-94.4) |
|  | >50 years | 7 | 2,044 | 50.6-80.0 | 60.6 | 62.3 (52.6-71.6) | 94.1 (90.2-96.4) | 77 | 1,989 | 39.6-69.6 | 50.0 | 51.5 (39.7-63.2) | 96.1 (94.0-97.5) |
|  | Mixed | 105 | 51,029 | 0.1-97.4 | 41.8 | 41.5 (35.8-47.3) | 99.4 (99.4-99.4) | 50 | 35,876 | 0.1-81.8 | 30.6 | 29.5 (22.4-37.0) | 99.5 (99.5-99.6) |
| **Year of publication category** | ≤2005 | 61 | 10,907 | 5.7-88.0 | 50.0 | 50.5 (44.4-56.5) | 97.4 (97.1-97.7) | 51 | 9,337 | 1.0-76.0 | 37.0 | 30.5 (24.4-37.0) | 97.7 (97.3-98.0) |
|  | 2006-2015 | 168 | 79,377 | 0.1-97.4 | 41.0 | 40.3 (36.1-44.6) | 99.3 (99.3-99.3) | 100 | 57,869 | 0.0-81.9 | 35.2 | 30.3 (25.5-35.2) | 99.3 (99.3-99.4) |
|  | >2015 | 61 | 43,750 | 1.5-92.5 | 44.0 | 43.4 (35.7-51.2) | 99.6 (99.6-99.7) | 35 | 16,692 | 1.9-84.2 | 18.0 | 24.0 (17.1-31.7) | 99.1 (99.1-99.3) |

^*^ I^2^: a measure that assesses the magnitude of between-study variation that is due to actual differences in seroprevalence across studies rather than chance. All meta-analyses had a statistically significant Cochrane’s Q-statistic (p<0.001) and a wide prediction interval indicative of strong heterogeneity.

^†^ Other countries: Cote d’Ivoire, Eritrea, Gabon, Mali.

Abbreviations: CI = Confidence interval, HSV-2 = Herpes simplex virus type 2.

# **Figure S2.** Forest plots presenting the outcomes of the pooled mean herpes simplex virus type 2 (HSV-2) seroprevalence among the different at risk populations across the sub-Saharan Africa subregions.

##

Eastern Africa









 Abbreviations: HIV = Human immunodeficiency virus, HSV-2 = Herpes simplex virus type 2, STI = Sexually transmitted infection.

##

Southern Africa












 Abbreviations: HIV = Human immunodeficiency virus, HSV-2 = Herpes simplex virus type 2, STI = Sexually transmitted infection.

##

Western Africa



 Abbreviations: HIV = Human immunodeficiency virus, HSV-2 = Herpes simplex virus type 2, STI = Sexually transmitted infection.

##

Central Africa

Abbreviations: HIV = Human immunodeficiency virus, HSV-2 = Herpes simplex virus type 2, STI = Sexually transmitted infection.

##

Mixed regions in sub-Saharan Africa

Abbreviations: HIV = Human immunodeficiency virus, HSV-2 = Herpes simplex virus type 2, STI = Sexually transmitted infection.

# **Table S9.** Univariable and multivariable meta-regression analyses for herpes simplex virus type 2 seroprevalence among the different at risk populations in sub-Saharan Africa using the year of data collection as a categorical variable or as a linear term (in replacement of year of publication).

|  | | | **Outcome measures** | **Sample size** | **Univariable analysis** | | | | **Multivariable analysis** | | | |
| --- | --- | --- | --- | --- | --- | --- | --- | --- | --- | --- | --- | --- |
|  |  |  | **Total n** | **Total N** | ***RR* (95%CI)** | **p-value** | **LR test p-value** | **Adjusted R^2^ (%)** | **Model 1^*^** | | **Model 2**^†^ | |
|  |  |  |  |  |  |  |  |  | ***ARR* (95% CI)** | **p-value** | ***ARR* (95% CI)** | **p-value** |
| **Population characteristics** | **Population type** | General populations | 507 | 230,541 | 1.00 | - | <0.001 | 12.9 | 1.00 | - | 1.00 | - |
|  |  | Intermediate-risk populations | 45 | 9,259 | 1.73 (1.35-2.21) | <0.001 |  |  | 1.59 (1.31-1.92) | <0.001 | 1.53 (1.26-1.85) | <0.001 |
|  |  | Higher-risk populations | 40 | 13,476 | 1.70 (1.31-2.20) | <0.001 |  |  | 1.63 (1.33-2.01) | <0.001 | 1.61 (1.31-1.98) | <0.001 |
|  |  | HIV negative populations | 51 | 38,533 | 1.41 (1.12-1.78) | 0.003 |  |  | 1.32 (1.08-1.62) | 0.006 | 1.32 (1.08-1.61) | 0.006 |
|  |  | HIV positive individuals and individuals in HIV discordant couples | 42 | 15,521 | 2.18 (1.70-2.80) | <0.001 |  |  | 2.16 (1.77-2.64) | <0.001 | 2.22 (1.82-2.72) | <0.001 |
|  |  | STI clinic attendees and symptomatic populations | 72 | 11,996 | 1.88 (1.54-2.28) | <0.001 |  |  | 1.84 (1.56-2.17) | <0.001 | 1.83 (1.55-2.15) | <0.001 |
|  |  | Other populations | 16 | 6,506 | 1.53 (1.03-2.27) | 0.033 |  |  | 1.19 (0.88-1.61) | 0.241 | 1.23 (0.91-1.66) | 0.176 |
|  | **Age group** | <20 years | 108 | 42,984 | 1.00 | - | <0.001 | 32.6 | 1.00 | - | 1.00 | - |
|  |  | 20-30 years | 144 | 55,344 | 2.49 (2.07-3.00) | <0.001 |  |  | 2.49 (2.14-2.91) | <0.001 | 2.52 (2.17-2.94) | <0.001 |
|  |  | 30-40 years | 84 | 27,875 | 4.26 (3.46-5.24) | <0.001 |  |  | 4.38 (3.68-5.21) | <0.001 | 4.45 (3.74-5.30) | <0.001 |
|  |  | 40-50 years | 52 | 12,650 | 4.62 (3.64-5.86) | <0.001 |  |  | 5.15 (4.21-6.29) | <0.001 | 5.28 (4.32-6.45) | <0.001 |
|  |  | >50 years | 16 | 4,127 | 4.30 (2.96-6.24) | <0.001 |  |  | 4.62 (3.39-6.30) | <0.001 | 4.70 (3.45-6.41) | <0.001 |
|  |  | Mixed ages | 369 | 182,852 | 3.10 (2.64-3.64) | <0.001 |  |  | 2.39 (2.07-2.76) | <0.001 | 2.38 (2.06-2.75) | <0.001 |
|  | **Sex** | Women | 447 | 204,899 | 1.00 | - | <0.001 | 4.7 | 1.00 | - | 1.00 | - |
|  |  | Men | 269 | 100,000 | 0.68 (0.60-0.78) | <0.001 |  |  | 0.61 (0.56-0.68) | <0.001 | 0.61 (0.56-0.67) | <0.001 |
|  |  | Mixed sexes | 57 | 20,933 | 0.89 (0.70-1.12) | 0.337 |  |  | 0.79 (0.65-0.95) | 0.012 | 0.76 (0.63-0.93) | 0.008 |
|  | **African subregion** | Eastern Africa | 298 | 144,196 | 1.00 | - | <0.001 | 3.3 | 1.00 | - | 1.00 | - |
|  |  | Southern Africa | 319 | 128,395 | 0.82 (0.72-0.94) | 0.006 |  |  | 0.82 (0.73-0.93) | 0.002 | 0.85 (0.75-0.95) | 0.008 |
|  |  | Western Africa | 94 | 21,301 | 0.63 (0.52-0.77) | <0.001 |  |  | 0.60 (0.52-0.69) | <0.001 | 0.60 (0.51-0.69) | <0.001 |
|  |  | Central Africa | 48 | 5,832 | 1.01 (0.78-1.31) | 0.909 |  |  | 0.72 (0.58-0.89) | 0.003 | 0.77 (0.62-0.95) | 0.017 |
|  |  | Mixed regions | 14 | 26,225 | 1.23 (0.79-1.91) | 0.347 |  |  | 1.00 (0.47-2.15) | 0.982 | 1.01 (0.47-2.17) | 0.972 |
|  | **National income** | LIC | 326 | 123,156 | 1.00 | - | 0.027 | 0.7 | 1.00 | - | 1.00 | - |
|  |  | LMIC | 285 | 107,905 | 0.86 (0.75-0.98) | 0.030 |  |  | 0.98 (0.88-1.09) | 0.763 | 0.97 (0.87-1.08) | 0.665 |
|  |  | UMIC | 145 | 63,908 | 0.89 (0.75-1.05) | 0.195 |  |  | 1.16 (0.99-1.35) | 0.062 | 1.14 (0.97-1.33) | 0.100 |
|  |  | Mixed | 17 | 30,863 | 1.35 (0.90-2.03) | 0.141 |  |  | 0.85 (0.42-1.70) | 0.657 | 0.87 (0.43-1.74) | 0.701 |
| **Study methodology characteristics** | **Assay type** | Western Blot | 82 | 19,787 | 1.00 | - | 0.089 | 0.5 | 1.00 | - | 1.00 | - |
|  |  | ELISA | 681 | 304,639 | 0.86 (0.71-1.05) | 0.157 |  |  | 1.05 (0.90-1.23) | 0.510 | 1.02 (0.86-1.20) | 0.784 |
|  |  | Rapid test | 10 | 1,406 | 0.55 (0.31-0.97) | 0.041 |  |  | 0.75 (0.49-1.15) | 0.198 | 0.73 (0.48-1.12) | 0.160 |
|  | **Sample size**^‡^ | ≥100 | 740 | 324,163 | 1.00 | - | 0.634 | 0.0 | - | - | - | - |
|  |  | <100 | 33 | 1,669 | 1.07 (0.79-1.45) | 0.654 |  |  | - | - | - | - |
|  | **Sampling method** | Probability based | 311 | 165,963 | 1.00 | - | <0.001 | 3.6 | 1.00 | - | 1.00 | - |
|  |  | Non-probability based | 462 | 159,869 | 1.35 (1.20-1.53) | <0.001 |  |  | 1.05 (0.93-1.17) | 0.390 | 1.04 (0.92-1.16) | 0.493 |
|  | **Response rate** | ≥80% | 255 | 142,489 | 1.00 | - | <0.001 | 4.0 | 1.00 | - | 1.00 | - |
|  |  | <80% | 153 | 57,722 | 0.95 (0.80-1.12) | 0.564 |  |  | 1.17 (1.03-1.32) | 0.015 | 1.18 (1.04-1.34) | 0.007 |
|  |  | Unclear | 365 | 125,621 | 1.34 (1.17-1.53) | <0.001 |  |  | 1.27 (1.14-1.42) | <0.001 | 1.27 (1.14-1.42) | <0.001 |
| **Temporal variables** | **Year of data collection category**^§^ | <2000 | 239 | 47,629 | 1.00 | - | <0.001 | 3.4 | 1.00 | - | - | - |
|  |  | 2000-2010 | 393 | 206,142 | 0.82 (0.71-0.99) | 0.032 |  |  | 0.82 (0.73-0.92) | 0.001 | - | - |
|  |  | >2010 | 141 | 72,061 | 0.68 (0.56-0.80) | <0.001 |  |  | 0.79 (0.68-0.92) | 0.002 | - | - |
|  | **Year of data collection** | | 773 | 325,832 | 0.98 (0.97-0.99) | <0.001 | <0.001 | 3.1 | - | - | 0.98 (0.98-0.99) | 0.009 |

^*^ Variance explained by the multivariable model (adjusted *R^2^*) = 57.68%.

^†^ Variance explained by multivariable model 2 (adjusted *R^2^*) = 57.58%.

^‡^ Sample size denotes the sample size of each study population found in the original publication.

^§^ The categories were set based on the observed median time between the year of publication and year of data collection of 5 years.

Abbreviations: *ARR* = Adjusted risk ratio, CI = Confidence interval*,* ELISA = Enzyme-linked immunosorbent assay, HSV-2 = Herpes simplex virus type 2, HIV = human immunodeficiency virus, LIC = Low-income country, LMIC = Lower-middle-income country, LR = Likelihood ratio, *RR* = Risk ratio, STI = Sexually transmitted infection, UMIC = Upper-middle-income country.

# **Table S10.** Studies reporting proportions of HSV-2 virus isolation in clinically-diagnosed genital ulcer disease and in clinically-diagnosed genital herpes in sub-Saharan Africa.

| **Author, year** | **Year(s) of data collection** | **Country** | **Study site** | **Study design** | **Sampling method** | **Population** | **HSV-2 biological assay** | **Sample size** | **Proportion of HSV-2 detection (%)** |
| --- | --- | --- | --- | --- | --- | --- | --- | --- | --- |
| **Patients with clinically diagnosed GUD** | | |  |  |  |  |  |  |  |
| Ahmed, 2003^214^ | 1999 | Tanzania | Outpatient clinic | CS | Conv | Patients with GUD | PCR | 50 | 73.0 |
| Ahmed, 2003^214^ | 1999 | Tanzania | Outpatient clinic | CS | Conv | Patients with GUD | PCR | 52 | 42.0 |
| Chen, 2000^164^ | 1993-94 | South Africa | Outpatient clinic | CS | Conv | Men with GUD | PCR | 538 | 35.9 |
| Gray, 2009^215^ | 2002-06 | Uganda | Community | CS | Conv | Circumcised men with GUD | PCR | 25 | 48.0 |
| Gray, 2009^215^ | 2002-06 | Uganda | Community | CS | Conv | Uncircumcised men with GUD | PCR | 56 | 39.4 |
| Kamya, 1995^216^ | 1990-91 | Uganda | Outpatient clinic | CS | Conv | Patients with GUD | IF | 98 | 32.7 |
| Kularatne, 2018^167^ | 2007-15 | South Africa | Outpatient clinic | CS | Conv | Patients with GUD | PCR | 771 | 60.7 |
| LeGoff, 2007^213^ | 2003-05 | Ghana and Central African Republic | Outpatient clinic | CS | Conv | Women with GUD | PCR | 422 | 50.0 |
| Masaka, 2012^217^ | 2010 | Zambia | Outpatient clinic | CS | Conv | Patients with GUD | PCR | 200 | 28.0 |
| Mayaud, 2008^218^ | - | Burkina Faso | Community | CS | Conv | Women with GUD | PCR | 37 | 49.0 |
| Mungati, 2018^219^ | 2014-15 | Zimbabwe | Outpatient clinic | CS | Conv | Patients with GUD | PCR | 200 | 38.5 |
| Mwansasu, 2002^115^ | - | Tanzania | Community | CS | Conv | Patients with GUD | PCR | 70 | 64.3 |
| Nilsen, 2007^220^ | 1999-01 | Tanzania | Outpatient clinic | CS | Conv | Patients with GUD | PCR | 301 | 64.0 |
| O'Farrell, 2007^172^ | 2004 | South Africa | STI clinic | CS | Conv | Males with GUD | PCR | 162 | 52.7 |
| Oni, 1997^221^ | 1992 | Nigeria | Outpatient clinic | CS | Conv | Males attending an STI clinic | CF and IF | 24 | 8.3 |
| Paz-Bailey, 2005^222^ | 2001-02 | Botswana | Outpatient clinic | CS | Conv | Patients with GUD | PCR | 137 | 58.0 |
| Paz-Bailey, 2009^173^ | 2005-06 | South Africa | Outpatient clinic | CS | RS | Men with GUD taking acyclovir | PCR | 309 | 69.9 |
| Paz-Bailey, 2009^173^ | 2005-06 | South Africa | Outpatient clinic | CS | RS | Men with GUD taking a placebo | PCR | 306 | 72.2 |
| Phiri, 2013^174^ | 2004-06 | Malawi | Outpatient clinic | CS | Conv | Males and Females with GUD | PCR | 398 | 67.0 |
| Pickering, 2005^223^ | 1999-01 | Uganda | Outpatient clinic | CS | Conv | Adult males and females presenting with GUD | PCR | 202 | 47.0 |
| Suntoke, 2009^116^ | 2002-06 | Uganda | Community | CS | Conv | >19 years old patients with GUD | PCR | 100 | 62.0 |
| Tanton, 2010^224^ | 2004-06 | Tanzania | Community | CS | Conv | Women working in recreational facilities | PCR | 22 | 18.2 |
| Vandenhoudt, 2013^104^ | 2008 | Kenya | Community | CS | RDS | FSWs with GUD | PCR | 22 | 40.9 |
| Vandepitte, 2011^105^ | 2008-09 | Uganda | Community | CS | Conv | FSWs with GUD | PCR | 62 | 35.0 |
| Zimba, 2011^175^ | 2005 | Mozambique | Outpatient clinic | CS | Conv | Patients with GUD | PCR | 76 | 62.0 |
| **Patients with clinically diagnosed genital herpes^*^** | | | | | | | | | |
| Chen, 2000^164^ | 1993-94 | South Africa | Outpatient clinic | CS | Conv | Men with genital herpes | PCR | 193 | 100.0 |
| Kamya, 1995^216^ | 1990-91 | Uganda | Outpatient clinic | CS | Conv | Males and females with genital herpes | IF | 35 | 91.4 |
| Kularatne, 2018^167^ | 2007-15 | South Africa | Outpatient clinic | CS | Conv | Patients with genital herpes | PCR | 468 | 100.0 |
| Lai, 2003^225^ | 1998 | South Africa | Outpatient clinic | CS | Conv | Genital herpes swabs collected in 1998 | PCR | 57 | 100.0 |
| Lai, 2003^225^ | 1993-94 | South Africa | Outpatient clinic | CS | Conv | Genital herpes swabs collected in 1993 | PCR | 14 | 100.0 |
| Morse, 1997^171^ | 1993-94 | Lesotho | Outpatient clinic | CS | Conv | Patients with genital herpes | PCR | 20 | 95.0 |
| Mungati, 2018^219^ | 2014-15 | Zimbabwe | Outpatient clinic | CS | Conv | Patients with genital herpes | PCR | 77 | 98.7 |
| Paz-Bailey, 2009^173^ | 2005-06 | South Africa | Outpatient clinic | CS | RS | Men with genital herpes | PCR | 451 | 53.0 |
| Suntoke, 2009^116^ | 2002-06 | Uganda | Community | CS | Conv | Patients with GUD testing positive for HSV | PCR | 65 | 95.4 |

^*^ Included studies did not distinguish between primary and recurrent genital herpes cases.

Abbreviations: CF = Complement fixation, Conv = Convenience, CS = Cross sectional, FSWs = Female sex workers, GUD = Genital ulcer disease, HSV-2 = Herpes simplex virus type 2, IF = Immunofluorescence, PCR = Polymerase chain reaction, RCT = Randomized controlled trial, RS = Random sampling, STI = Sexually transmitted infections.

# **Table S11.** Summary of the precision assessment and risk of bias assessment for the studies reporting HSV-2 seroprevalence in sub-Saharan Africa.

| **Quality assessment** | **HSV-2 seroprevalence measures** | |
| --- | --- | --- |
|  | **Number of studies** | **%** |
| **Precision of seroprevalence measures^a^** | | |
| Low precision | 34 | 10.6 |
| High precision | 288 | 89.4 |
| **Risk of bias quality domain^b^** | | |
| **Sampling method** | | |
| Low risk of bias | 88 | 27.3 |
| High risk of bias | 234 | 72.7 |
| **Response rate** | | |
| Low risk of bias | 85 | 26.4 |
| High risk of bias | 45 | 14.0 |
| Unclear risk of bias | 192 | 59.6 |
| **Summary of the risk of bias assessment** | | |
| **Low risk of bias** |  |  |
| In at least one quality domain | 107 | 33.2 |
| In both quality domains | 39 | 12.1 |
| **High risk of bias** |  |  |
| In at least one quality domain | 91 | 28.3 |
| In both quality domains | 23 | 7.1 |
| **Seroprevalence studies where risk of bias assessment was possible** | **322** | **100** |

^a^ Precision was assessed based on the overall sample size (not each stratum subsample size) of the study as reported in the record/publication.

^b^ Risk of bias was assessed based on the overall sample size (not each stratum subsample size) of the study as reported in the record/publication.

Abbreviations: HSV-2 = Herpes simplex virus type 2.

# **References**

1. West Africa Brief (Actualite Ouest-Africaine). The six regions of the African Union. 2017. <http://www.west-africa-brief.org/content/en/six-regions-african-union>, Accessed on June, 2019.

2. Smolak A, Chemaitelly H, Hermez JG, Low N, Abu-Raddad LJ. Epidemiology of Chlamydia trachomatis in the Middle East and north Africa: a systematic review, meta-analysis, and meta-regression. *The Lancet Global health* 2019; **7**(9): e1197-e225.

3. Abu-Raddad LJ, Akala FA, Semini I, Riedner G, Wilson D, Tawil O. Characterizing the HIV/AIDS Epidemic in the Middle East and North Africa : Time for Strategic Action. World Bank. © World Bank. <https://openknowledge.worldbank.org/handle/10986/2457> License: CC BY 3.0 IGO. 2010.

4. Brunham RC, Plummer FA. A general model of sexually transmitted disease epidemiology and its implications for control. *Med Clin North Am* 1990; **74**(6): 1339-52.

5. Low N, Broutet N, Adu-Sarkodie Y, Barton P, Hossain M, Hawkes S. Global control of sexually transmitted infections. *Lancet (London, England)* 2006; **368**(9551): 2001-16.

6. World Health Organization. Global strategy for the prevention and control of sexually transmitted infections: 2006 - 2015. Breaking the chain of transmission. WHO Press, Geneva, Switzerland. Found at: <http://www.who.int/reproductivehealth/publications/rtis/9789241563475/en/>. Last accessed April 2012. 2007.

7. World Bank. World Bank Country and Lending Groups (Available at: <https://datahelpdesk.worldbank.org/knowledgebase/articles/906519-world-bank-country-and-lending-groups>. Accessed in June 2017). 2017.

8. Abdool Karim Q, Kharsany AB, Leask K, et al. Prevalence of HIV, HSV-2 and pregnancy among high school students in rural KwaZulu-Natal, South Africa: a bio-behavioural cross-sectional survey. *Sexually transmitted infections* 2014; **90**(8): 620-6.

9. Akinyi B, Odhiambo C, Otieno F, et al. Prevalence, incidence and correlates of HSV-2 infection in an HIV incidence adolescent and adult cohort study in western Kenya. *PloS one* 2017; **12**(6): e0178907.

10. Biraro S, Kamali A, White R, et al. Effect of HSV-2 on population-level trends in HIV incidence in Uganda between 1990 and 2007. *Tropical medicine & international health : TM & IH* 2013; **18**(10): 1257-66.

11. De Baetselier I, Menten J, Cuylaerts V, et al. Prevalence and incidence estimation of HSV-2 by two IgG ELISA methods among South African women at high risk of HIV. *PloS one* 2015; **10**(3): e0120207.

12. del Mar Pujades Rodriguez M, Obasi A, Mosha F, et al. Herpes simplex virus type 2 infection increases HIV incidence: a prospective study in rural Tanzania. *AIDS (London, England)* 2002; **16**(3): 451-62.

13. Hallfors DD, Cho H, Hartman S, Mbai I, Ouma CA, Halpern CT. Process Evaluation of a Clinical Trial to Test School Support as HIV Prevention Among Orphaned Adolescents in Western Kenya. *Prevention science : the official journal of the Society for Prevention Research* 2017; **18**(8): 955-63.

14. Heffron R, Chao A, Mwinga A, et al. High prevalent and incident HIV-1 and herpes simplex virus 2 infection among male migrant and non-migrant sugar farm workers in Zambia. *Sexually transmitted infections* 2011; **87**(4): 283-8.

15. Jewkes R, Nduna M, Levin J, et al. Impact of stepping stones on incidence of HIV and HSV-2 and sexual behaviour in rural South Africa: cluster randomised controlled trial. *BMJ (Clinical research ed)* 2008; **337**: a506.

16. Kamali A, Nunn AJ, Mulder DW, Van Dyck E, Dobbins JG, Whitworth JA. Seroprevalence and incidence of genital ulcer infections in a rural Ugandan population. *Sexually transmitted infections* 1999; **75**(2): 98-102.

17. Kamali A, Quigley M, Nakiyingi J, et al. Syndromic management of sexually-transmitted infections and behaviour change interventions on transmission of HIV-1 in rural Uganda: a community randomised trial. *Lancet (London, England)* 2003; **361**(9358): 645-52.

18. Kebede Y, Dorigo-Zetsma W, Mengistu Y, et al. Transmission of herpes simplex virus Type 2 among factory workers in Ethiopia. *The Journal of infectious diseases* 2004; **190**(2): 365-72.

19. McFarland W, Gwanzura L, Bassett MT, et al. Prevalence and incidence of herpes simplex virus type 2 infection among male Zimbabwean factory workers. *The Journal of infectious diseases* 1999; **180**(5): 1459-65.

20. Mensch BS, Grant MJ, Soler-Hampejsek E, Kelly CA, Chalasani S, Hewett PC. Does schooling protect sexual health? The association between three measures of education and STIs among adolescents in Malawi. *Population studies* 2020; **74**(2): 241-61.

21. Munjoma MW, Kurewa EN, Mapingure MP, et al. The prevalence, incidence and risk factors of herpes simplex virus type 2 infection among pregnant Zimbabwean women followed up nine months after childbirth. *BMC women's health* 2010; **10**: 2.

22. Nakubulwa S, Kaye DK, Bwanga F, Tumwesigye NM, Nakku-Joloba E, Mirembe FM. Incidence and risk factors for herpes simplex virus type 2 seroconversion among pregnant women in Uganda: A prospective study. *Journal of infection in developing countries* 2016; **10**(10): 1108-15.

23. Pettifor A, MacPhail C, Hughes JP, et al. The effect of a conditional cash transfer on HIV incidence in young women in rural South Africa (HPTN 068): a phase 3, randomised controlled trial. *The Lancet Global health* 2016; **4**(12): e978-e88.

24. Radebe F, Jemmott J, Klopper A, et al. Incidence and prevalence of sexually transmitted infections among school students in the eastern cape, South Africa. *Sexually transmitted infections* 2011; **87**(SUPPL. 1): A30-A1.

25. Rosenberg M, Pettifor A, Duta M, et al. Executive function associated with sexual risk in young South African women: Findings from the HPTN 068 cohort. *PloS one* 2018; **13**(4): e0195217.

26. Sobngwi-Tambekou J, Taljaard D, Lissouba P, et al. Effect of HSV-2 serostatus on acquisition of HIV by young men: results of a longitudinal study in Orange Farm, South Africa. *The Journal of infectious diseases* 2009; **199**(7): 958-64.

27. Stoner MCD, Edwards JK, Miller WC, et al. Does partner selection mediate the relationship between school attendance and HIV/Herpes simplex virus-2 among adolescent girls and young women in South Africa: An analysis of HIV prevention trials network 068 data. *Journal of Acquired Immune Deficiency Syndromes* 2018; **79**(1): 20-7.

28. Tobian AA, Charvat B, Ssempijja V, et al. Factors associated with the prevalence and incidence of herpes simplex virus type 2 infection among men in Rakai, Uganda. *The Journal of infectious diseases* 2009; **199**(7): 945-9.

29. Tobian AA, Kigozi G, Redd AD, et al. Male circumcision and herpes simplex virus type 2 infection in female partners: a randomized trial in Rakai, Uganda. *The Journal of infectious diseases* 2012; **205**(3): 486-90.

30. van de Wijgert JH, Morrison CS, Brown J, et al. Disentangling contributions of reproductive tract infections to HIV acquisition in African Women. *Sexually transmitted diseases* 2009; **36**(6): 357-64.

31. Wagner HU, Van Dyck E, Roggen E, et al. Seroprevalence and incidence of sexually transmitted diseases in a rural Ugandan population. *International journal of STD & AIDS* 1994; **5**(5): 332-7.

32. Kapiga SH, Sam NE, Shao JF, et al. Herpes simplex virus type 2 infection among bar and hotel workers in northern Tanzania: prevalence and risk factors. *Sexually transmitted diseases* 2003; **30**(3): 187-92.

33. Meque I, Dube K, Feldblum PJ, et al. Prevalence, incidence and determinants of herpes simplex virus type 2 infection among HIV-seronegative women at high-risk of HIV infection: a prospective study in Beira, Mozambique. *PloS one* 2014; **9**(2): e89705.

34. Ondondo RO, Ng'ang'a ZW, Mpoke S, Kiptoo M, Bukusi E. Incidence and prevalence of herpes simplex virus type-2 infections among fishermen in Kisumu, Kenya. *International Journal of Infectious Diseases* 2014; **21**(SUPPL. 1): 422.

35. Riedner G, Hoffmann O, Rusizoka M, et al. Decline in sexually transmitted infection prevalence and HIV incidence in female barworkers attending prevention and care services in Mbeya Region, Tanzania. *AIDS (London, England)* 2006; **20**(4): 609-15.

36. Tassiopoulos KK, Seage G, 3rd, Sam N, et al. Predictors of herpes simplex virus type 2 prevalence and incidence among bar and hotel workers in Moshi, Tanzania. *The Journal of infectious diseases* 2007; **195**(4): 493-501.

37. Braunstein SL, Ingabire CM, Kestelyn E, et al. High human immunodeficiency virus incidence in a cohort of Rwandan female sex workers. *Sexually transmitted diseases* 2011b; **38**(5): 385-94.

38. Chohan V, Baeten JM, Benki S, et al. A prospective study of risk factors for herpes simplex virus type 2 acquisition among high-risk HIV-1 seronegative women in Kenya. *Sexually transmitted infections* 2009; **85**(7): 489-92.

39. Kaul R, Nagelkerke NJ, Kimani J, et al. Prevalent herpes simplex virus type 2 infection is associated with altered vaginal flora and an increased susceptibility to multiple sexually transmitted infections. *The Journal of infectious diseases* 2007; **196**(11): 1692-7.

40. Masese L, Baeten JM, Richardson BA, et al. Incident herpes simplex virus type 2 infection increases the risk of subsequent episodes of bacterial vaginosis. *The Journal of infectious diseases* 2014; **209**(7): 1023-7.

41. Ramjee G, Williams B, Gouws E, Van Dyck E, De Deken B, Karim SA. The impact of incident and prevalent herpes simplex virus-2 infection on the incidence of HIV-1 infection among commercial sex workers in South Africa. *Journal of acquired immune deficiency syndromes (1999)* 2005; **39**(3): 333-9.

42. Traore IT, Hema MN, Meda N, et al. Effect of a tailored intervention package on HIV-1 acquisition among young female sex workers in ouagadougou, Burkina Faso. *Sexually transmitted infections* 2013; **89**(SUPPL. 1).

43. de Bruyn G, Shiboski S, van der Straten A, et al. The effect of the vaginal diaphragm and lubricant gel on acquisition of HSV-2. *Sexually transmitted infections* 2011; **87**(4): 301-5.

44. McCormack S, Ramjee G, Kamali A, et al. PRO2000 vaginal gel for prevention of HIV-1 infection (Microbicides Development Programme 301): a phase 3, randomised, double-blind, parallel-group trial. *Lancet (London, England)* 2010; **376**(9749): 1329-37.

45. Mehta SD, Moses S, Parker CB, Agot K, Maclean I, Bailey RC. Circumcision status and incident herpes simplex virus type 2 infection, genital ulcer disease, and HIV infection. *AIDS (London, England)* 2012; **26**(9): 1141-9.

46. Mehta SD, Moses S, Agot K, et al. Medical male circumcision and herpes simplex virus 2 acquisition: posttrial surveillance in Kisumu, Kenya. *The Journal of infectious diseases* 2013; **208**(11): 1869-76.

47. Mlisana K, Naicker N, Werner L, et al. Symptomatic vaginal discharge is a poor predictor of sexually transmitted infections and genital tract inflammation in high-risk women in South Africa. *Journal of Infectious Diseases* 2012; **206**(1): 6-14.

48. Perti T, Nyati M, Gray G, et al. Frequent genital HSV-2 shedding among women during labor in Soweto, South Africa. *Infectious diseases in obstetrics and gynecology* 2014; **2014**: 258291.

49. Celum C, Morrow RA, Donnell D, et al. Daily oral tenofovir and emtricitabine-tenofovir preexposure prophylaxis reduces herpes simplex virus type 2 acquisition among heterosexual HIV-1-uninfected men and women: a subgroup analysis of a randomized trial. *Annals of internal medicine* 2014; **161**(1): 11-9.

50. Cowan FM, Humphrey JH, Ntozini R, Mutasa K, Morrow R, Iliff P. Maternal Herpes simplex virus type 2 infection, syphilis and risk of intra-partum transmission of HIV-1: results of a case control study. *AIDS (London, England)* 2008a; **22**(2): 193-201.

51. Muiru AN, Guthrie BL, Bosire R, et al. Incident HSV-2 infections are common among HIV-1-discordant couples. *The Journal of infectious diseases* 2013; **208**(7): 1093-101.

52. Okuku HS, Sanders EJ, Nyiro J, et al. Factors associated with herpes simplex virus type 2 incidence in a cohort of human immunodeficiency virus type 1-seronegative Kenyan men and women reporting high-risk sexual behavior. *Sexually transmitted diseases* 2011; **38**(9): 837-44.

53. Amornkul PN, Vandenhoudt H, Nasokho P, et al. HIV prevalence and associated risk factors among individuals aged 13-34 years in Rural Western Kenya. *PloS one* 2009; **4**(7): e6470.

54. Anjulo AA, Abebe T, Hailemichael F, Mihret A. Seroprevalence and risk factors of herpes simplex virus-2 among pregnant women attending antenatal care at health facilities in Wolaita zone, Ethiopia. *Virology journal* 2016; **13**: 43.

55. Behling J, Chan AK, Zeh C, Nekesa C, Heinzerling L. Evaluating HIV Prevention Programs: Herpes Simplex Virus Type 2 Antibodies as Biomarker for Sexual Risk Behavior in Young Adults in Resource-Poor Countries. *PloS one* 2015; **10**(5): e0128370.

56. Braunstein SL, Ingabire CM, Geubbels E, et al. High burden of prevalent and recently acquired HIV among female sex workers and female HIV voluntary testing center clients in Kigali, Rwanda. *PloS one* 2011a; **6**(9): e24321.

57. De Walque D, Dow WH, Nathan R, et al. Incentivising safe sex: A randomised trial of conditional cash transfers for HIV and sexually transmitted infection prevention in rural Tanzania. *BMJ Open* 2012; **2**(1): 000747.

58. Dhont N, van de Wijgert J, Luchters S, Muvunyi C, Vyankandondera J, Temmerman M. Sexual violence, HSV-2 and HIV are important predictors for infertility in Rwanda. *Human reproduction (Oxford, England)* 2010; **25**(10): 2507-15.

59. Doyle AM, Ross DA, Maganja K, et al. Long-term biological and behavioural impact of an adolescent sexual health intervention in Tanzania: follow-up survey of the community-based MEMA kwa Vijana Trial. *PLoS medicine* 2010; **7**(6): e1000287.

60. Duflo E, Dupas P, Kremer M. Education, HIV, and Early Fertility: Experimental Evidence from Kenya. *The American economic review* 2015; **105**(9): 2757-97.

61. Ghebrekidan H, Ruden U, Cox S, Wahren B, Grandien M. Prevalence of herpes simplex virus types 1 and 2, cytomegalovirus, and varicella-zoster virus infections in Eritrea. *Journal of clinical virology : the official publication of the Pan American Society for Clinical Virology* 1999; **12**(1): 53-64.

62. Ghebremichael M, Paintsil E. High risk behaviors and sexually transmitted infections among men in Tanzania. *AIDS and behavior* 2011; **15**(5): 1026-32.

63. Ghebremichael M, Habtzgi D, Paintsil E. Deciphering the epidemic synergy of herpes simplex virus type 2 (HSV-2) on human immunodeficiency virus type 1 (HIV-1) infection among women in sub-Saharan Africa. *BMC research notes* 2012; **5**: 451.

64. Gorander S, Mbwana J, Lyamuya E, Lagergard T, Liljeqvist JA. Mature glycoprotein g presents high performance in diagnosing herpes simplex virus type 2 infection in sera of different tanzanian cohorts. *Clinical and vaccine immunology : CVI* 2006; **13**(6): 633-9.

65. Guwatudde D, Wabwire-Mangen F, Eller LA, et al. Relatively low HIV infection rates in rural Uganda, but with high potential for a rise: a cohort study in Kayunga District, Uganda. *PloS one* 2009; **4**(1): e4145.

66. Hallfors DD, Cho H, Mbai, II, et al. Disclosure of HSV-2 serological test results in the context of an adolescent HIV prevention trial in Kenya. *Sexually transmitted infections* 2015; **91**(6): 395-400.

67. Hokororo A, Kihunrwa A, Hoekstra P, et al. High prevalence of sexually transmitted infections in pregnant adolescent girls in Tanzania: a multi-community cross-sectional study. *Sexually transmitted infections* 2015; **91**(7): 473-8.

68. Holt BY, Effler P, Brady W, et al. Planning STI/HIV prevention among refugees and mobile populations: situation assessment of Sudanese refugees. *Disasters* 2003; **27**(1): 1-15.

69. Jespers V, Crucitti T, Menten J, et al. Prevalence and correlates of bacterial vaginosis in different sub-populations of women in sub-Saharan Africa: a cross-sectional study. *PloS one* 2014; **9**(10): e109670.

70. Kamali A, Kinsman J, Nalweyiso N, et al. A community randomized controlled trial to investigate impact of improved STD management and behavioural interventions on HIV incidence in rural Masaka, Uganda: trial design, methods and baseline findings. *Tropical medicine & international health : TM & IH* 2002; **7**(12): 1053-63.

71. Kapiga SH, Ewings FM, Ao T, et al. The epidemiology of HIV and HSV-2 infections among women participating in microbicide and vaccine feasibility studies in Northern Tanzania. *PloS one* 2013; **8**(7): e68825.

72. Kasubi MJ, Nilsen A, Marsden HS, Bergstrom T, Langeland N, Haarr L. Prevalence of antibodies against herpes simplex virus types 1 and 2 in children and young people in an urban region in Tanzania. *Journal of clinical microbiology* 2006; **44**(8): 2801-7.

73. Kuteesa MO, Weiss HA, Cook S, et al. Epidemiology of alcohol misuse and illicit drug use among young people aged 15-24 years in fishing communities in Uganda. *International Journal of Environmental Research and Public Health* 2020; **17 (7) (no pagination)**(2401).

74. Mehta SD, Nordgren RK, Agingu W, et al. Sexual Quality of Life and Association With HIV and Sexually Transmitted Infections Among a Cohort of Heterosexual Couples in Kenya. *Journal of Sexual Medicine* 2018; **15**(10): 1446-55.

75. MOH Uganda, ORC Macro. Uganda HIV/AIDS sero-behavioural survey: 2004-2005: Calverton, Maryland, USA: Ministry of Health and ORC Macro., 2006.

76. Msuya SE, Mbizvo E, Stray-Pedersen B, Sundby J, Sam NE, Hussain A. Reproductive tract infections and the risk of HIV among women in Moshi, Tanzania. *Acta obstetricia et gynecologica Scandinavica* 2002; **81**(9): 886-93.

77. Msuya SE, Mbizvo EM, Stray-Pedersen B, et al. Decline in HIV prevalence among women of childbearing age in Moshi urban, Tanzania. *International journal of STD & AIDS* 2007; **18**(10): 680-7.

78. Msuya SE, Uriyo J, Hussain A, et al. Prevalence of sexually transmitted infections among pregnant women with known HIV status in northern Tanzania. *Reproductive Health* 2009; **6**(1): 4.

79. Nakku-Joloba E, Kambugu F, Wasubire J, et al. Sero-prevalence of herpes simplex type 2 virus (HSV-2) and HIV infection in Kampala, Uganda. *African health sciences* 2014; **14**(4): 782-9.

80. Nakubulwa S, Kaye DK, Bwanga F, Tumwesigye NM, Mirembe FM. Genital infections and risk of premature rupture of membranes in Mulago Hospital, Uganda: a case control study. *BMC research notes* 2015; **8**: 573.

81. NASCOP (National AIDS/STI Control Program ). 2007 Kenya AIDS Indicator Survey: Final Report. Nairobe, Kenya, September 2009.

82. Nilsen A, Mwakagile D, Marsden H, Langeland N, Matre R, Haarr L. Prevalence of, and risk factors for, HSV-2 antibodies in sexually transmitted disease patients, healthy pregnant females, blood donors and medical students in Tanzania and Norway. *Epidemiology and infection* 2005; **133**(5): 915-25.

83. Norris AH, Kitali AJ, Worby E. Alcohol and transactional sex: how risky is the mix? *Social science & medicine (1982)* 2009; **69**(8): 1167-76.

84. Nyiro JU, Sanders EJ, Ngetsa C, et al. Seroprevalence, predictors and estimated incidence of maternal and neonatal Herpes Simplex Virus type 2 infection in semi-urban women in Kilifi, Kenya. *BMC infectious diseases* 2011; **11**: 155.

85. Oliver VO, Otieno G, Gvetadze R, et al. High prevalence of sexually transmitted infections among women screened for a contraceptive intravaginal ring study, Kisumu, Kenya, 2014. *International Journal of STD and AIDS* 2018; **29**(14): 1390-9.

86. Otieno FO, Ndivo R, Oswago S, et al. Correlates of prevalent sexually transmitted infections among participants screened for an HIV incidence cohort study in Kisumu, Kenya. *International journal of STD & AIDS* 2015; **26**(4): 225-37.

87. Reynolds SJ, Makumbi F, Newell K, et al. Effect of daily aciclovir on HIV disease progression in individuals in Rakai, Uganda, co-infected with HIV-1 and herpes simplex virus type 2: a randomised, double-blind placebo-controlled trial. *The Lancet Infectious diseases* 2012; **12**(6): 441-8.

88. Sivapalasingam S, McClelland RS, Ravel J, et al. An effective intervention to reduce intravaginal practices among HIV-1 uninfected Kenyan women. *AIDS Research and Human Retroviruses* 2014; **30**(11): 1046-54.

89. Tedla Y, Shibre T, Ali O, et al. Serum antibodies to Toxoplasma gondii and Herpesvidae family viruses in individuals with schizophrenia and bipolar disorder: a case-control study. *Ethiopian medical journal* 2011; **49**(3): 211-20.

90. Todd J, Grosskurth H, Changalucha J, et al. Risk factors influencing HIV infection incidence in a rural African population: a nested case-control study. *The Journal of infectious diseases* 2006; **193**(3): 458-66.

91. Weiss HA, Buve A, Robinson NJ, et al. The epidemiology of HSV-2 infection and its association with HIV infection in four urban African populations. *AIDS (London, England)* 2001; **15 Suppl 4**: S97-108.

92. Winston SE, Chirchir AK, Muthoni LN, et al. Prevalence of sexually transmitted infections including HIV in street-connected adolescents in western Kenya. *Sexually transmitted infections* 2015; **91**(5): 353-9.

93. Yahya-Malima KI, Evjen-Olsen B, Matee MI, Fylkesnes K, Haarr L. HIV-1, HSV-2 and syphilis among pregnant women in a rural area of Tanzania: prevalence and risk factors. *BMC infectious diseases* 2008; **8**: 75.

94. Yegorov S, Galiwango RM, Good SV, et al. Schistosoma mansoni infection and socio-behavioural predictors of HIV risk: a cross-sectional study in women from Uganda. *BMC infectious diseases* 2018; **18**(1): 586.

95. Ng'ayo OM, Bukusi E, R AM, et al. Sexual and demographic determinants for herpes simplex virus type 2 among fishermen along Lake Victoria, Kenya. *Sexually transmitted infections* 2008; **84**(2): 140-2.

96. Rakwar J, Jackson D, Maclean I, et al. Antibody to Haemophilus ducreyi among trucking company workers in Kenya. *Sexually transmitted diseases* 1997; **24**(5): 267-71.

97. Riedner G, Todd J, Rusizoka M, et al. Possible reasons for an increase in the proportion of genital ulcers due to herpes simplex virus from a cohort of female bar workers in Tanzania. *Sexually transmitted infections* 2007; **83**(2): 91-6.

98. Vallely A, Kasindi S, Hambleton IR, et al. Microbicides development program, Tanzania-baseline characteristics of an occupational cohort and reattendance at 3 months. *Sexually transmitted diseases* 2007; **34**(9): 638-43.

99. Watson-Jones D, Weiss HA, Rusizoka M, et al. Risk factors for herpes simplex virus type 2 and HIV among women at high risk in northwestern Tanzania: preparing for an HSV-2 intervention trial. *Journal of acquired immune deficiency syndromes (1999)* 2007; **46**(5): 631-42.

100. Baeten JM, Benki S, Chohan V, et al. Hormonal contraceptive use, herpes simplex virus infection, and risk of HIV-1 acquisition among Kenyan women. *AIDS (London, England)* 2007; **21**(13): 1771-7.

101. Baltzer H, Chege D, Rebbapragada A, et al. Relative HIV resistance in kenyan sex workers is not due to an altered prevalence or mucosal immune impact of herpes simplex virus type 2 infection. *Current HIV research* 2009; **7**(5): 504-7.

102. Masese L, Baeten JM, Richardson BA, et al. Changes in the contribution of genital tract infections to HIV acquisition among Kenyan high-risk women from 1993 to 2012. *AIDS (London, England)* 2015; **29**(9): 1077-85.

103. Priddy FH, Wakasiaka S, Hoang TD, et al. Anal sex, vaginal practices, and HIV incidence in female sex workers in urban Kenya: implications for the development of intravaginal HIV prevention methods. *AIDS Res Hum Retroviruses* 2011; **27**(10): 1067-72.

104. Vandenhoudt HM, Langat L, Menten J, et al. Prevalence of HIV and other sexually transmitted infections among female sex workers in Kisumu, Western Kenya, 1997 and 2008. *PloS one* 2013; **8**(1): e54953.

105. Vandepitte J, Bukenya J, Weiss HA, et al. HIV and other sexually transmitted infections in a cohort of women involved in high-risk sexual behavior in Kampala, Uganda. *Sexually transmitted diseases* 2011; **38**(4): 316-23.

106. Mehta SD, Moses S, Agot K, et al. Herpes simplex virus type 2 infection among young uncircumcised men in Kisumu, Kenya. *Sexually transmitted infections* 2008; **84**(1): 42-8.

107. Nakubulwa S, Mirembe FM, Kaye DK, Kaddu-Mulindwa D. Association between HSV-2 and HIV serostatus in pregnant women of known HIV serostatus attending Mulago hospital antenatal clinic, Kampala, Uganda. *Journal of infection in developing countries* 2009; **3**(10): 803-6.

108. Serwadda D, Gray RH, Sewankambo NK, et al. Human immunodeficiency virus acquisition associated with genital ulcer disease and herpes simplex virus type 2 infection: a nested case-control study in Rakai, Uganda. *The Journal of infectious diseases* 2003; **188**(10): 1492-7.

109. Baeten JM, McClelland RS, Corey L, et al. Vitamin A supplementation and genital shedding of herpes simplex virus among HIV-1-infected women: a randomized clinical trial. *The Journal of infectious diseases* 2004; **189**(8): 1466-71.

110. Madebe R, Kiwelu I, Ndaro A, et al. Herpes Simplex virus type 2 seroprevalence and risk factors among adolescents and youth with HIV-1 in Northern, Tanzania. *Journal of infection in developing countries* 2020; **14**(4): 398-403.

111. McClelland RS, Wang CC, Overbaugh J, et al. Association between cervical shedding of herpes simplex virus and HIV-1. *AIDS (London, England)* 2002; **16**(18): 2425-30.

112. Roxby AC, Drake AL, John-Stewart G, et al. Herpes simplex virus type 2, genital ulcers and HIV-1 disease progression in postpartum women. *PloS one* 2011; **6**(5): e19947.

113. Langeland N, Haarr L, Mhalu F. Prevalence of HSV-2 antibodies among STD clinic patients in Tanzania. *International journal of STD & AIDS* 1998; **9**(2): 104-7.

114. Mostad SB, Kreiss JK, Ryncarz AJ, et al. Cervical shedding of herpes simplex virus in human immunodeficiency virus-infected women: effects of hormonal contraception, pregnancy, and vitamin A deficiency. *The Journal of infectious diseases* 2000; **181**(1): 58-63.

115. Mwansasu A, Mwakagile D, Haarr L, Langeland N. Detection of HSV-2 in genital ulcers from STD patients in Dar es Salaam, Tanzania. *Journal of clinical virology : the official publication of the Pan American Society for Clinical Virology* 2002; **24**(3): 183-92.

116. Suntoke TR, Hardick A, Tobian AA, et al. Evaluation of multiplex real-time PCR for detection of Haemophilus ducreyi, Treponema pallidum, herpes simplex virus type 1 and 2 in the diagnosis of genital ulcer disease in the Rakai District, Uganda. *Sexually transmitted infections* 2009; **85**(2): 97-101.

117. Kassa D, Gebremichael G, Tilahun T, et al. Prevalence of sexually transmitted infections (HIV, hepatitis B virus, herpes simplex virus type 2, and syphilis) in pregnant women in Ethiopia: Trends over 10 years (2005-2014). *International Journal of Infectious Diseases* 2019; **79**: 50-7.

118. Abbai NS, Govender S, Nyirenda M. Herpes simplex virus-2 infections in pregnant women from Durban, South Africa: prevalence, risk factors and co-infection with HIV-1. *Southern African Journal of Infectious Diseases* 2018.

119. Achilles SL, Mhlanga F, Dezzutti CS, et al. Differences in genital tract immune cell populations and innate cervicovaginal fluid anti-HIV activity among women from Zimbabwe and the United States. *AIDS Research and Human Retroviruses* 2016; **32**(Supplement 1): 78.

120. Austrian K, Hewett PC, Soler-Hampejsek E, Bozzani F, Behrman JR, Digitale J. Adolescent Girls Empowerment Programme: research and evaluation mid-term technical report, 2016.

121. Baird SJ, Garfein RS, McIntosh CT, Ozler B. Effect of a cash transfer programme for schooling on prevalence of HIV and herpes simplex type 2 in Malawi: a cluster randomised trial. *Lancet (London, England)* 2012; **379**(9823): 1320-9.

122. Birdthistle IJ, Floyd S, Machingura A, Mudziwapasi N, Gregson S, Glynn JR. From affected to infected? Orphanhood and HIV risk among female adolescents in urban Zimbabwe. *AIDS (London, England)* 2008; **22**(6): 759-66.

123. Bradley J, Floyd S, Piwowar-Manning E, et al. Sexually transmitted bedfellows: Exquisite association between HIV and herpes simplex virus type 2 in 21 communities in Southern Africa in the HIV prevention trials network 071 (PopART) Study. *Journal of Infectious Diseases* 2018; **218**(3): 443-52.

124. Chatterjee K, Dandara C, Gyllensten U, et al. A Fas gene polymorphism influences herpes simplex virus type 2 infection in South African women. *Journal of medical virology* 2010; **82**(12): 2082-6.

125. Cowan FM, Pascoe SJS, Langhaug LF, et al. The Regai Dzive Shiri Project: A cluster randomised controlled trial to determine the effectiveness of a multi-component community-based HIV prevention intervention for rural youth in Zimbabwe - Study design and baseline results. *Tropical Medicine and International Health* 2008b; **13**(10): 1235-44.

126. Crucitti T, Jespers V, Mulenga C, Khondowe S, Vandepitte J, Buve A. Non-sexual transmission of Trichomonas vaginalis in adolescent girls attending school in Ndola, Zambia. *PloS one* 2011; **6**(1): e16310.

127. Delany-Moretlwe S, Jentsch U, Weiss H, et al. Comparison of focus HerpesSelect and Kalon HSV-2 gG2 ELISA serological assays to detect herpes simplex virus type 2 antibodies in a South African population. *Sexually transmitted infections* 2010; **86**(1): 46-50.

128. Fearon E, Wiggins RD, Pettifor AE, et al. Associations between friendship characteristics and HIV and HSV-2 status amongst young South African women in HPTN-068. *Journal of the International AIDS Society* 2017; **20**(4).

129. Ferrand RA, Munaiwa L, Matsekete J, et al. Undiagnosed HIV infection among adolescents seeking primary health care in Zimbabwe. *Clinical infectious diseases : an official publication of the Infectious Diseases Society of America* 2010a; **51**(7): 844-51.

130. Ferrand RA, Bandason T, Musvaire P, et al. Causes of acute hospitalization in adolescence: burden and spectrum of HIV-related morbidity in a country with an early-onset and severe HIV epidemic: a prospective survey. *PLoS medicine* 2010b; **7**(2): e1000178.

131. Francis SC, Mthiyane TN, Baisley K, et al. Prevalence of sexually transmitted infections among young people in South Africa: A nested survey in a health and demographic surveillance site. *PLoS medicine* 2018; **15**(2): e1002512.

132. Glynn JR, Crampin AC, Ngwira BM, Ndhlovu R, Mwanyongo O, Fine PE. Herpes simplex virus type 2 trends in relation to the HIV epidemic in northern Malawi. *Sexually transmitted infections* 2008; **84**(5): 356-60.

133. Glynn JR, Kayuni N, Gondwe L, Price AJ, Crampin AC. Earlier menarche is associated with a higher prevalence of Herpes simplex type-2 (HSV-2) in young women in rural Malawi. *eLife* 2014; **3**: e01604.

134. Gray GE, Allen M, Moodie Z, et al. Safety and efficacy of the HVTN 503/Phambili study of a clade-B-based HIV-1 vaccine in South Africa: a double-blind, randomised, placebo-controlled test-of-concept phase 2b study. *The Lancet Infectious diseases* 2011; **11**(7): 507-15.

135. Gregson S, Mason PR, Garnett GP, et al. A rural HIV epidemic in Zimbabwe? Findings from a population-based survey. *International journal of STD & AIDS* 2001; **12**(3): 189-96.

136. Gwanzura L, Chigonda TG, Mvere D, De Villiers DM, Siziya S, Mason PR. The prevalence of Herpes simplex virus type-2 infection in blood donors in Harare, Zimbabwe. *The Central African journal of medicine* 2002; **48**(3-4): 38-42.

137. Hazel A, Foxman B, Low BS. Herpes simplex virus type 2 among mobile pastoralists in northwestern Namibia. *Annals of human biology* 2015; **42**(6): 543-51.

138. Kapina M, Reid C, Roman K, et al. HIV incidence rates and risk factors for urban women in Zambia: Preparing for a microbicide clinical trial. *Sexually transmitted diseases* 2009; **36**(3): 129-33.

139. Kenyon C, Colebunders R, Buve A, Hens N. Partner-concurrency associated with herpes simplex virus 2 infection in young South Africans. *International journal of STD & AIDS* 2013; **24**(10): 804-12.

140. Kharsany ABM, McKinnon LR, Lewis L, et al. Population prevalence of sexually transmitted infections in a high HIV burden district in KwaZulu-Natal, South Africa: Implications for HIV epidemic control. *International Journal of Infectious Diseases* 2020; **98**: 130-7.

141. Kjetland EF, Gwanzura L, Ndhlovu PD, et al. Herpes simplex virus type 2 prevalence of epidemic proportions in rural Zimbabwean women: association with other sexually transmitted infections. *Archives of gynecology and obstetrics* 2005; **272**(1): 67-73.

142. Kurewa NE, Mapingure MP, Munjoma MW, Chirenje MZ, Rusakaniko S, Stray-Pedersen B. The burden and risk factors of Sexually Transmitted Infections and Reproductive Tract Infections among pregnant women in Zimbabwe. *BMC infectious diseases* 2010; **10**: 127.

143. Luseno WK, Hallfors DD, Cho H, et al. Use of HIV and HSV-2 biomarkers in sub-saharan adolescent prevention research: a comparison of two approaches. *The journal of primary prevention* 2014; **35**(3): 181-91.

144. Mbizvo EM, Msuya Sia E, Stray-Pedersen B, Chirenje MZ, Munjoma M, Hussain A. Association of herpes simplex virus type 2 with the human immunodeficiency virus among urban women in Zimbabwe. *International journal of STD & AIDS* 2002; **13**(5): 343-8.

145. Menezes LJ, Pokharel U, Sudenga SL, et al. Patterns of prevalent HPV and STI co-infections and associated factors among HIV-negative young Western Cape, South African women: the EVRI trial. *Sexually transmitted infections* 2018; **94**(1): 55-61.

146. NIMH Collaborative HIV/STD Prevention Trial Group. Sexually transmitted disease and HIV prevalence and risk factors in concentrated and generalized HIV epidemic settings. *AIDS (London, England)* 2007; **21 Suppl 2**: S81-90.

147. Pascoe SJ, Langhaug LF, Mavhu W, et al. Poverty, food insufficiency and HIV infection and sexual behaviour among young rural Zimbabwean women. *PloS one* 2015; **10**(1): e0115290.

148. Price J, Pettifor A, Selin A, et al. The association between perceived household educational support and HIV risk in young women in a rural South African community (HPTN 068): A cross sectional study. *PloS one* 2019; **14**(1): e0210632.

149. Wand H, Ramjee G. The relationship between age of coital debut and HIV seroprevalence among women in Durban, South Africa: A cohort study. *BMJ Open* 2012; **2**(1): 000285.

150. Cowan FM, Hargrove JW, Langhaug LF, et al. The appropriateness of core group interventions using presumptive periodic treatment among rural Zimbabwean women who exchange sex for gifts or money. *Journal of acquired immune deficiency syndromes (1999)* 2005; **38**(2): 202-7.

151. Abdool Karim SS, Abdool Karim Q, Kharsany AB, et al. Tenofovir Gel for the Prevention of Herpes Simplex Virus Type 2 Infection. *The New England journal of medicine* 2015; **373**(6): 530-9.

152. Balkus JE, Brown ER, Hillier SL, et al. Oral and injectable contraceptive use and HIV acquisition risk among women in four African countries: A secondary analysis of data from a microbicide trial. *Contraception* 2016; **93**(1): 25-31.

153. Barnabas SL, Dabee S, Passmore JS, et al. Converging epidemics of sexually transmitted infections and bacterial vaginosis in southern African female adolescents at risk of HIV. *International journal of STD & AIDS* 2018; **29**(6): 531-9.

154. Benjamin RJ, Busch MP, Fang CT, et al. Human immunodeficiency virus-1 infection correlates strongly with herpes simplex virus-2 (genital herpes) seropositivity in South African and United States blood donations. *Transfusion* 2008; **48**(2): 295-303.

155. Gust DA, Soud F, Hardnett FP, et al. Evaluation of sexual risk behavior among study participants in the TDF2 PrEP study among heterosexual adults in Botswana. *Journal of Acquired Immune Deficiency Syndromes* 2016; **73**(5): 556-63.

156. Mavedzenge SN, Weiss HA, Montgomery ET, et al. Determinants of differential HIV incidence among women in three southern African locations. *Journal of acquired immune deficiency syndromes (1999)* 2011; **58**(1): 89-99.

157. Wand H, Ramjee G. Evaluating HIV prevention efforts using semiparametric regression models: Results from a large cohort of women participating in an HIV prevention trial from KwaZulu-Natal, South Africa. *Journal of the International AIDS Society* 2013; **16**: 18589.

158. Sutcliffe S, Taha TE, Kumwenda NI, Taylor E, Liomba GN. HIV-1 prevalence and herpes simplex virus 2, hepatitis C virus, and hepatitis B virus infections among male workers at a sugar estate in Malawi. *Journal of acquired immune deficiency syndromes (1999)* 2002; **31**(1): 90-7.

159. Lewis DA, Chirwa TF, Msimang VM, Radebe FM, Kamb ML, Firnhaber CS. Urethritis/cervicitis pathogen prevalence and associated risk factors among asymptomatic HIV-infected patients in South Africa. *Sexually transmitted diseases* 2012; **39**(7): 531-6.

160. Lowe S, Mudzviti T, Mandiriri A, et al. Sexually transmitted infections, the silent partner in HIV-infected women in Zimbabwe. *Southern African Journal of HIV Medicine* 2019; **20 (1) (no pagination)**(a849).

161. Kufa T, Radebe F, Maseko V, Puren A, Kularatne R. Medical Male Circumcision and Associations Among Sexually Transmitted Infections Service Attendees. *AIDS and behavior* 2020; **24**(5): 1422-31.

162. Rabenau HF, Lennemann T, Kircher C, et al. Prevalence- and gender-specific immune response to opportunistic infections in HIV-infected patients in Lesotho. *Sexually transmitted diseases* 2010; **37**(7): 454-9.

163. Varo R, Buck WC, Kazembe PN, Phiri S, Andrianarimanana D, Weigel R. Seroprevalence of CMV, HSV-2 and HBV among HIV-Infected Malawian Children: A Cross-sectional Survey. *Journal of tropical pediatrics* 2016; **62**(3): 220-6.

164. Chen CY, Ballard RC, Beck-Sague CM, et al. Human immunodeficiency virus infection and genital ulcer disease in South Africa: the herpetic connection. *Sexually transmitted diseases* 2000; **27**(1): 21-9.

165. Esber A, Rao N, Norris A, et al. Intravaginal Practices and Prevalence of Sexual and Reproductive Tract Infections Among Women in Rural Malawi. *Sexually transmitted diseases* 2016; **43**(12): 750-5.

166. Hoyo C, Hoffman I, Moser BK, et al. Improving the accuracy of syndromic diagnosis of genital ulcer disease in Malawi. *Sexually transmitted diseases* 2005; **32**(4): 231-7.

167. Kularatne RS, Muller EE, Maseko DV, Kufa-Chakezha T, Lewis DA. Trends in the relative prevalence of genital ulcer disease pathogens and association with HIV infection in Johannesburg, South Africa, 2007-2015. *PloS one* 2018; **13**(4): e0194125.

168. Lewis DA, Pillay C, Mohlamonyane O, et al. The burden of asymptomatic sexually transmitted infections among men in Carletonville, South Africa: implications for syndromic management. *Sexually transmitted infections* 2008; **84**(5): 371-6.

169. Lewis DA, Marsh K, Radebe F, Maseko V, Hughes G. Trends and associations of Trichomonas vaginalis infection in men and women with genital discharge syndromes in Johannesburg, South Africa. *Sexually transmitted infections* 2013; **89**(6): 523-7.

170. Mhlongo S, Magooa P, Muller EE, et al. Etiology and STI/HIV coinfections among patients with urethral and vaginal discharge syndromes in South Africa. *Sexually transmitted diseases* 2010; **37**(9): 566-70.

171. Morse SA, Trees DL, Htun Y, et al. Comparison of clinical diagnosis and standard laboratory and molecular methods for the diagnosis of genital ulcer disease in Lesotho: association with human immunodeficiency virus infection. *The Journal of infectious diseases* 1997; **175**(3): 583-9.

172. O'Farrell N, Morison L, Moodley P, et al. High-risk sexual behaviour in men attending a sexually transmitted infection clinic in Durban, South Africa. *Sexually transmitted infections* 2007; **83**(7): 530-3.

173. Paz-Bailey G, Sternberg M, Puren AJ, et al. Improvement in healing and reduction in HIV shedding with episodic acyclovir therapy as part of syndromic management among men: a randomized, controlled trial. *The Journal of infectious diseases* 2009; **200**(7): 1039-49.

174. Phiri S, Zadrozny S, Weiss HA, et al. Etiology of genital ulcer disease and association with HIV infection in Malawi. *Sexually transmitted diseases* 2013; **40**(12): 923-8.

175. Zimba TF, Apalata T, Sturm WA, Moodley P. Aetiology of sexually transmitted infections in Maputo, Mozambique. *Journal of infection in developing countries* 2011; **5**(1): 41-7.

176. Chattopadhyay K, Williamson AL, Hazra A, Dandara C. The combined risks of reduced or increased function variants in cell death pathway genes differentially influence cervical cancer risk and herpes simplex virus type 2 infection among black Africans and the Mixed Ancestry population of South Africa. *BMC cancer* 2015; **15**: 680.

177. Anaedobe CG, Ajani TA. Co-infection of herpes simplex virus type 2 and HIV infections among pregnant women in Ibadan, Nigeria. *Journal of Global Infectious Diseases* 2019; **11**(1): 19-24.

178. Ashley-Morrow R, Nollkamper J, Robinson NJ, Bishop N, Smith J. Performance of focus ELISA tests for herpes simplex virus type 1 (HSV-1) and HSV-2 antibodies among women in ten diverse geographical locations. *Clinical microbiology and infection : the official publication of the European Society of Clinical Microbiology and Infectious Diseases* 2004; **10**(6): 530-6.

179. Behanzin L, Diabate S, Minani I, et al. Decline in HIV Prevalence among Young Men in the General Population of Cotonou, Benin, 1998-2008. *PloS one* 2012; **7**(8): e43818.

180. Cisse BC, Zaba F, Meite S, et al. Seroprevalence of herpes simplex virus 2 infection among pregnant women in urban health training Yopougon-Attie (Cote D’ivoire). *Academic Journals* 2015; **6**(3): 17-21.

181. Eltom MA, Mbulaiteye SM, Dada AJ, Whitby D, Biggar RJ. Transmission of human herpesvirus 8 by sexual activity among adults in Lagos, Nigeria. *AIDS (London, England)* 2002; **16**(18): 2473-8.

182. Kane CT, Diawara S, Ndiaye HD, et al. Concentrated and linked epidemics of both HSV-2 and HIV-1/HIV-2 infections in Senegal: public health impacts of the spread of HIV. *International journal of STD & AIDS* 2009; **20**(11): 793-6.

183. Katz I, De Luca F, Dzudzor B, et al. Seroprevalences of autoantibodies and anti-infectious antibodies among Ghana's healthy population. *Scientific reports* 2020; **10**(1): 2814.

184. Kirakoya-Samadoulougou F, Nagot N, Defer MC, et al. Epidemiology of herpes simplex virus type 2 infection in rural and Urban Burkina Faso. *Sexually transmitted diseases* 2011; **38**(2): 117-23.

185. Lagarde E, Congo Z, Meda N, et al. Epidemiology of HIV infection in urban Burkina Faso. *International Journal of STD and AIDS* 2004; **15**(6): 395-402.

186. Mawak JD, Dashe N, Atseye AB, Agabi YA, Zakeri H. Seroprevalence and co-infection of herpes simplex virus type 2 and human immunodeficiency virus in Nigeria. *Shiraz E Medical Journal* 2012; **13**(1): 33-9.

187. Patnaik P, Herrero R, Morrow RA, et al. Type-specific seroprevalence of herpes simplex virus type 2 and associated risk factors in middle-aged women from 6 countries: the IARC multicentric study. *Sexually transmitted diseases* 2007; **34**(12): 1019-24.

188. Shaw M, van der Sande M, West B, et al. Prevalence of herpes simplex type 2 and syphilis serology among young adults in a rural Gambian community. *Sexually transmitted infections* 2001; **77**(5): 358-65.

189. Thomas JO, Herrero R, Omigbodun AA, et al. Prevalence of papillomavirus infection in women in Ibadan, Nigeria: a population-based study. *British journal of cancer* 2004; **90**(3): 638-45.

190. Walraven G, Scherf C, West B, et al. The burden of reproductive-organ disease in rural women in The Gambia, West Africa. *Lancet (London, England)* 2001; **357**(9263): 1161-7.

191. Aho J, Koushik A, Coutlee F, Diakite SL, Rashed S. Prevalence of HIV, human papillomavirus type 16 and herpes simplex virus type 2 among female sex workers in Guinea and associated factors. *International Journal of STD and AIDS* 2014; **25**(4): 280-8.

192. Dada AJ, Ajayi AO, Diamondstone L, Quinn TC, Blattner WA, Biggar RJ. A serosurvey of Haemophilus ducreyi, syphilis, and herpes simplex virus type 2 and their association with human immunodeficiency virus among female sex workers in Lagos, Nigeria. *Sexually transmitted diseases* 1998; **25**(5): 237-42.

193. Low AJ, Clayton T, Konate I, et al. Genital warts and infection with human immunodeficiency virus in high-risk women in Burkina Faso: a longitudinal study. *BMC infectious diseases* 2011; **11**(1): 20.

194. Nagot N, Ouedraogo A, Ouangre A, et al. Is sexually transmitted infection management among sex workers still able to mitigate the spread of HIV infection in West Africa? *Journal of Acquired Immune Deficiency Syndromes* 2005; **39**(4): 454-8.

195. Traore IT, Meda N, Hema NM, et al. HIV prevention and care services for female sex workers: Efficacy of a targeted community-based intervention in Burkina Faso. *Journal of the International AIDS Society* 2015; **18**(1): 20088.

196. Wade AS, Kane CT, Diallo PA, et al. HIV infection and sexually transmitted infections among men who have sex with men in Senegal. *AIDS (London, England)* 2005; **19**(18): 2133-40.

197. Camara M, Seydi M, Dieye TN, et al. Association between herpes simplex virus type 2 and HIV-1 in a population of married couples from Dakar, Senegal. *International journal of STD & AIDS* 2012; **23**(11): 810-4.

198. Odebisi-Omokanye M, Udeze A, Akanbi K, Jimoh N, Imam M. Serosurvey of Herpes Simplex Virus type-2 infection among HIV Infected Individuals Accessing a Secondary Health Care Facility in Kwara State, North Central Nigeria. *Nig J Pure & Appl Sci* 2017; **30**(2).

199. Yunusa T, Haruna S, Garba H. Seroprevalence of Herpes Simplex virus among human immunodeficiency virus-positive patients in resource-limited setting. *Journal of Global Infectious Diseases* 2019; **11**(3): 107-11.

200. Aryee EA, Bailey RL, Natividad-Sancho A, Kaye S, Holland MJ. Detection, quantification and genotyping of Herpes Simplex Virus in cervicovaginal secretions by real-time PCR: a cross sectional survey. *Virology journal* 2005; **2**(1): 61.

201. Charpentier C, Koyalta D, Ndinaromtan M, et al. Distribution of HIV-1 and HSV-2 epidemics in Chad revealing HSV-2 hot-spot in regions of high-risk HIV spread. *Journal of infection in developing countries* 2011; **5**(1): 64-7.

202. Eis-Hubinger AM, Nyankiye E, Bitoungui DM, Ndjomou J. Prevalence of herpes simplex virus type 2 antibody in Cameroon. *Sexually transmitted diseases* 2002; **29**(11): 637-42.

203. Ozouaki F, Ndjoyi-Mbiguino A, Legoff J, et al. Genital shedding of herpes simplex virus type 2 in childbearing-aged and pregnant women living in Gabon. *International journal of STD & AIDS* 2006; **17**(2): 124-7.

204. Volpi A, Sarmati L, Suligoi B, Montano M, Rezza G, Andreoni M. Correlates of human herpes virus-8 and herpes simplex virus type 2 infections in Northern Cameroon. *Journal of medical virology* 2004; **74**(3): 467-72.

205. Longo JDD, Simaleko MM, Diemer HSC, Gresenguet G, Brucker G, Belec L. Risk factors for HIV infection among female sex workers in Bangui, Central African Republic. *PloS one* 2017; **12**(11): e0187654.

206. Nzila N, Laga M, Thiam MA, et al. HIV and other sexually transmitted diseases among female prostitutes in Kinshasa. *AIDS (London, England)* 1991; **5**(6): 715-21.

207. LeGoff J, Gresenguet G, Gody C, et al. Performance of the BioPlex 2200 multiplexing immunoassay platform for the detection of herpes simplex virus type 2 specific antibodies in African settings. *Clinical and Vaccine Immunology* 2011; **18**(7): 1191-3.

208. Mbopi-Keou FX, Gresenguet G, Mayaud P, et al. Interactions between herpes simplex virus type 2 and human immunodeficiency virus type 1 infection in african women: Opportunities for intervention. *Journal of Infectious Diseases* 2000; **182**(4): 1090-6.

209. Vandepitte JM, Malele F, Kivuvu DM, et al. HIV and other sexually transmitted infections among female sex workers in Kinshasa, Democratic Republic of Congo, in 2002. *Sexually transmitted diseases* 2007; **34**(4): 203-8.

210. Evidence for Contraceptive Options and HIV Outcomes (ECHO) Trial Consortium. HIV incidence among women using intramuscular depot medroxyprogesterone acetate, a copper intrauterine device, or a levonorgestrel implant for contraception: a randomised, multicentre, open-label trial. *Lancet (London, England)* 2019; **394**(10195): 303-13.

211. Marrazzo JM, Ramjee G, Richardson BA, et al. Tenofovir-based preexposure prophylaxis for HIV infection among African women. *New England Journal of Medicine* 2015; **372**(6): 509-18.

212. Heffron R, Donnell D, Rees H, et al. Use of hormonal contraceptives and risk of HIV-1 transmission: a prospective cohort study. *Lancet Infectious Diseases* 2012; **12**(1): 19-26.

213. LeGoff J, Weiss HA, Gresenguet G, et al. Cervicovaginal HIV-1 and herpes simplex virus type 2 shedding during genital ulcer disease episodes. *AIDS (London, England)* 2007; **21**(12): 1569-78.

214. Ahmed HJ, Mbwana J, Gunnarsson E, et al. Etiology of genital ulcer disease and association with human immunodeficiency virus infection in two tanzanian cities. *Sexually transmitted diseases* 2003; **30**(2): 114-9.

215. Gray RH, Serwadda D, Tobian AA, et al. Effects of genital ulcer disease and herpes simplex virus type 2 on the efficacy of male circumcision for HIV prevention: Analyses from the Rakai trials. *PLoS medicine* 2009; **6**(11): e1000187.

216. Kamya MR, Nsubuga P, Grant RM, Hellman N. The high prevalence of genital herpes among patients with genital ulcer disease in Uganda. *Sexually transmitted diseases* 1995; **22**(6): 351-4.

217. Makasa M, Buve A, Sandoy IF. Etiologic pattern of genital ulcers in Lusaka, Zambia: has chancroid been eliminated? *Sexually transmitted diseases* 2012; **39**(10): 787-91.

218. Mayaud P, Nagot N, Konate I, et al. Effect of HIV-1 and antiretroviral therapy on herpes simplex virus type 2: A prospective study in African women. *Sexually transmitted infections* 2008; **84**(5): 332-7.

219. Mungati M, Machiha A, Mugurungi O, et al. The Etiology of Genital Ulcer Disease and Coinfections With Chlamydia trachomatis and Neisseria gonorrhoeae in Zimbabwe: Results From the Zimbabwe STI Etiology Study. *Sexually transmitted diseases* 2018; **45**(1): 61-8.

220. Nilsen A, Kasubi MJ, Mohn SC, Mwakagile D, Langeland N, Haarr L. Herpes simplex virus infection and genital ulcer disease among patients with sexually transmitted infections in Dar es Salaam, Tanzania. *Acta dermato-venereologica* 2007; **87**(4): 355-9.

221. Oni AA, Adu FD, Ekweozor CC, Bakare RA. Herpetic urethritis in male patients in Ibadan. *West African journal of medicine* 1997; **16**(1): 27-9.

222. Paz-Bailey G, Rahman M, Chen C, et al. Changes in the etiology of sexually transmitted diseases in botswana between 1993 and 2002: Implications for the clinical management of genital ulcer disease. *Clinical Infectious Diseases* 2005; **41**(9): 1304-12.

223. Pickering JM, Whitworth JA, Hughes P, et al. Aetiology of sexually transmitted infections and response to syndromic treatment in southwest Uganda. *Sexually transmitted infections* 2005; **81**(6): 488-93.

224. Tanton C, Weiss HA, Rusizoka M, et al. Long-term impact of acyclovir suppressive therapy on genital and plasma HIV RNA in Tanzanian women: a randomized controlled trial. *The Journal of infectious diseases* 2010; **201**(9): 1285-97.

225. Lai W, Chen CY, Morse SA, et al. Increasing relative prevalence of HSV-2 infection among men with genital ulcers from a mining community in South Africa. *Sexually transmitted infections* 2003; **79**(3): 202-7.
